# Pan-cancer subtyping in a 2D-map shows substructures that are driven by specific combinations of molecular characteristics

**DOI:** 10.1038/srep24949

**Published:** 2016-04-25

**Authors:** Erdogan Taskesen, Sjoerd M. H. Huisman, Ahmed Mahfouz, Jesse H. Krijthe, Jeroen de Ridder, Anja van de Stolpe, Erik van den Akker, Wim Verheagh, Marcel J. T. Reinders

**Affiliations:** 1 Delft Bioinformatics Lab (DBL), Delft University of Technology, Delft, 2628CD, the Netherlands; 2 Division of Image Processing, Department of Radiology, Leiden University Medical Center, Leiden, the Netherlands; 3 Precision and decentralized Diagnostics, Philips Research, Eindhoven, the Netherlands

## Abstract

The use of genome-wide data in cancer research, for the identification of groups of patients with similar molecular characteristics, has become a standard approach for applications in therapy-response, prognosis-prediction, and drug-development. To progress in these applications, the trend is to move from single genome-wide measurements in a single cancer-type towards measuring several different molecular characteristics across multiple cancer-types. Although current approaches shed light on molecular characteristics of various cancer-types, detailed relationships between patients within cancer clusters are unclear. We propose a novel multi-omic integration approach that exploits the joint behavior of the different molecular characteristics, supports visual exploration of the data by a two-dimensional landscape, and inspection of the contribution of the different genome-wide data-types. We integrated 4,434 samples across 19 cancer-types, derived from TCGA, containing gene expression, DNA-methylation, copy-number variation and microRNA expression data. Cluster analysis revealed 18 clusters, where three clusters showed a complex collection of cancer-types, squamous-cell-carcinoma, colorectal cancers, and a novel grouping of kidney-cancers. Sixty-four samples were identified outside their tissue-of-origin cluster. Known and novel patient subgroups were detected for Acute Myeloid Leukemia’s, and breast cancers. Quantification of the contributions of the different molecular types showed that substructures are driven by specific (combinations of) molecular characteristics.

With rapidly increasing availability of novel therapeutic options, like targeted therapies for tumor-driving signal transduction pathways and revolutionary immunotherapies, there is an urgent clinical need to match these therapies to specific groups of patients, in order to maximize patient benefit. Conventionally, cancer subtyping, prognosis assessment, and therapy choice for cancer patients are based on standard histopathology, such as pathological stainings for KI67, ER, PR and Her2 in the case of breast cancer^1^, or identifying *EGFR*, *BRAF*, and *KRAS* mutations in colorectal or lung cancer^2^. High throughput technologies, such as microarrays and next-generation sequencing, have opened new possibilities for biomarker discovery and cancer subtyping, by moving from single gene analysis to an analysis encompassing the whole genome and/or transcriptome^3^,^4^. For instance, transcriptional breast cancer signatures have been associated with clinical outcome^5^. Remarkably, patient groups identified using either genomic mutations or expression signatures often show poor concordance. This is for example apparent in Acute Myeloid Leukemias where the largest group of patients have normal karyotypes with point mutations (e.g, *FLT3ITD*, *NPM1, IDH1*/*IDH2* or *KRAS/NRAS*) and do not cluster on mutation status using gene expression profiles^6^. A lack of cluster robustness across different molecular data-types complicates treatment choice. Hence, there is a need for integrative analyses of genome-wide datasets across different molecular data-types to reach a unified and more robust cancer subtyping.

Besides analyzing each molecular data-type separately, integration of transcriptomic architectures with other types of molecular data (e.g., single-nucleotide polymorphisms^7^, DNA-methylation^8^,^9^, or copy-number variation^10^) is heavily researched and has shown to improve patient characterization and subtyping. The simple hypothesis is that each molecular characteristic provides a different perspective of the same disease, and integration of complementary data should give more comprehensive insights in the disease state. Therefore, many studies step away from single data-type analysis and develop methods to integrate various data-types to improve disease characterisation^11^,^12^,^13^,^14^.

State-of-the-art approaches to improve cancer characterization and patient subtyping incorporate multiple molecular genome-wide data-types, but also multiple cancerous tissues (pan-cancer). It is believed that such a pan-cancer approach can reveal common mechanisms of cancerous cells that could substantially improve the success of targeted therapies. A special case is the group of metastatic cancers identified as samples with unknown primary origin, which have been shown to account for 3% to 5% of all cancers^15^,^16^. The Cancer Genome Atlas project^17^ (TCGA) provides multiple types of molecular data for 34 different cancerous tissues. Exploiting multi-omic pan-cancer data does, however, require novel data integration methods^18^,^19^.

Data integration can be performed in three ways: early (concatenation-based), intermediate (transformation-based) and late (model-based) stage integration^18^. Recently, for patient subtyping on multi-omic pan-cancer data, the late stage integration approach (data integration after analyses of each molecular data-type separately) was used^20^. Although many novel insights were gained, and it outperformed other methods^21^,^22^, the approach still describes the patient relationships for each of the measured molecular characteristics separately, while a majority vote decides on the final clustering. Alternatively, an early or intermediate stage integration approach would be more powerful, because it can capture interactions between different molecular data-types. As an example, in AML it has been shown that modeling the joint effect of different molecular characteristics has advantages in subtype discovery and prediction^8^,^9^. Hence, it seems more reasonable to integrate data earlier. Such an approach is methodologically more complex, since the molecular data-types have different data distributions, and types with an excessive number of features can dominate the results.

Patient relationships are commonly described using tree-based clustering approaches, with the result that relationships between similar patients are difficult to interpret. As an example, it has been shown that hundreds of squamous-like-tissue samples cluster strongly together^20^, but it is unclear whether there is any substructure among these patient samples. Insights in the similarities between similar samples are important to further refine subgroups of patients with similar genome-wide characteristics. Alternatively, patient samples can be visualized in a low-dimensional map (e.g. in two dimensions) by employing, for instance, a principal component analysis (PCA) and exploring the similarities between the samples in the 2D-map^14^,^23^. However, PCA puts emphasis on finding dissimilar samples, rather than similar samples^24^. Therefore, another approach should be followed when studying the relationships between samples within and across cancer subtypes.

We propose a novel omic integration method, called MEREDITH, which is a hybrid of an early stage (concatenation-based) and intermediate stage (transformation-based) integration approach. Our approach allows for visualization and analysis of samples in a 2D-map that emphasizes similarities between similar samples, and quantifies the contribution of each molecular data-type across the genome-wide dataset. We collectively analyzed 4,434 specimens from 19 cancers by integrating same-sample measurements across different molecular characteristics, and demonstrate that MEREDITH detects known global pan-cancer clusters as well as within-tissue-of-origin clusters. We report putative novel cancer subtypes, and identify patients with unknown primary tumor of origin, for which we show that only a subset has significantly poor prognosis. Finally, we systematically quantified the contribution of each molecular data-type in the multi-omic approach, demonstrating the importance of each data-type for pan-cancer and within-tissue-of-origin cancer subtyping. Software is freely available on request and the cancer map can be explored interactively at http://pancancer-map.ewi.tudelft.nl.

## Results

### MEREDITH: multi-omic data integration approach

**Figure 1:**
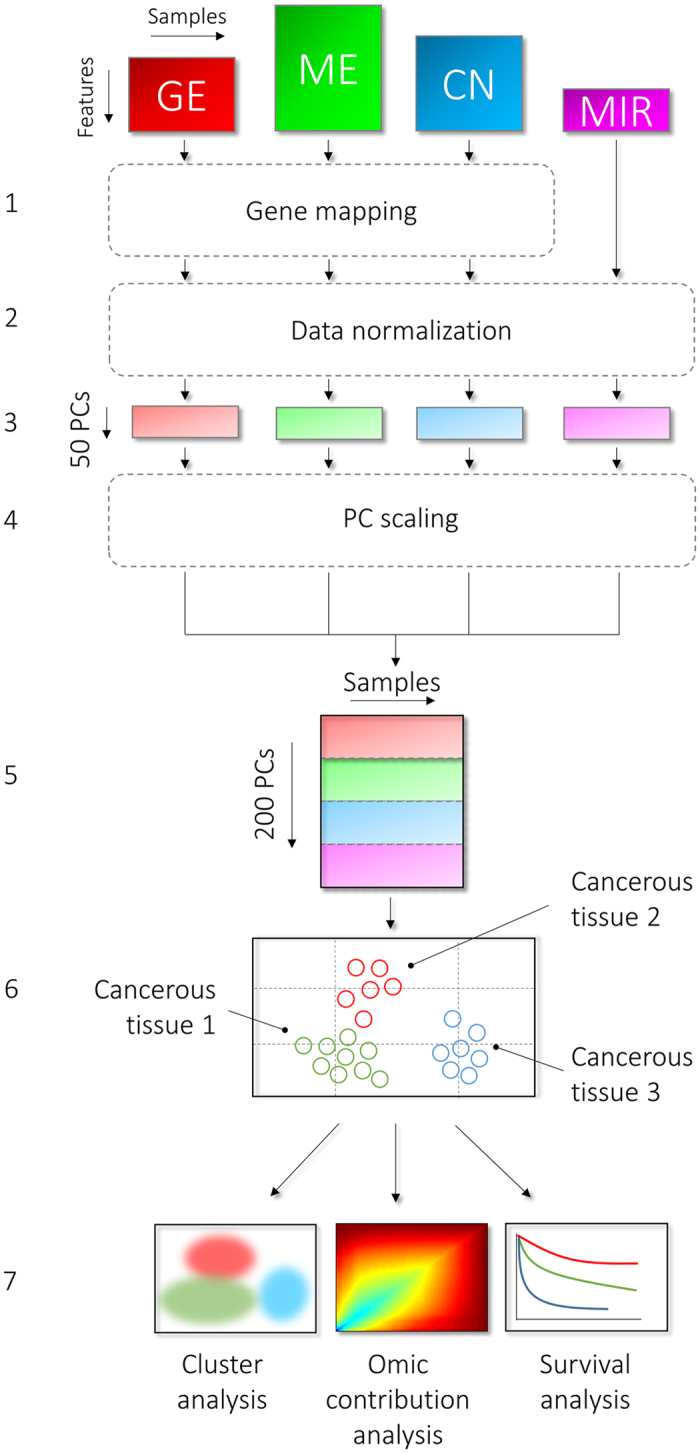
Schematic overview of the multi-omic approach. The seven principal steps in MEREDITH to process and integrate genome-wide data of the four molecular data-types (GE, ME, CN, and MIR). Step 1. Mapping of features to the corresponding genes, 2. PCA per platform for an initial dimensionality reduction, 3. The retained the 50 PCs with the highest eigenvalues for each data set, 4. PC scaling, 5. Concatenation of the PCs per data set, 6. Mapping to a two-dimensional multiplatform map using t-distributed stochastic neighborhood embedding, 7. Cluster analysis, analysis of the contribution of different molecular types, and survival analysis can subsequently be applied.

**Figure 2:**
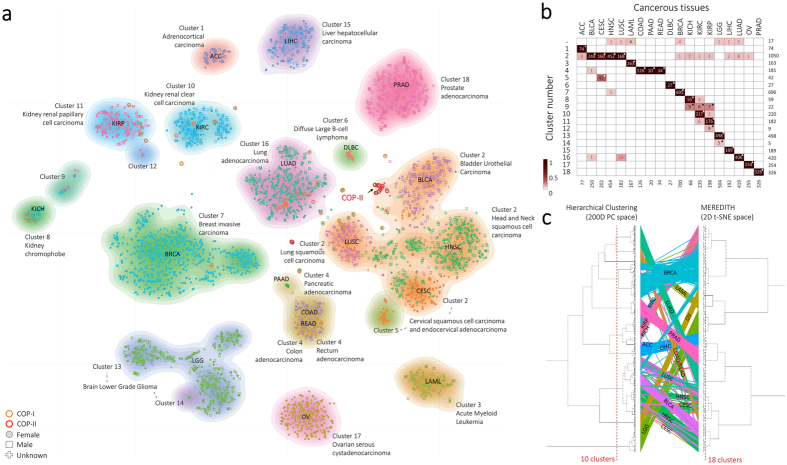
Patient-sample projection in a two-dimensional map illustrating the cancer-landscape. (**a**) Projection of the 4,434 patient cancer samples using MEREDITH. Each point, either being square (male), dot (female), or plus (gender unlabeled), is a sample which is colored based on the cancer-type label (19 cancer-types in total). The clustering of cancer samples is illustrated by the 18 differently colored density maps. (**b**) Heat map depicting the clustering of cancer samples versus cancer-types. A star indicates significant overrepresentation of samples from a specific cancer-type in a cluster, whereas the colored squares depict the percentages of cancer samples in a particular cluster. Red colored number depicts the COP samples. (**c**) A comparison of hierarchical clustered samples using the 200PC space versus MEREDITH and hierarchical clustering. An edge links the sample ID positions as clustered by the HC and t-SNE approaches. Edge colors are based on the cancer-tissue labels.

We devised a novel multi-omic data integration approach (MEREDITH) to identify similarities among 4,434 patients taken from The Cancer Genome Atlas (TCGA)^17^ across 19 cancer-types based on genome-wide measurements of four different molecular characteristics: gene expression (GE; 18,882 features), DNA-methylation (ME; 11,429 features), copy-number variation (CN; 23,638 features) and microRNA expression (MIR; 467 features). MEREDITH is able to find similarities between samples across all molecular data-types simultaneously in seven principal steps (Fig. 1). First, features of each molecular data-type are mapped to the corresponding genes and a principal component analysis (PCA) is applied per dataset of a particular molecular type for an initial dimensionality reduction. We retained the 50 PCs with the highest eigenvalues for each data-type, which is a parameter setting that users can change (explained variance for GE, ME, CN and MIR is 74%, 66%, 73%, and 89% respectively). Note that similar results are obtained when retaining the PCs that explain 95% of the variance, see supplement for more details (Fig. S1). The contributions per molecular data-type are scaled using the total variance of each of their respective set of 50 PCs to ensure that the final integrated result is not dominated by a single data-type (See supplementary method for more details). Next, the reduced features for each of the four types of molecular data are concatenated, resulting in a 200 dimensional (200D) space in which the samples are represented. Then, samples are mapped to a two-dimensional multi-omic map (MO-map, Fig. 2a) using t-distributed stochastic neighborhood embedding (t-SNE)^25^. Here we used the fast Barnes-Hut t-SNE^26^, which non-linearly retains local similarities between samples instead of focusing on the similarities between dissimilar samples. The resulting multi-omic map allows for subsequent analyses, such as cluster analysis, contribution analysis of the different molecular data-types, or patient survival analysis. It should be noted that the MO-map is determined by a stochastic mapping, resulting in different mappings for different initializations. We used the solution with the lowest Kullback–Leibler divergence after running Barnes-Hut t-SNE 1000 times. In addition, the axes in the MO-map are meaningless since samples are positioned in such a way that “only” their similarities are being preserved.

### MO-map reflects original sample distributions faithfully

We first quantified the conservation of local similarities among samples in the MO-map using a measure of local similarity reflecting the percentage of overlapping neighboring samples (Methods section and Supplementary Fig. S2). The (2D) MO-map showed high local similarity (64%) with the 200D space, spanned by the PCs of the different types of molecular data (Supplementary Fig. S3b), indicating that the output of the t-SNE algorithm accurately represents smaller distances of the samples in their high-dimensional space. We also compared the MO-map to that of a higher order t-SNE map (6D), and detected strong preservation of neighboring relationships (81%, Supplementary Fig. S3c). In contrast, inspection of the first two principal components (with the highest eigenvalue) yielded a low local similarity compared to the MO-map (39%, Supplementary Fig. S3d) and poor separation of the 19 cancer-tissues (Supplementary Fig. S3a). Note that the principal components represent a linear combination of all features based on the four omic data sets (loadings are demonstrated in supplementary Fig. S2a). This indicates that the local structure of the data cannot be preserved by mapping the 200D data to a 2D space using a linear method like PCA (44% similarity, Supplementary Fig. S3e) that equally weighs similar and dissimilar samples.

### Unsupervised pan-cancer clustering detects known and novel data structures

Unsupervised clustering of the MO-map revealed 18 clusters (Fig. 2a, based on DBSCAN with the Davies-Bouldin index score for selecting the number of clusters, more details in Methods section). The 18 clusters are subsequently associated with the cancer-type labels by assessing the overrepresentation of each cancer-type within each cluster (Fig. 2b, *P* < 0.001) Out of the 18 clusters, 15 showed a near one-to-one relationship with the cancer-tissue-of-origin (Fig. 2b). Moreover, we confirmed the near one-to-one relationship by testing whether the t-SNE distances between samples from the same tissue-of-origin are significantly smaller than randomly chosen samples (*P* < 1 × 10^−4^, Supplementary Fig. S4, Methods section). Patient clustering on the 2D MO-map created by MEREDITH showed high accordance with conventional clustering based on the 200D PCA space (cophenetic correlation^27^ of 0.46, Fig. 2c), and is in line with previously published analyses of TCGA data^16^,^20^. The three detected clusters without a clear one-to-one relationship with tissue-of-origin contain a complex mixture of cancer samples from various cancer-types (Fig. 2, panel a,b), i.e., the squamous-like cancer samples (Cluster 2: BLCA, CESC, HNSC and LUSC), colorectal and pancreatic cancers (Cluster 4: COAD, READ and PAAD), and a putative novel subtype of kidney-related cancer samples (Cluster 9: KICH, KIRC and KIRP). Interestingly, although the detection of the cluster with the squamous-like-type cancer-tissue samples is consistent with previous results^20^, the MO-map clearly demonstrates that these cancer-tissues are not completely inseparable (Fig. 2a). Examination of Cluster 4, in which all samples are derived from the gastro-intestinal tract, revealed that especially COAD and READ cancer samples are placed together (Fig. 2a). The co-clustering of these two groups is in line with literature, where they are described as colorectal cancers^28^, for which mutations in the Wnt signaling pathway drive tumorigenesis^29^,^30^,^31^,^32^.

Although there is strong overlap between clusters obtained from the MO-map and those obtained by hierarchical clustering in the 200D space, there are differences in the structure of the dendrogram (Fig. 2c). In particular, specific samples destroy the balance of the dendrogram in the 200D space (in fact only 10 clusters are detected, based on optimizing the Davies-Bouldin index score, whereas there are 19 cancer-tissues based on the MO-map). Hence, by retaining the local similarity between samples (which is done by the t-SNE step in MEREDITH), we balance the general distribution of samples, improving their subsequent analysis. As a result, the MO-map supports visual exploration of both the distribution of clusters and of the individual samples.

To investigate the value of MEREDITH further, we evaluated the results of different clustering algorithms (DBSCAN^33^, Hierarchical Clustering, k-means, and Mixture of Gaussians^34^), and PCA between the samples in the low-dimensional MO-map and in their original high-dimensional representation (200D). MEREDITH showed highest tissue enrichment for 9 out of 19 cancer tissues (Fig. S5, Table S1), and lowest Davies-Bouldin scores (Fig. S5). This demonstrates that a reduction of data complexity, by a transformation step of samples into a low-dimensional space, is beneficial for follow-up analysis.

### MO-map gives insight in joint behaviour of the different molecular data-types

**Figure 3:**
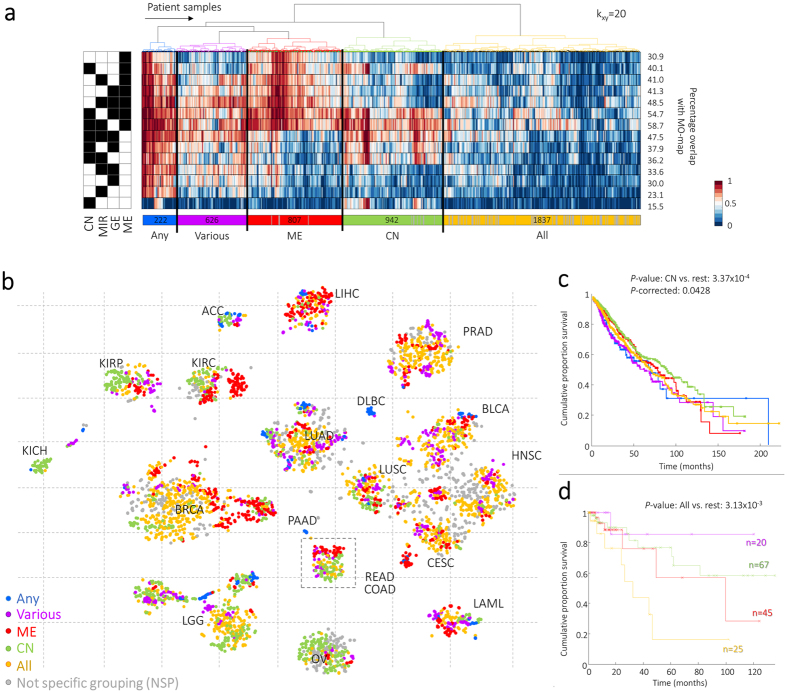
Single data-type versus integrated approach. (**a**) The fourteen alternative molecular data-type combinations with their unique sample projection are pairwise compared with the MP-approach, for *k*_*xy*_ = 20-nearest neighbours. The percentage of overlap with the MO-map that is derived for each of the 4,434 patient samples is subsequently hierarchically clustered based on Euclidean distance and ward linkage. Percentages depicted at the right column describe the averages per molecular data-type over all samples. (**b**) Colouring the MO-map based on the cluster labels to demonstrate the contribution of combinations of data-types. (**c**) Kaplan-Meier plot of overall survival for the different genomic profiles in (**a**). *P*-corrected demonstrate the *P*-value after correcting for the covariates age, sex and for cancer-tissue type since survival rates per cancer are quite different. (**d**) Kaplan-Meier plot of overall survival for the different genomic profiles for only COAD/READ samples.

To determine the contribution of the four molecular data-types in the MO-map, we systematically quantified the overlap of the k(=20)-nearest neighbours per sample in the MO-maps for each of the fourteen possible combinations with the neighbours in the MO-map based on all four molecular data-types (Methods section). By hierarchically clustering the overlap-percentage per sample for every combination, samples with a similar overlap-percentage across the different contributions of molecular data-types are grouped together, and thereby define a common genomic profile (depicted in Fig. 3a).

The Blue Cluster (denoted as any-profile) contains 222 samples and shows on average the same 20-nearest neighbors across all MO-maps; i.e. these samples group consistently together, no matter which combination of molecular data-types is taken. Cancer tissues that were significantly overrepresented in this group are DLBC, PAAD, ACC, LAML, and LGG (*P* < 0.001). For the Purple Cluster (n = 626) samples do not show consistent genomic patterns but rather a mixture of various combinations of molecular data-types for BLCA, LAML, and PRAD (*P* < 0.001). The Red (n = 807) and Green (n = 942) clusters include a ME or CN genomic profile, respectively. Hence, the clustering of these samples in the MO-map is mainly driven by either of these two molecular data-types. Samples with a ME genomic profile are significantly overrepresented with the cancer-types COAD, BRCA, KIRC, and LIHC (*P* < 0.001), whereas samples with the CN genomic profile are associated with COAD, KICH, KIRC, KIRP, LGG, and OV (*P* < 0.001). Interestingly, a deeper examination of the colorectal samples (COAD and READ), which initially showed no clear structure in the sample distribution (Fig. 2a), reveals now the underlying genomic profiles (Fig. 3a, within dashed box). This suggests that the sample distribution is based on differences in DNA-methylation and Copy number changes.

The Yellow Cluster contains 1,837 samples (41%) for which the local neighborhoods are unique to the MO-map based on all four molecular data-types (denoted as all-profile). These samples are located mainly in the “center” of the MO-map (Fig. 3b) and are significantly overrepresented among the cancer-types BRCA, HNSC, PRAD and BLCA (*P* < 0.001).

To evaluate the consistency of the genomic profiles of the samples, we reran MEREDITH with 100 random initializations to derive 100 MO-maps. The average overlap of the 100 MO-maps with the final MO-map is 78% based on the 20-nearest neighborhood (Supplementary Fig. S6). 502 samples (11.3%) had no consistent 20-nearest neighborhood, and these samples were mainly located in the Yellow Cluster (448/502, Fig. 3a,b indicated with the grey lines, and dots respectively). The remaining 1,389 (31.3%) samples in the Yellow Cluster are thus the result of a complex mix of molecular features (as their neighborhood is preserved and are thus not the result of mapping variation).

Interestingly, when comparing the overall survival (OS) of samples with a specific genomic profile to that of all other samples, we detected that samples from the Green Cluster (combinations with CN) have significant better OS in the log-rank test (*P* = 3.37 × 10^−4^, Fig. 3c). These results remained significant in the in the Cox proportional hazard ratio model where we corrected for the confounders age, sex and the six cancer-types that are significantly associated with CN (*P*cox = 0.0428, HR = 0.831, 95% CI 0.69–0.99). Moreover, examination of exclusively the colorectal samples (Fig. 3b, dashed rectangle), showed that samples from the Yellow Cluster (based on all data-types) are associated with significantly inferior OS (*P* = 3.13 × 10^−3^, Fig. 3d). Together these results show that different combinations of molecular data-types contribute differently to the (sub)grouping in the MO-map, emphasizing the importance to analyze the distribution of samples after integrating the data over the different omic-datasets using MEREDITH.

### MEREDITH reveals known and novel structures for Acute Myeloid Leukemia

Acute Myeloid Leukemia (AML) is a heterogenic hematological malignancy that can be dissected in subtypes that have prognostic value and determine therapy choice. In general, patients with AML are separated into three groups, i.e., patients with abnormal karyotype (e.g., translocations, t(8;21), t(15;17) or inversions inv(16)), normal karyotype (usually with point mutations in the genes *FLT3ITD*, *NPM1, IDH1*), and complex karyotype (with multiple (cyto)genetic abnormalities, e.g., 3q, 7q, and trisomy 8). These three groups are known to have good, intermediate and poor survival, respectively^6^,^35^,^36^.

**Figure 4:**
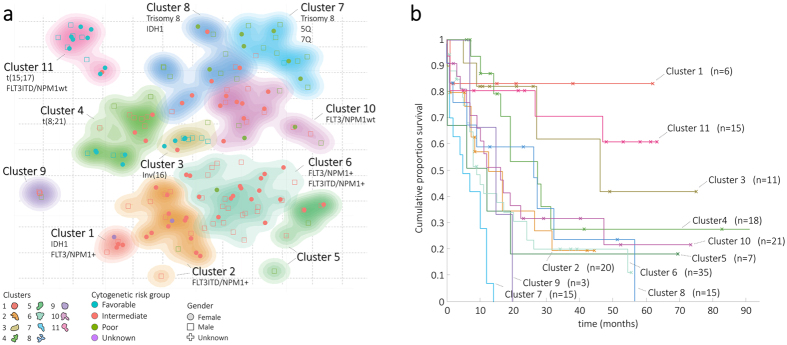
Subtyping of Acute Myeloid Leukemia patient samples. (**a**) MO-map representing the 167 Acute Myeloid Leukemia samples based on MEREDITH. Each point, either being square (male), dot (female), or plus (gender unlabeled), is a sample and colored according the cytogenetic risk-groups. AML subtype labels are depicted when significantly overrepresented with the cluster (hypergeometric test *P* < 0.001). Clustering of cancer samples is illustrated by the 11 differently colored density maps. (**b**) Kaplan-Meier plot for overall survival (OS) for the determined cluster labels.

Unsupervised clustering of the 167 AML patients, using MEREDITH, resulted in 11 clusters. Nine of these clusters were significantly associated with (cyto)genetic labels (*P* < 0.05, Fig. 4a, Supplementary Table S2). Only two clusters are not significantly enriched for any of the (cyto)genetic labels but rather are associated with FAB-classification^37^ M5 (Cluster 5), and M6 (Cluster 9).

The association of the clusters with (cyto)genetic labels demonstrates that the complex behavior of the known AML subtypes is accurately be captured in the MO-map (Fig. 4a). Overall, the grouping of samples is in line with previous results^6^,^9^,^35^, e.g., clustering of samples with: *i*) t(8;21), Cluster 4; *ii*) t(15;17), Cluster 11; *iii*) inv(16), Cluster 13; and *iv*) complex karyotype in Cluster 7. In addition, we could discern samples with different combinations of *FLT3*/*NPM1* and *IDH1* mutations, known to be associated with clinical outcome^38^,^39^. In addition, samples in Cluster 1, with *IDH1* mutations and the genotype of mutated *NPM1* without *FLT3ITD*, were recently discovered by solely looking at the genotypes^39^. Interestingly, our MO-map shows that *IDH1* mutants can be further dissected in two different clusters based on the four molecular data-types (Cluster 1 and 8), which appear to have different OS (Fig. 4b). Although we cannot show its significance using the log-rank test (*P* = 0.09), further investigation with larger sets of samples may provide better characterization of this specific subgroup of AML.

### Integrating multiple molecular data-types identifies the subtypes of AML cancers

To assess whether the 11 detected AML clusters (Fig. 4a) can also be discovered when analyzing the GE, ME, CN, and MIR genome-wide datasets separately, we used MEREDITH to analyze each of the four omic-datasets separately. For each molecular data-type we follow the same clustering procedure and cut the tree in 11 clusters (same number as when using MEREDITH on the four data-types combined), Each cluster for each data-type is then tested for significant overlap with any of the MEREDITH clusters (*P* < 0.001, Supplementary Table S2). There was no individual molecular data-type that could recover all 11 clusters identified by MEREDITH, indicating that the four molecular data-types contain complementary information. Note that the solutions provided by the four individual data-types are not necessarily incorrect but are rather different, or an incomplete perspective of the same disease. There was only one cluster, i.e., samples with a complex karyotype (Cluster 7) that grouped consistently together over all four molecular data-types. This is of interest as these samples are known to have a multitude of mutations, which appear to present themselves across all four molecular characteristics (Supplementary Table S2). In contrast, samples that grouped in the novel Cluster 9 are only seen by the CN data, highlighting that some differences have a highly specific origin. We also analyzed whether MEREDITH outperforms various PCA reductions (2D/3D/4D, and 200D) in terms of cluster enrichment for the AML subtypes. We detected that 6 out of 12 AML groups showed highest cluster enrichment using MEREDITH being comparable to the derived from the 200D PCA space (Table S3).

### MEREDITH correctly identifies known subtype structure for breast cancers

Patients with breast cancer can be categorized into three general groups: 1) triple negative breast cancer dominated by basal type breast cancer (i.e., estrogen receptor negative (ER−), progesterone receptor negative (PR−), and human epidermal growth factor receptor-2 negative (HER-2); 2) the HER-2 subtype (ER−/PR−/HER2+); and 3) ER/PR positive breast cancer (ER+/PR+), which can be further subtyped based on mRNA profiling into luminal A (ER+/PR+/HER2−) and luminal B (ER+/PR+/HER2+)^5^. Despite the variation in prognosis between the different subtypes within each group, therapy choices are tuned to the three basic groups. It was already shown before that data integration revealed novel insights^40^,^41^,^42^, however unknown are the results in subtyping when integrating mRNA, DNA-methylation, microRNA and Copy number changes.

**Figure 5:**
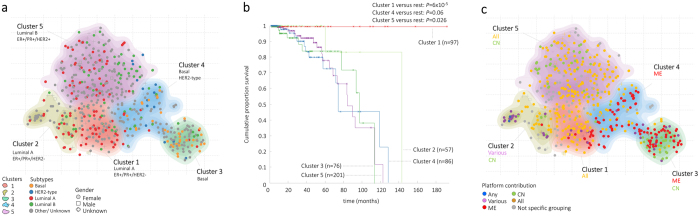
Subtyping of Breast Cancer patient samples. (**a**) MO-map representing the 563 Breast cancer samples based on MEREDITH. Each point, either being square (male), dot (female), or plus (gender unlabeled), is a sample and colored according the subtypes of breast cancer. The clustering of cancer samples is illustrated by the five differently colored density maps. (**b**) Kaplan-Meier plot for overall survival (OS) for the five cluster labels. (**c**) Colouring the samples in the MO-map based on the genomic profiles (Fig. 2a). Breast cancer subtypes and genomic profile labels are depicted when significantly overrepresented with the cluster (hypergeometric test *P* < 0.001).

Subtype analysis of the 563 breast cancer samples using MEREDITH resulted in five distinct clusters (Fig. 5a) that were significantly associated with the known breast cancer subtypes: *i*) cluster 1 for the Luminal A subtype (*P* = 5.1 × 10^−6^); cluster 2 also for Luminal A (*P* = 6.2 × 10^−4^); *iii*) cluster 3 for the Basal subtype (*P* = 3.9 × 10^−14^); *iv*) cluster 4 for the Basal and HER2-type (*P* = 1.5 × 10^−3^ and *P* = 0.035 respectively); and *v*) cluster 5 for the Luminal B subtype (*P* = 1.6 × 10^−6^). Detailed clinical characteristics can be found in Supplementary Table S4. This clearly demonstrates that breast cancer subtypes can also be captured in the two-dimensional MO-map (Fig. 5a). In terms of overall survival, we detected that samples in Cluster 5 (enriched for Luminal B) have a significant inferior survival compared to samples outside the cluster (*P* = 0.026), with 5-year survival rates of 78% (Fig. 5b). Intriguingly, while both Clusters 1 and 2 include samples that are significantly associated with Luminal A, samples in Cluster 1 showed significantly good survival rates (*P* = 6 × 10^−5^), which was not seen of patients in Cluster 2.

### Integrating multiple molecular data-types identifies subtypes of breast cancers

Similar to our analysis of the AML clusters, we analyzed whether the five breast cancer clusters identified using MEREDITH can be similarly discovered when analyzing the GE, ME, CN, and MIR datasets separately. We detected that the known breast cancer subtypes can be readily detected by analyzing solely gene expression profiles, i.e. 78% overlap between the 20-nearest neighbours of the samples in the gene expression map and the MO-map (based on the four molecular data-types). A cluster analysis revealed that all four data-types could recover the 5 clusters identified by MEREDITH (Fig. 5a, supplementary Table S4 and Fig. S7). However, there is an advantage of incorporating the four different data-types which can especially be seen in Cluster 1 and 2 (Fig. 5c). Both clusters are significantly enriched for Luminal A but their molecular makeup now clearly shows that only a specific set of samples group together when the four omic data sets is used. We also analyzed whether MEREDITH outperforms various PCA reductions (2D/3D/4D, and 200D) in terms of subtype enrichment. We detected that MEREDITH showed highest cluster enrichment for ER/PR/HER2/Luminal A, and Basal subtypes (Table S5).

To determine whether the subtypes of breast cancer (Fig. 5a) are affected by any of the five genomic profiles (Fig. 3a), we systematically assessed their enrichment for each of the breast cancer clusters (Fig. 5c). Samples of the Luminal B subtype were significantly enriched for the Yellow Cluster (i.e., combinations of all data-types, *P* = 2.97 × 10^−4^), whereas samples with the Basal subtype were significantly enriched for the Red Cluster (combinations with ME, *P* = 3.52 × 10^−11^). The associations with the genomic profiles explain the separation of Luminal A samples in the two distinct clusters (Cluster 1 and 2): samples in Cluster 1 are significantly enriched for the Yellow Cluster (all, *P* = 2.67 × 10^−4^), whereas samples in Cluster 2 are enriched for the Purple Cluster (various: *P* = 1.4 × 10^−9^) and Green Cluster (CN: *P* = 1.77 × 10^−3^). Taken together, we demonstrate that specific molecular data-types can drive sample grouping and possible reveal novel subtypes of breast cancer. As an example, subsets of samples in Cluster 2, 3 and 5 (Fig. 5c) are mainly driven by copy-number variations, which is known to be important in subtyping^10^, whereas subsets of samples cluster 3 and 4 are mainly driven by epigenetic changes.

### Samples located outside the primary tissue-of-origin (COPs) can be divided into two distinct groups

Besides samples that fall into the matched tissue-cluster, we also detected samples that do not have a matched genome-wide profile with the cancer-tissue-of-origin cluster (Fig. 2, red and orange circled samples). Such cancer samples have previously been reported as cancer with unknown primary (CUP)^15^,^16^ but were left unnoticed in other pan-cancer studies^20^,^43^,^44^. Our MO-map demonstrates that not all of these cancer-tissue samples are necessarily of unknown primary, but many represent “Cancers Outside their Primary (COP)” because these samples can be located just outside the boundary of the cancer-tissue-of-origin cluster (Fig. 2).

There are 64 COP samples (1.44%, Fig. 2a) across 12 cancer-types (Fig. 2b). Three cancer-types (LUSC, KIRC and LUAD) showed a significant overrepresentation of COPs (*P* = 9.7091e-08, *P* = 1.6054e-04, and *P* = 0.0044, Fig. 2b). In contrast, seven cancer-types included no COPs (CESC, COAD, DLBC, LAML, PAAD, PRAD, and READ).

Because previous studies detected larger numbers of CUPs (3% to 5% of all cancers^15^), we analyzed the percentages of detected COPs across all other combinations of molecular data-types. The COP percentages ranged from 0.25% (CN) to 5.9% (ME) from which 14 samples were consistently detected as being a COP across all combinations. This suggests that not all COP samples may be the result of biological effects but may also be the result of type-specific technical effects.

**Figure 6:**
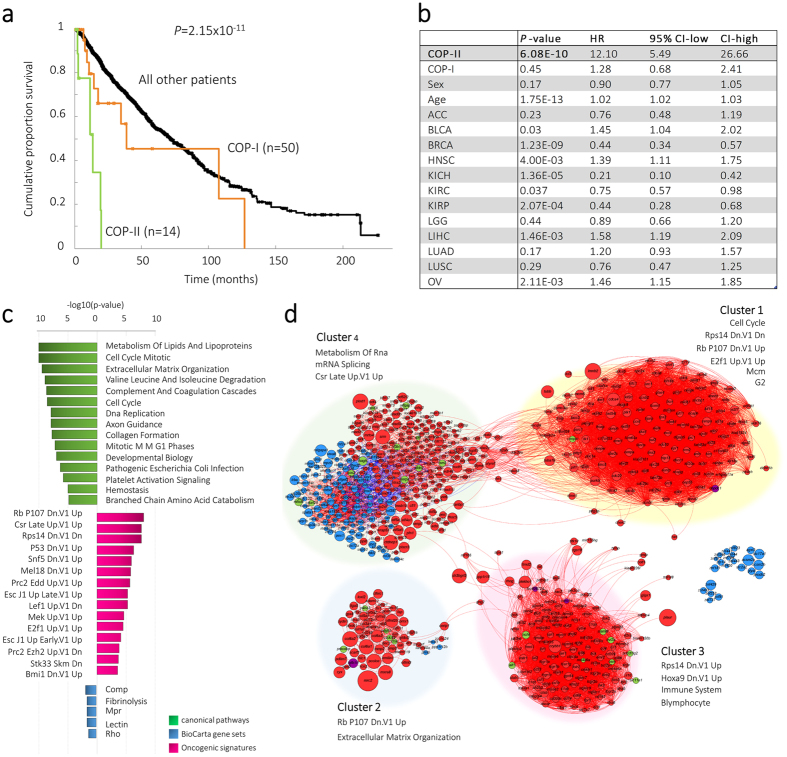
Analysis of the COP cluster with similar profiles. (**a**) Kaplan-Meier survival plot depicted the overall survival (OS) for the COP-I, COP-II and all other remaining samples. The P-value describes the comparison between COP-II samples versus all other samples using the log-rank statistics. (**b**) Cox proportional hazard ratio regression model to determine associations with OS for COP-I and COP-II samples groups after correcting for covariates. The covariates are chosen based on the cancer-tissues that were seen among the COP samples. (**c**) Significantly enriched pathways for the COP-II sample group. (**d**) Co-expression network based on gene expression profiles for the COP-II sample group. Node size depicts the −log10(P-value) for differential expression between COP-II sample group and all other remaining samples. Node color depicts either significant upregulation (red), downregulation (blue), DNA-methylation (green), or copy-number changes (purple). Edges with positive correlation are depicted in red, whereas negative correlations are depicted in blue. Co-expression network is clustered in four distinct groups and significantly enriched pathways for each cluster are depicted.

COP samples were thought to be molecularly similar to the cell type of origin^15^. However, we could dissect the 64 COP samples into two major subgroups. Namely, samples that do cluster with another tissue-of-origin (COP-I), and samples located outside the annotated tissue-of-origin but without genomic similarities to any of the known cancer-type tissue labels (COP-II). The type-II COPs (n = 14) cluster at the edge of the squamous-like-type of cancers (Fig. 2a, Cluster 2), and were detected by running MEREDITH on the 64 COPs in isolation of the other samples (Supplementary Fig. S8a). In addition, the type-II COPs showed higher correlations among each other when analyzing the molecular data-types separately (Supplementary Fig. S8b). Strikingly, these COPs showed strongly reduced survival compared to the type-I COPs (log-rank *P* = 2.15 × 10^−11^, Fig. 6a), which remained significant after correction for potential confounders as listed in Fig. 6b (*P*cox = 6.08 × 10^−10^, HR = 12.1, 95% CI 5.49–26.66). In contrast, the OS of the type-I COPs (n = 50) showed no significant differences compared to all other samples with matched tissue-of-origin (*P* = 0.16, and *P*cox = 0.45, Fig. 6b).

We next analysed whether the 14 type-II COP samples have a specific pathway abnormality or co-expression network by comparing the gene expression profiles to all other samples. We first zero-mean normalized gene expression values per cancer to avoid detecting cancer-specific related gene expression profiles. When comparing the type-II COPs to all other samples, we detected 3,474 significantly differential expressed genes using Limma^45^ and multiple testing correction using Holm^46^ (*P* < 0.05). Pathway analysis on these genes showed association with well-known cancer biomarkers (Fig. 6c), such as a suppressor of retinoblastoma RB-P107 (*P* = 9.88 × 10^−9^), but also P53 (*P* = 5.98 × 10^−7^), LEF1 (*P* = 5.41 × 10^−6^), MEK (*P* = 2.63 × 10^−6^), E2F1 (*P* = 3.54 × 10^−5^), KRAS (*P* = 5.8 × 10^−4^) (detailed information can be found in Supplementary Table S6 and Methods section). We created a co-expression network by computing the pairwise Pearson correlations for the differentially expressed genes (only retaining genes with at least two correlating partners for which the correlation is higher than 0.6). This resulted in four distinct gene clusters (Fig. 6d), all significantly associated with known mechanisms to be disturbed in cancer cells, including: cell cycle pathway, RB-P107, immune system, and mRNA splicing pathways as shown in Clusters 1 to 4, respectively.

## Discussion

We introduce a multi-omic integration approach, MEREDITH, which we applied to integrate four different types of molecular data: mRNA expression, DNA-methylation, microRNA, and copy-number changes. We analyzed 4,434 patients across 19 primary cancer-types and showed a strong similarity between samples with the same tissue-of-origin. Instead of analyzing the molecular data-types separately, as is the case with current state-of-the-art methods such as COCA^20^, MEREDITH creates a 2D MO-map of the samples based on all data-types, taking joint behavior into account. The 2D MO-map represents accurately the sample distributions in the high-dimensional measurement space, can easily be visually explored, and identifies robustly substructures in the data. For example, it has been demonstrated that hundreds of squamous-like-tissue samples cluster together^20^, but with MEREDITH we clearly demonstrate the substructure among these samples. Furthermore, we demonstrate novel grouping of samples in AML, breast cancer and COPs that has not been shown before.

It should be noted that, besides the similarly clustered samples, differences between clustering algorithms in the original space versus the low-dimensional space are also seen (Fig. S5). As an example, samples of the squamous-like-tissue are clustered using MEREDITH, and using hierarchical clustering in the original space (200D). However, when we cluster the low-dimensional space using K-means or hierarchical clustering, it can separate HNSC, CESC, LUSC, and BLCA (Fig. S5). Thus our presented low-dimensional space may also be used for the prediction of these separate cancer-tissues. In general, differences between results when using various clustering algorithms are insurmountable. A final biological interpretation remains an experts task for which our provided MO-map allows a more detailed examination of local substructures across and within the cancer-tissues.

MEREDITH resembles the MFA approach by Tayrac *et al.*^14^. Like them we follow a hybrid data integration where different data-types are merged after an initial principal component analysis for each data-type separately (to weigh their contributions equally). However, after this initial step we use stochastic neighborhood embedding instead of PCA analysis. We argued that for a low-dimensional embedding of the data (like the 2D-map that we propose) it is important to emphasize similarities between samples (as done by t-SNE) instead of emphasizing dissimilarities between samples (as done by PCA and advocated by Tayrac *et al.*). This principal change much better captures the different cancer (sub)types (Fig. 2a), and is representative for the sample distribution in high-dimensional space (supplement Fig. S3b,c), as opposed to using PCA in the second step (supplement Fig. S3a,d,e). Note that the PCA in the initial step captures most of the variability in each of the data-types so that the argument of keeping similar samples together is not relevant for this integration step. Tayrac *et al.* inspected the contributions of the different data-types by looking at the loading factors of the final PCA step. Although insightful, we show how a sample is influenced by molecular data-types by systematically evaluating all possible combinations of genomic data. As such, samples could be categorized into genomic profiles, and substructures can now be explained by their molecular makeup.

From a pan-cancer perspective we show that integrating molecular data-types can better separate cancer-tissue samples in well-defined clusters compared to analyzing only a single molecular data-type. To quantify the separability of all cancer-tissue samples by using a supervised-learning approach, we used each label (cancer-type) of a sample and assessed whether it could be predicted from the label of its closest neighbor. The MO-approach outperformed all single data set analyses (AUC = 0.94, Supplementary Fig. S9). However, slightly worse performance is obtained for GE, ME or MIR separately (AUC ≈ 0.93 for each of these data types), and relatively bad performance when using the CN data (AUC = 0.72). This indicates that for GE, ME and MIR only a small number of patients is misclassified when performing single data-type analysis. This is not the case for copy number changes that appear to be less informative. We speculate that the MO-approach is less sensitive to noise since it integrates over the different data types, and therefore retains patients in the tissue-of-origin cluster, whereas single data-type analysis mistakenly pushes away samples from the tissue-of-origin cluster. Thus combining data types, may not necessarily reveal novel clusters but it may prevent misclassifications. More details about single data type analysis versus MO-analysis can be found in supplementary materials (Single data type analysis versus MO-map). Although samples with similar cancers tend to group together in both the integrated map as well as the individual maps (Supplementary Figs S10–S13), there is an intriguing difference. Samples tend to cluster more densely in the integrated map, whereas samples in individual maps seem to “drift away” from the tissue-of-origin cluster. Nevertheless, it can be beneficial to analyze different combinations of molecular data-types to improve our understanding of the complex relationships between different cancer-tissue samples and their molecular make-up. For example, we detected that colon and rectal tissue samples are tangled together in a cluster. However, an analysis of the contributions of each of the molecular data-types provided insights in the separation between the two cancer-types. To support visual exploration of the contributions of the different data-types, we have made the maps of different combinations of molecular data-types available in an interactive browser at the following website: http://pancancer-map.ewi.tudelft.nl. This is especially useful when visually exploring subtypes of samples together with various sample annotations (gender, age, survival time etc), which we statically demonstrate for breast cancers in Fig. S7.

Initially we found that maps based on DNA-methylation profiles resulted in strong gender-based grouping within tissue-of-origin (Supplementary Fig. S14). The clear distinction between genders was also seen when analyzing copy-number profiles, however, in that case genders were grouped together across-tissue-of-origin (Supplementary Fig. S15). Although gender specific grouping is a known effect, e.g., males do have a tendency toward higher methylation levels^47^, we saw striking differences between DNA-methylation and copy-number changes and the effect on cancer subtyping. These differences were not prominently seen in the PCA-based maps (Supplementary Fig. S16 panel a,b), again showing the benefit of the multi-omic data integration as proposed by MEREDITH. Note that, to avoid gender-specific clustering, removal of features on the X and Y chromosomes is required (details regarding normalization can be found in methods section).

MEREDITH also detected cancers outside their primary of origin (COPs) that were left unnoticed by different studies^20^. The detection of the COPs depends on the type of molecular data being used, but 56 out of the 64 COP samples were detected repeatedly using seven different combinations of molecular data-types. Another finding is that we detected a subgroup of COPs (type-II COPs) that showed significantly reduced OS. This group showed a common cell cycle pathway abnormality which is likely the result of abnormal regulation of genes in the oncogenic pathways, such as RB, E2F1, P53 (Fig. 6d, clusters 2 and 3). This opens the question whether metastatic cancer cells converge to a stage of similarly disturbed biological mechanisms, for example an EMT transition with associated stem cell cancer characteristics and high metastatic potential and therapy resistance characteristics.

MEREDITH successfully identified known cancer-types and subtypes enabling novel insights in patient characterization and subtyping. Further improvements of subtyping may even be possible as our approach is not limited to these four molecular data-types, but it is applicable to any type and combination of genome-wide data.

## Methods

### TCGA data processing

In this study we used 4,434 patients across 19 distinct malignancies from the TCGA consortium^17^ (Fig. 2b). Four high-throughput datasets were used in this study: mRNA expression data (GE), DNA-methylation data (ME), Copy Number variation data (CN), and microRNA expression data (MIR). We left out the mutational data because it is not trivial to map those to gene-based values. For each dataset, Level 3 data was retrieved from TCGA using the TCGA-Assembler^48^. For GE we retrieved the RNAseqV2, RSEM values which were accordingly log2-transformed (to avoid having infinite values, we initially added+1 to all values), followed by a zero-mean normalization per gene. For ME we retrieved the beta values (methylated/(methylated+unmethylated) ratio) for both Illumina 27 k (505 samples) and 450 k (4,169 samples) beadchip. The beta values are subsequently standardized per sample by averaging gene-wise within 1,500 base pairs of the transcriptional-start-site, followed by quantile normalization. This makes sure that the beta values for both beadchips can be used together. We next applied a zero-mean normalization, and PCA normalization to remove technical variation. For microRNA we retrieved the RPM values which were accordingly log2-transformed (with pseudocount of 1), followed by a zero-mean normalization. For CN, copy-number values are transformed by log2(copy-number/2), and centered on 0. For each dataset we removed all genes located on the X and Y chromosome to avoid gender related biases (Supplementary Figs S14 and S15). Features containing missing values among all samples are removed whereas others are imputed using K = 3 nearest neighbor approach. No biases were observed regarding gender (Supplementary Figs S11a and S12a) ethnicity, race, or BCR center types (Supplementary Fig. S17 panel a–c). To avoid bias due to the DNA-methylation array, we used samples measured on the 450K array in the breast cancer analysis.

### Cluster analysis

Density-Based Algorithm for Discovering Clusters (DBSCAN^33^) is employed to define clusters in each of the cluster analysis. DBSCAN is suitable for clustering t-SNE and PCA maps as it is based on the sample density. Clustering cut-off (eps parameter) is chosen by maximizing the silhouette score^49^, and samples were not forced into clusters, instead, at most 10% of the tissues samples can be labelled as ‘non-clustering’.

### Annotation enrichment

Patient characteristics for cancer-tissues were compared using the Mann-Whitney-U test (continuous-variables) and the one sided Fisher exact test (categorical variables). All the association tests are performed using the hypergeometric test, i.e., to assess significance of overrepresentation of the cancer-types and cluster labels.

### Quantification of Local similarity across two maps

To compare the embedding of samples in two different maps, we propose a scale dependent similarity measure. For a pair of maps X and Y, we compare the sets of the, respectively, *k*_*x*_ and *k*_*y*_ nearest neighbours of each sample. We first define the variable *r*^*x*^_*ij*_ as the rank of the distance of sample *j* among all samples with respect to sample *i*, in map X. The nearest neighbor of sample *i* will have rank 1, the second nearest neighbor rank 2, etc. Analogously, *r*^*y*^_*ij*_ is the rank of sample *j* with respect to sample *i* in map Y. Now we define a score on the interval [0, 1], as (eq. 1)


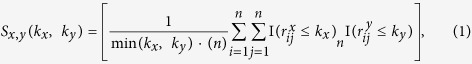


where the variable *n* is the total number of samples, and the indicator function is given by (eq. 2)


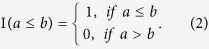


The score *s*_*x,y*_(*k*_*x*_, *k*_*y*_) will have value 1 if, for each sample, all *k*_*x*_ nearest neighbours in map X are also the *k*_*y*_ nearest neighbours in map Y, or vice versa. For the analysis in Fig. 3 we have used *k*_*x*_ = *k*_*y*_ = 20. Other settings of *k*_*x*_ and *k*_*y*_ can be found in the supplement (Fig. S18). Note that a local neighborhood of 20 samples (that we used in our experimental settings) is based on the cancer-tissue with the smallest number of samples (i.e., PAAD). For the analysis in Supplementary Fig. S3, panel b–e we used *k*_*xy*_ = 250 which is the average of the cancer-tissue group size.

### Survival analysis

Outcome measures are assessed using Kaplan-Meier estimates^50^ in a univariate analysis using the log-rank test^51^. Multivariate analyses is performed according the Cox proportional hazard ratio model, where we corrected for the covariates age, sex, and cancer-tissue types when required. The definition of complete remission (CR) and survival endpoints such as overall survival (OS) are provided by TCGA.

### Pathway analysis

Canonical pathways (1,320), Biocarta genesets (217), and oncogenic signatures (189) are utilized from the molecular signature database (MsigDB v4.0) for which we calculated a P-value for the fraction of genes that is significantly detected (either by gene expression, DNA-methylation or copy-number changes), and annotated in the pathway using the hypergeometric test. Multiple test correction is applied according Benjamini and Yukuteli (BY)^52^ and pathways are selected when *P*_BY_ < 0.05.

## Additional Information

**How to cite this article**: Taskesen, E. *et al.* Pan-cancer subtyping in a 2D-map shows substructures that are driven by specific combinations of molecular characteristics. *Sci. Rep.* **6**, 24949

## Supplementary Material

Supplementary Information
