# Author Correction: Pan-cancer subtyping in a 2D-map shows substructures that are driven by specific combinations of molecular characteristics

**DOI:** 10.1038/s41598-018-35518-w

**Published:** 2018-11-23

**Authors:** Erdogan Taskesen, Sjoerd M. H. Huisman, Ahmed Mahfouz, Jesse H. Krijthe, Jeroen de Ridder, Anja van de Stolpe, Erik van den Akker, Wim Verhaegh, Marcel J. T. Reinders

**Affiliations:** 1 Delft Bioinformatics Lab (DBL), Delft University of Technology, Delft, 2628CD The Netherlands; 2 Division of Image Processing, Department of Radiology, Leiden University Medical Center, Leiden, The Netherlands; 3 Precision and decentralized Diagnostics, Philips Research, Eindhoven, The Netherlands

Correction to: *Scientific Reports* 10.1038/srep24949, published online 25 April 2016

This Article contains a typographical error in the spelling of the author Wim Verhaegh, which is incorrectly given as Wim Verheagh.

