# Supplementary material for: Pan-cancer subtyping in a 2D-map shows substructures that are driven by specific combinations of molecular characteristics
Source: Sci Rep. 2016 Apr 25;6:24949. doi: 10.1038/srep24949 (PMC4842960; doi:10.1038/srep24949)
Supplement: Supplementary Information [file srep24949-s1.pdf]

Supplementary materials

Pan-cancer subtyping in a 2D-map shows substructures that are driven by specific combinations of molecular characteristics

Erdogan Taskesen[1], Sjoerd M.H. Huisman[1,2], Ahmed Mahfouz[1,2], Jesse H Krijthe[1], Jeroen de Ridder[1], Anja van de Stolpe[3], Erik van den Akker[1], Wim Verheagh[3], Marcel J.T. Reinders[1*]

Affiliations:
[1] Delft Bioinformatics Lab (DBL), Delft University of Technology, Delft, 2628CD, the Netherlands
[2] Division of Image Processing, Department of Radiology, Leiden University Medical Center, Leiden, the Netherlands
[3] Precision and decentralized Diagnostics, Philips Research, Eindhoven, the Netherlands

ET: E.Taskesen@vu.nl; SH: S.M.H.Huisman@tudelft.nl; AM: A.Mahfouz@tudelft.nl; JHK: J.H.Krijthe@tudelft.nl; JDR: J.deRidder@tudelft.nl; AVDS: anja.van.de.stolpe@philips.com; EVDA: E.B.vandenAkker@tudelft.nl; WV: wim.verhaegh@philips.com; MJTR: M.J.T.Reinders@tudelft.nl

* Corresponding author

MERDITH method

Our proposed method (Fig.1) contains seven principal steps specifically tailored to exploit rich datasets in which samples have been characterized using genome wide measurements. As an input we used four independent genome wide measurements from TCGA. In step 1 we further normalized the level 3 data per data set to zero mean, with additional data specific adjustments as outlined in the methods section. Step 2: Gene mapping: For GE, ME, and CN we mapped features to the accompanying genes as described in methods section. Step 3: Feature extraction: We use canonical principal component analysis (PCA) for an initial dimensionality reduction which is performed per data set and selected are the top 50 principal components (PCs) with highest eigenvalues (explained variance for GE, ME, CN and MIR is 74%, 66%, 73%, and 89% respectively). These contributions per data set are scaled in step 4 on the total variance of each of their respective set of 50 PCs to ensure that the final integrated results are not dominated by a single data set, e.g., $PCnormalized^i = var(PC^i)/(\text{square root}(\text{sum}(var(PC^i))))$ where *i* iterates over the 50 principal components. Step 5: Concatenation: The obtained feature space is a summarized into 200 PCs x 4,434 samples and further reduced using a non-linear dimensionality reduction method, called t-SNE[1], that emphasizes the local similarity between samples. Note that the PCs that cover the 95% explained variance per data set shows similar results (Fig.S1). The t-SNE approach showed to be superior to other feature extraction techniques in the creating of a low-dimensional map based on high-dimensional data[1], which we therefore employed in our approach. All cancer samples can now be projected in a two-dimensional space (step 6), and allows cluster analysis, omic contribution analysis, and corresponding patient survival analysis as depicted in step 7 (Fig.1A).

PCA projections of all cancer-tissues

The use of Principal Component Analysis (PCA) has proven to be a very valuable technique for feature extraction and visualization in genomic research. PCA aims to capture features with largest variance but it lacks in performance for finding local structure in high-dimensional genomic data, when reducing the data to a low (visible) feature space (e.g., 2D or 3D). A disadvantage of PCA is that it preserves the structure in the data by keeping the low-dimensional representations of dissimilar data points far apart. For high-dimensional genomic data, it is important to keep the low-dimensional representations of very similar data points close together, which is typically not possible with a linear mapping approach. Here we systematically evaluate the projections produced by PCA and t-SNE. In general, inspection of the two principal components with highest eigenvalue, based on the integrated data, revealed poor separation between the cancerous tissues (Supplementary Fig.S3C). A cluster analysis grouped all patient samples into two clusters, after following a similar optimization approach as for the t-SNE map. Thus PCA failed

to produce a clear visualization of the complex genomic behavior for the 19 cancerous tissues that the t-SNE mappings offered.

Single data-type analysis versus MO-map

In this section we addressed the question whether the separation/subtyping of cancer-tissue samples using a multi-omic approach is superior compared to that of a single omic analysis. We show the results using three different methods in which we address differences at local and global scales.

First, we addressed the question whether the average t-SNE distance of patients within tissue-of-origin clusters (in Euclidian distance), is significantly smaller compared to the average t-SNE distance of randomly chosen cancer-tissue samples with the same group size (based on 10,000 draw). This quantifies the separation of a cancer-tissue cluster in relation to all cancer-tissue types. For the multi-omic approach (Fig.2A) we already demonstrated significant separation of the cancer-tissue sample clusters ($P<1x10^{-4}$, Supplementary Fig.S4). For the single omic data sets we first projected the cancer samples in a two-dimensional map by using MEREDITH. The resulting 2D-maps (Supplementary Fig.S10-Fig.S13) also produced a significantly smaller t-SNE distance for patients within a tissue-of-origin cluster compared to the average t-SNE distance of randomly chosen samples with the same group size ($P<1x10^{-4}$, Supplementary Fig.S10-Fig.S13). For gene expression data it was previously shown that the use of solely RNA-sequencing data can accurately discriminate various cancer-tissue types in a one versus all supervised approach[2]. We now also demonstrate that genome wide profiles of DNA-methylation, copy-number changes and microRNAs can distinguish between cancer-types.

Finally, we quantified the similarity of the k_x nearest neighboring sample(s) in the MO-map with that of the k_y nearest neighboring sample(s) in the single omic maps. Thus if a single omic map has perfect local similarity with the MO-map, the k_x nearest neighboring sample(s) would fully overlap with k_y, and consequently have the value one. We compared the MO-map to the maps of the four genome wide data sets for the combinations of k_x, and k_y, up to 250 nearest neighbors. As expected, increasing the number of neighbors resulted in higher similarity with the MO-map (Supplementary Fig.S18). This indicates that on a global level (high k_{xy}), cancer-tissue samples are consistently grouped together. Differences however are seen on the local scales (low k_{xy}, Supplementary Fig.S18), which indicates differences in local patient sample orientation (patients have different neighbors across the omic data sets). In general, we detected that the GE map showed highest local similarity with the MP map (77%, Supplementary Fig.S18) when looking at the 250 nearest neighbors (followed by the ME map (74%), MIR map (69%) and finally the CN map (50%, Supplementary Fig.S18).

Taken together, we demonstrate that cancer-tissues are separable by means of genome wide data from each of the four data sets. The MO-approach improved retention of cancer samples in the tissue-of-origin cluster compared to single omic analysis. In terms of subtyping, global patterns of cancer-tissue grouping are comparable between the MO-approach and single omic approach. However, the main differences occur on the local scale, indicating that cancer samples in the MO-approach have different neighbors compared to that of single omic analysis.

DNA-methylation revealed gender specific grouping within tissue-of-origin, whereas Copy Number variation grouped genders across tissue-of-origin

Out of the 19 cancerous tissues, there are 16 tissues with mixed genders, and three gender specific tissues, i.e., CESC, OV are female specific, and PRAD is male specific. For DNA-methylation and Copy Number changes, a gender specific grouping is a known effect, e.g., males do have a tendency toward higher methylation levels[3]. However, we detected a striking difference between ME (Supplementary Fig.S14) and CN (Supplementary Fig.S15) in a pan-cancer analysis; i.e., ME groups genders within-tissue-of-origin whereas CN groups genders across-tissue-of-origin. Nevertheless, for ME there is one exception, we observed gender specific grouping of squamous-like-type cancerous tissues cluster together, i.e., female PAAD patients cluster together with female patients from CESC (Cluster 5), whereas male patients with PAAD cluster together with male patients from HNSC and BLCA (Cluster 3).

We next analysed associations with gender specific clustering per cancer-type. In order to do so, we performed t-SNE followed by DBSCAN per cancer-type. Each cluster is subsequently assessed for associations with gender using the hypergeometric test. Note that the clusters are determined by the silhouette score and therefore not forced into two clusters (representing males and females). For DLBC, PAAD and READ we could not test for gender specific associations due low sample sizes.

For ME, we detected that 11 cancer-types (out of 13 tested) showed significant grouping of gender, meaning that at least two clusters are detected with one enriched for males and one for females. ACC (P=0.0344), LUAD (P=2.09e-67), LGG (P=3.8e-48), LAML (P=2.28e-35), KIRP (P=7.5e-06), KIRC (P=2.34e-16), COAD (P=5.8e-10), HNSC (P=7.8e-53), LIHC, (P=2.3e-07), LUSC (P=6.7e-15), BLCA (P=2.1e-12). Note that the presented P-value is the minimum of the gender associated clusters. Only the detected clusters with kidney Chromophobe (KICH) and breast cancer (BRCA) patient samples are not significantly associated with the grouping of males and females separately. The four cancer-types KIRC, KIRP, LIHC, and LUSC showed multiple significant gender specific clusters. This demonstrates that genders keep clustering together but can be separated based on other DNA-methylation characteristics.

For CN, we also detected 11 cancer-types (out of 13 tested) with significant enrichment for genders; ACC (*P*=0.0365), LUAD (*P*= 4.9532e-89), LGG (*P*= 7.9815e-37), LAML (*P*= 2.3867e-43), KIRP (*P*= 3.3382e-12), KIRC (*P*= 6.7780e-39), COAD (*P*= 1.0700e-18), HNSC (*P*= 6.7541e-62), LIHC, (P= 1.5367e-15), LUSC (P= 4.3421e-16), and BLCA (P= 6.9973e-18). Similar to the DNA-methylation results, only BRCA and KICH showed no separate clusters that were significantly associated with males/females. Interestingly, four cancer-types, COAD, BLCA, LUAD, and LGG showed multiple significantly gender specific clusters. Note that these are four different cancer-types from those detected based on the ME data set.

For microRNA data we detected one cancer-type (HNSC, *P*= 0.0162) with both significant male and female associations to the determined clusters, whereas the gene expression data showed no gender specific associations in the clusters. We finally analyzed the cancer-types based on the integrated omic data sets and detected that Neck squamous cell carcinoma (HNSC, P=0.0229), and Lung adenocarcinoma (LUAD, P=9.8374e-04) were significantly associated with male and female clusters. With these analyses we demonstrate that gender specific grouping for the ME and CN omic data set does dominate the clustering of patient samples when analyzing each of cancer-types separately. There were only two exception BRCA and KICH showed no grouping of males and females in the detected clusters.

Barnes Hut t-SNE

The Barnes Hut t-Distributed Stochastic Neighbor Embedding (t-SNE) algorithm[4] is a computationally efficient variant of the original t-SNE algorithm[1]. The Barnes Hut t-SNE algorithm embeds the high-dimensional data (in our approach the 200 PCs) into a two-dimensional map, and preserves the local structure by using a probabilistic measurement. This means that it finds an arrangement of neighbour patient samples in the low-dimensional space which is representative to that of the high-dimensional space. Moreover, t-SNE convert the distance between two samples in the high-dimensional space into a conditional probability, and subsequently applies a cost function that preserves the computed probabilities in the low-dimensional space. Patient-samples with similar genomic profile will therefore be positioned close together in the map which reflects the proximity in the high-dimensional space, whereas uncorrelated genomic profiles will be distant in the high-dimensional space and will thus also be far apart in the t-SNE map. The perplexity, which is a measure of the effective number of neighbors used to embed each data point, is set on default (i.e., 30) for all mapping that are performed. We used t-SNE to produce 2D projections of the data using Euclidean measures for the distance between data points in the sample space.

Comparison of omic data sets by means of Receiver Operating Curve

To obtain a measure of how well samples of the same cancer-type cluster together in the 2D t-SNE maps, we devised a classification scheme. For each pair of samples, we predicted whether they belong to the same cancer-type, based on their pairwise distance. The sample pairs were ordered by distance in the 2D t-SNE map and an ROC curve was made by scoring true positives (same cancer-type) and false positives (different cancer-type) over this list of pairs.

Quantification of Local similarity across two maps

The scores defined to compare the similarity between two maps are not symmetric because it is possible that, for instance, the 10 nearest neighbors in map X have more overlap with the 20-nearest neighbors in map Y, than the top-20 of map X with the top-10 in map Y.

References

1 Maaten, L. J. P. v. d. & Hinton, G. E. Visualizing High-Dimensional Data Using t-SNE. *Journal of Machine Learning Research* **9**, 2579-2605 (2008).

2 Wei, I. H., Shi, Y., Jiang, H., Kumar-Sinha, C. & Chinnaiyan, A. M. RNA-Seq accurately identifies cancer biomarker signatures to distinguish tissue of origin. *Neoplasia* **16**, 918-927, doi:10.1016/j.neo.2014.09.007 (2014).

3 El-Maarri, O. *et al.* Gender specific differences in levels of DNA-methylation at selected loci from human total blood: a tendency toward higher methylation levels in males. *Hum Genet* **122**, 505-514, doi:10.1007/s00439-007-0430-3 (2007).

4 Maaten, L. v. d. Barnes-Hut-SNE. *arXiv.org* **arXiv:1301.3342** (2013).

Figure legends

Supplementary Fig.S1. Patient-sample projection in a two-dimensional map illustrating the cancer-landscape using the number of principal component that explain the 95% variance. (**a**) Projection of the 4,434 patient cancer samples using MEREDITH by using the number of PCs that explain the 95% of the variance within each data set. In total 3,640 PCs are used for which 1,479 are from gene expression, 1,609 from DNA-methylation, 441 from Copy number variations, and 111 from the microRNA data set. Each point is a sample which is colored based on the cancer-type label (19 cancer-types in total). The clustering of cancer samples is illustrated by the 12 differently colored density maps. (**b**) Heat map depicting the clustering of cancer samples versus cancer-types. A star indicates significant overrepresentation of samples from a specific cancer-type in a cluster, whereas the colored squares depict the percentages of cancer samples in a particular cluster.

Supplementary Fig.S2. Quantification of Local similarity across two maps. Schematic overview to systematically compare local and global differences between two sample projections. For illustration we compare two input maps (x and y) in which each map contains n samples (step 1). The second step is the ranking of samples based on Euclidean distance. The ranks of map x are subsequently compared to the ranks of map y for k_x and k_y nearest neighbours (step 3). The overlap between ranks (step 4), is subsequently summarized in Score: $S_{x,y}(k_x,k_y)$.

Supplementary Fig.S3. Comparisons with maps from principal component analysis. (**a**). PCA projection of the 4,434 patient samples based on the multi-omic data (200D) to 2PCs with highest eigenvalue. The loadings of the PCs are depicted with blue lines. Each patient sample is depicted by a square (male), dot (female), or plus (gender unlabeled), and colored based on one of the 19 cancer-tissues. The density maps are colored according to one of the two cluster labels which are detected as described in Methods section. (**b**) Comparison of the 2D MO-map using MEREDITH versus 200D PCA map. (**c**). Comparison of 2D MO-map versus 6D MO-map using MEREDITH. (**d**) Comparison of the 2D MO-map using MEREDITH versus the 2D PCA map. (**e**). Comparison of 2D PCA map versus 200D PCA map. All comparison between two maps follow the quantification of local similarity across two maps approach for k_{xy}=250 (the average cancer-tissue group size) as described in Methods section.

Supplementary Fig.S4. Separability of cancer-tissues based for the MO-map. The mean distance between samples of a cancer-type is compared to the mean distance between the same numbers of randomly chosen samples (no. of draws is 10,000). The plots show the observed values in red dashed lines and kernel density estimates of the random values in black.

Supplementary Fig.S5 Comparison of cluster algorithms in high (original) dimensional space and low-dimensional space. Cluster algorithms are compared in the high (original 200D) dimensional space and in the low-dimensional space. Panel A-C depicts the Davies Bouldin index (DBindex) score, and cluster enrichment with the cancer-types using: **(a)** HC, **(b)** k-means and **(c)** Mixture of Gaussians respectively. DBSCAN was not able to fit the data in the high data space. Panel D-F depicts the DBindex score, and cluster enrichment in the low-dimensional (2D) space by means of: **(d)** HC, **(e)** k-means, and **(f)** Mixture of Gaussians respectively. Panel G-I depicts the DBindex score, and cluster enrichment in the low-dimensional using HC and: **(g)** PCA-2D, **(h)** PCA-3D, and **(i)** PCA-4D. The red vertical line depicts the optimum number of clusters under the restriction that at least 5 and at most 40 clusters can exists.

Supplementary Fig.S6. Comparison of the MO-map with hundred different initializations. 100 MO-maps with random initialization for patient samples are derived with MEREDITH. The MO-maps are subsequently compared to the final MO-map (Fig.2A) using the local similarity across two maps approach for k_{xy}=20. Each point in the map represents the percentage of overlap per sample between the final MO-map and a MO-map with random start initialization. Values range between [0,..,1] depicting no overlap (blue: 0), up to full overlap (red: 1). The average overlap between the final MO-map and each single MO-map with random start initialization ranges between 75.8 and 84.8. Clustering is performed using Euclidian distance and ward linkage. The Red Cluster consists of samples that are consistently detected with low percentage of overlap compared to the MO-map as a consequence of the random initialization.

Supplementary Fig.S7. Subtyping of Breast Cancer patient samples for GE, ME, CN and MIR data-types separately. Panel (**a**) to (**d**): Analysis of 563 Breast cancer samples using GE, ME, MIR and CN datasets using MEREDITH, respectively Each point is a sample (square: male, dot: female, plus: unlabeled gender) colored on the basis of the subtypes of breast cancer.

Supplementary Fig.S8. Correlation between COP patient samples based on GE, ME, MIR, and CN. (**a**) Projection of the 64 COP samples using MEREDITH. Each point is a COP sample (square: male, dot: female, plus: unlabeled gender) colored on the basis of the cancer-type label. The colored density maps illustrate the two sub-clusters COP-I and COP-II. (**b**) Pairwise correlations between the 64 COP samples based on each of the four molecular data-types (GE, ME, MIR and CN separately). The cells in the heat map are coloured by Pearson correlations values, depicting higher positive (red) or negative (blue) correlations, as indicated by the scale bar.

Supplementary Fig.S9. Supervised comparison between single data-type maps and the integrated MO-map. Supervised comparison between single data-type maps and the integrated MO-map for the

separation of the cancer-tissues by means of the area under the curve (AUC), as follows: For each pair of samples a prediction is made whether they belong to the same cancer-type, based on their pairwise distance. The sample pairs were ordered by distance in the 2D t-SNE map and an ROC curve was made by scoring true positives (same cancer-type) and false positives (different cancer-type) over this list of pairs. Only using the CN dataset (purple line) results in poorest AUC score whereas the MO-map (yellow) results in best AUC score.

Supplementary Fig.S10. Patient-sample projection for GE data respectively. (**a**). Projection of the 4,434 patient samples in 2D for the GE dataset. Each sample (square: male, dot: female, plus: unlabeled gender) is colored on the basis of one of the 19 cancer-tissues. The density map is colored according the cluster label. (**b**). Separability of cancer-tissues for the GE dataset by computing the average distance of samples for a cancer-tissue compared to the average distance between randomly chosen samples using the same group size (no. of draws is 10,000). The plots demonstrate the observed values in red dashed lines and kernel density estimates of the random values in black.

Supplementary Fig.S11. Patient-sample projection for ME data respectively. (**a**). Projection of the 4,434 patient samples in 2D for the ME dataset. Each sample (square: male, dot: female, plus: unlabeled gender) is colored on the basis of one of the 19 cancer-tissues. The density map is colored according the cluster label. (**b**). Separability of cancer-tissues for the ME dataset by computing the average distance of samples for a cancer-tissue compared to the average distance between randomly chosen samples using the same group size (no. of draws is 10,000). The plots demonstrate the observed values in red dashed lines and kernel density estimates of the random values in black.

Supplementary Fig.S12. Patient-sample projection for CN data respectively. (**a**). Projection of the 4,434 patient samples in 2D for the CN dataset. Each sample (square: male, dot: female, plus: unlabeled gender) is colored on the basis of one of the 19 cancer-tissues. The density map is colored according the cluster label. (**b**). Separability of cancer-tissues for the CN dataset by computing the average distance of samples for a cancer-tissue compared to the average distance between randomly chosen samples using the same group size (no. of draws is 10,000). The plots demonstrate the observed values in red dashed lines and kernel density estimates of the random values in black.

Supplementary Fig.S13. Patient-sample projection for MIR data. (**a**). Projection of the 4,434 patient samples in 2D for the MIR dataset. Each sample (square: male, dot: female, plus: unlabeled gender) is colored on the basis of one of the 19 cancer-tissues. The density map is colored according the cluster label. (**b**). Separability of cancer-tissues for the MIR dataset by computing the average distance of samples for a cancer-tissue compared to the average distance between randomly chosen samples using

the same group size (no. of draws is 10,000). The plots demonstrate the observed values in red dashed lines and kernel density estimates of the random values in black.

Supplementary Fig.S14. Patient-sample projection of the genome wide DNA-methylation profiles using all chromosomal features. Projection of the 4,434 patient samples for DNA-methylation profiles without removing features that are located at the X and Y chromosomes. Each point is a sample (square: male, dot: female, plus: unlabeled gender) colored on the basis of the cancer-tissue label (19 cancer-tissues in total). The density map is colored according the cluster label.

Supplementary Fig.S15. Patient-sample projection of the genome wide Copy Number profiles using all chromosomal features. Projection of the 4,434 patient samples for copy-number profiles without removing of features that are located at the X and Y chromosomes. Each point is a sample (square: male, dot: female, plus: unlabeled gender) is colored on the basis of the cancer-tissue label (19 cancer-tissues in total). The density map is colored according the cluster label.

Supplementary Fig.S16. Principal component analysis including all X and Y features. PCA projection using the 2PCs with highest eigenvalue of the 4,434 patient samples based on the (**a**) DNA-methylation levels and (**b**) Copy number changes data without removing of features that are located at the X and Y chromosomes. Each sample (square: male, dot: female, plus: unlabeled gender) is colored on the basis of the sex label. The density map is colored according the cluster label.

Supplementary Fig.S17. MO-map colored on ethnicity, race, DNA-methylation array and tissue source site. Projection of the 4,434 patient samples based on the multi-omic approach. Each point is a sample (square: male, dot: female, plus: unlabeled gender) is colored on the basis of (**a**) Ethnicity, (**b**). Race, (**c**). Tissue source site (TSS), (**d**) DNA-methylation array. The density maps are colored according the cluster label.

Supplementary Fig.S18. Single omic versus MO-map. Comparison of MO-map versus maps derived from the four molecular data-types separately for k_{xy}=[1..250] nearest neighbours. More detail can be found in Methods section (Quantification of Local similarity across two maps), and supplementary material (Single data-type analysis versus MO-map).

Figure S1

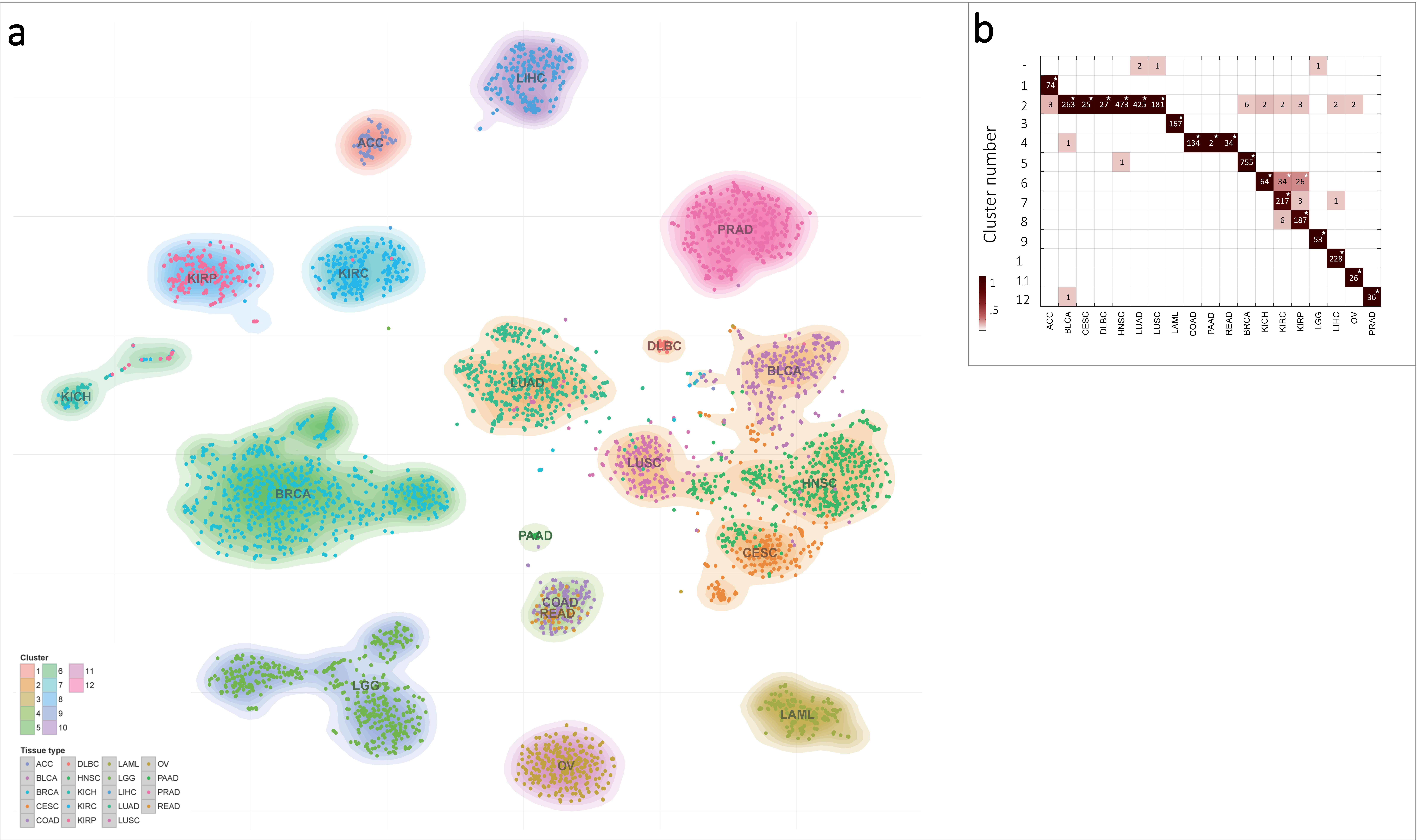
a
LIHC
ACC
PRAD
KIRP
KIRC
KICH
DLBC
LUAD
BLCA
LUSC
HNSC
BRCA
PAAD
CESC
COAD
READ
LGG
LAML
OV
Cluster
1
2
3
4
5
6
7
8
9
10
11
12
Tissue type
ACC
BLCA
BRCA
CESC
COAD
DLBC
HNSC
KICH
KIRC
KIRP
LAML
LGG
LIHC
LUAD
LUSC
OV
PAAD
PRAD
READ
b
Cluster number
ACC
BLCA
CESC
DLBC
HNSC
LUAD
LUSC
LAML
COAD
PAAD
READ
BRCA
KICH
KIRC
KIRP
LGG
LIHC
OV
PRAD
1
.5

Figure S2

1.

2.

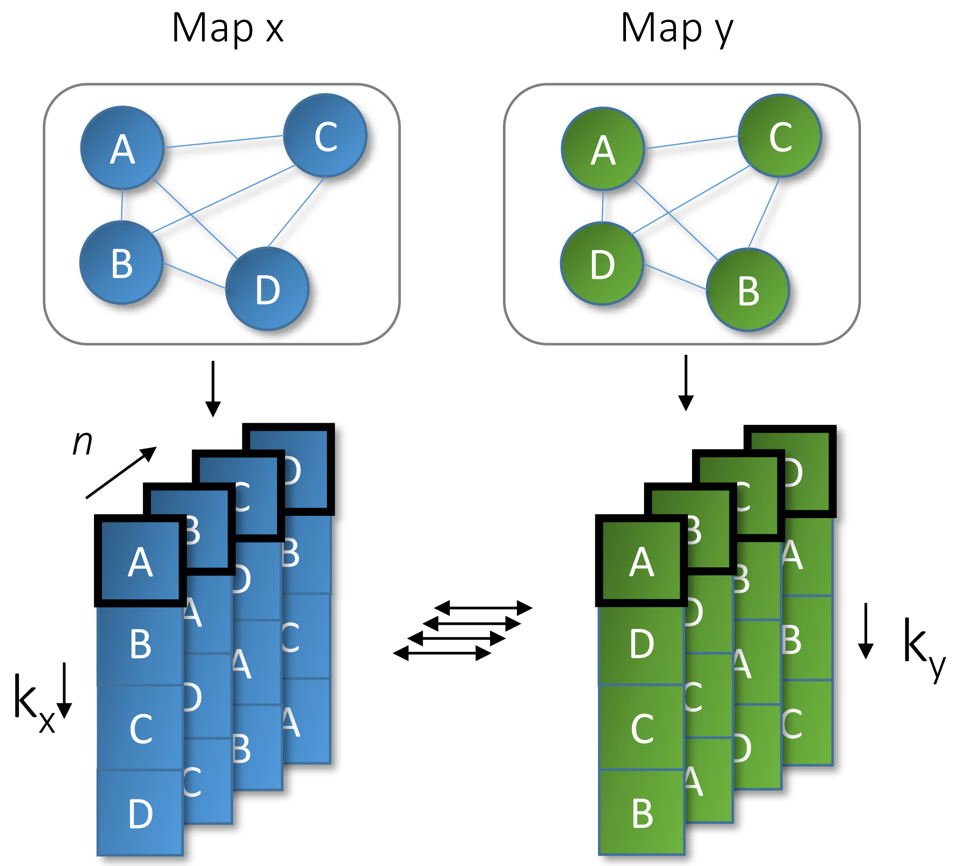

3.

	$k_x,k_y=1$	$k_x,k_y=2$	$k_x,k_y=3$	$k_x=2,k_y=1$	$k_x=1,k_y=2$	$k_x=3,k_y=1$	$k_x=3,k_y=2$	$k_x=2,k_y=3$	$k_x=1,k_y=3$
A	0	1	3	0	0	1	2	2	1
B	0	1	3	0	1	1	2	2	1
C	0	1	3	0	0	1	2	2	1
D	0	1	3	1	0	1	2	2	1
sum	0	4	12	1	1	4	8	8	4
$n \cdot \min(k_x,k_y)$	4	8	12	4	4	4	8	8	4

4.

$S_{x,y}(k_x,k_y)$

k_y \ k_x	1	2	3
3	4/4	8/8	12/12
2	1/4	4/8	8/8
1	0	1/4	4/4

Figure S3

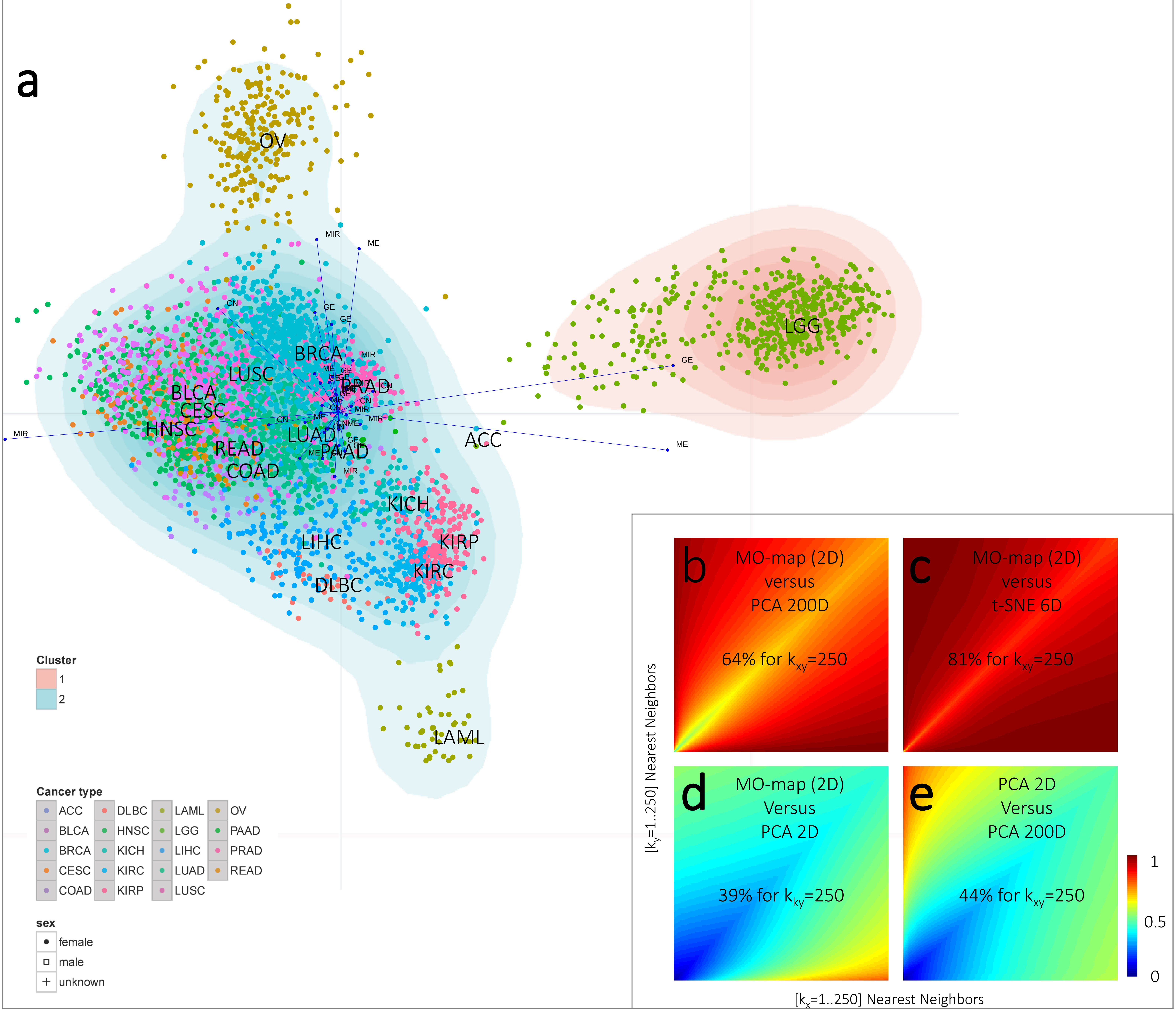

a
OV
LGG
BRCA
LUSC
BLCA
CESC
HNSC
READ
COAD
LUAD
PAAD
PRAD
ACC
KICH
LIHC
KIRP
KIRC
DLBC
LAML
MIR
ME
CN
GE
Cluster
1
2
Cancer type
ACC
BLCA
BRCA
CESC
COAD
DLBC
HNSC
KICH
KIRC
KIRP
LAML
LGG
LIHC
LUAD
LUSC
OV
PAAD
PRAD
READ
sex
female
male
unknown
b
MO-map (2D) versus PCA 200D
64% for k_{xy}=250
c
MO-map (2D) versus t-SNE 6D
81% for k_{xy}=250
d
MO-map (2D) Versus PCA 2D
39% for k_{ky}=250
e
PCA 2D Versus PCA 200D
44% for k_{xy}=250
1
0.5
0
[k_y=1..250] Nearest Neighbors
[k_x=1..250] Nearest Neighbors

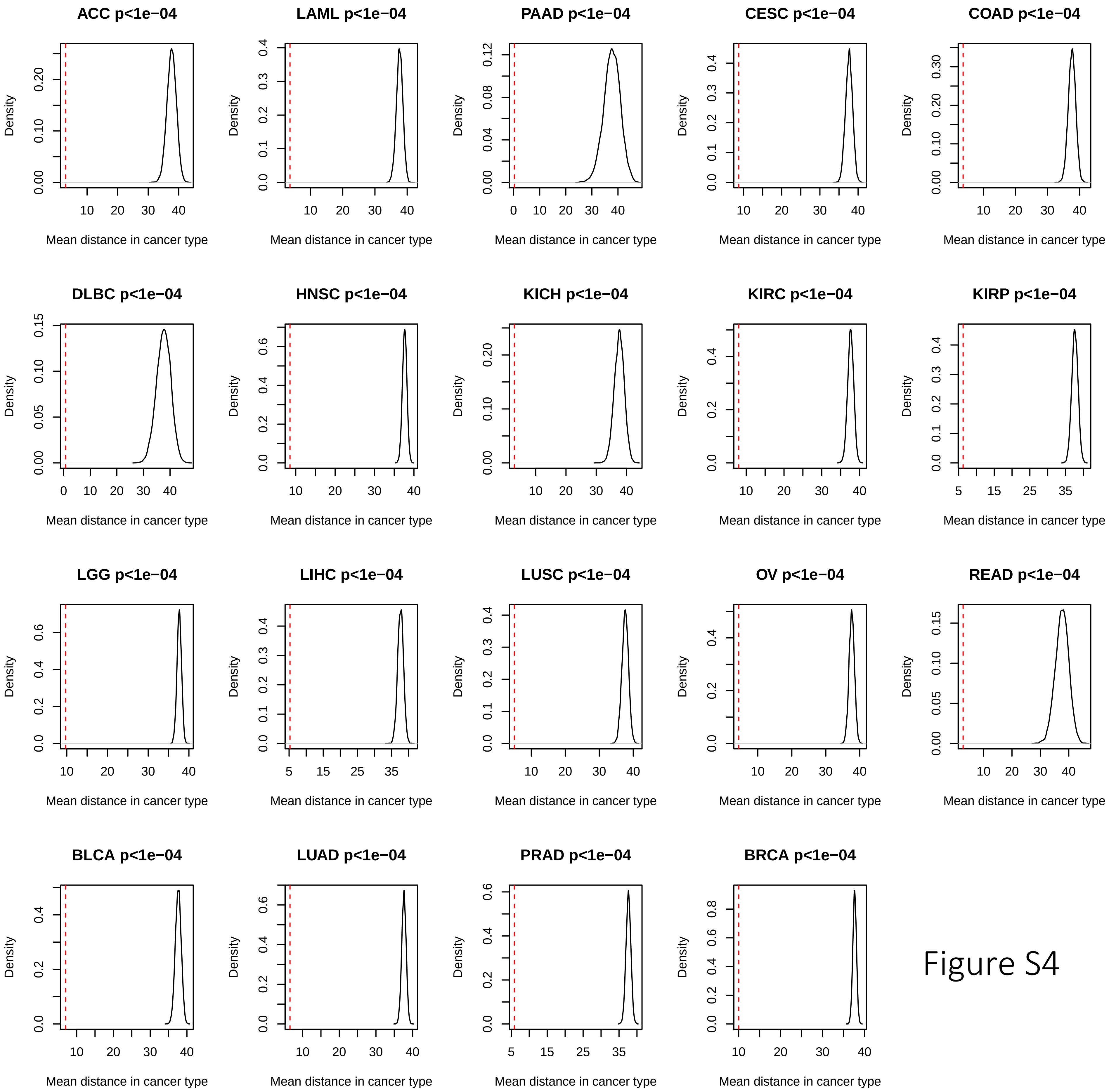
ACC p<1e−04
LAML p<1e−04
PAAD p<1e−04
CESC p<1e−04
COAD p<1e−04
DLBC p<1e−04
HNSC p<1e−04
KICH p<1e−04
KIRC p<1e−04
KIRP p<1e−04
LGG p<1e−04
LIHC p<1e−04
LUSC p<1e−04
OV p<1e−04
READ p<1e−04
BLCA p<1e−04
LUAD p<1e−04
PRAD p<1e−04
BRCA p<1e−04
Density
Mean distance in cancer type

Figure S4

Figure S5

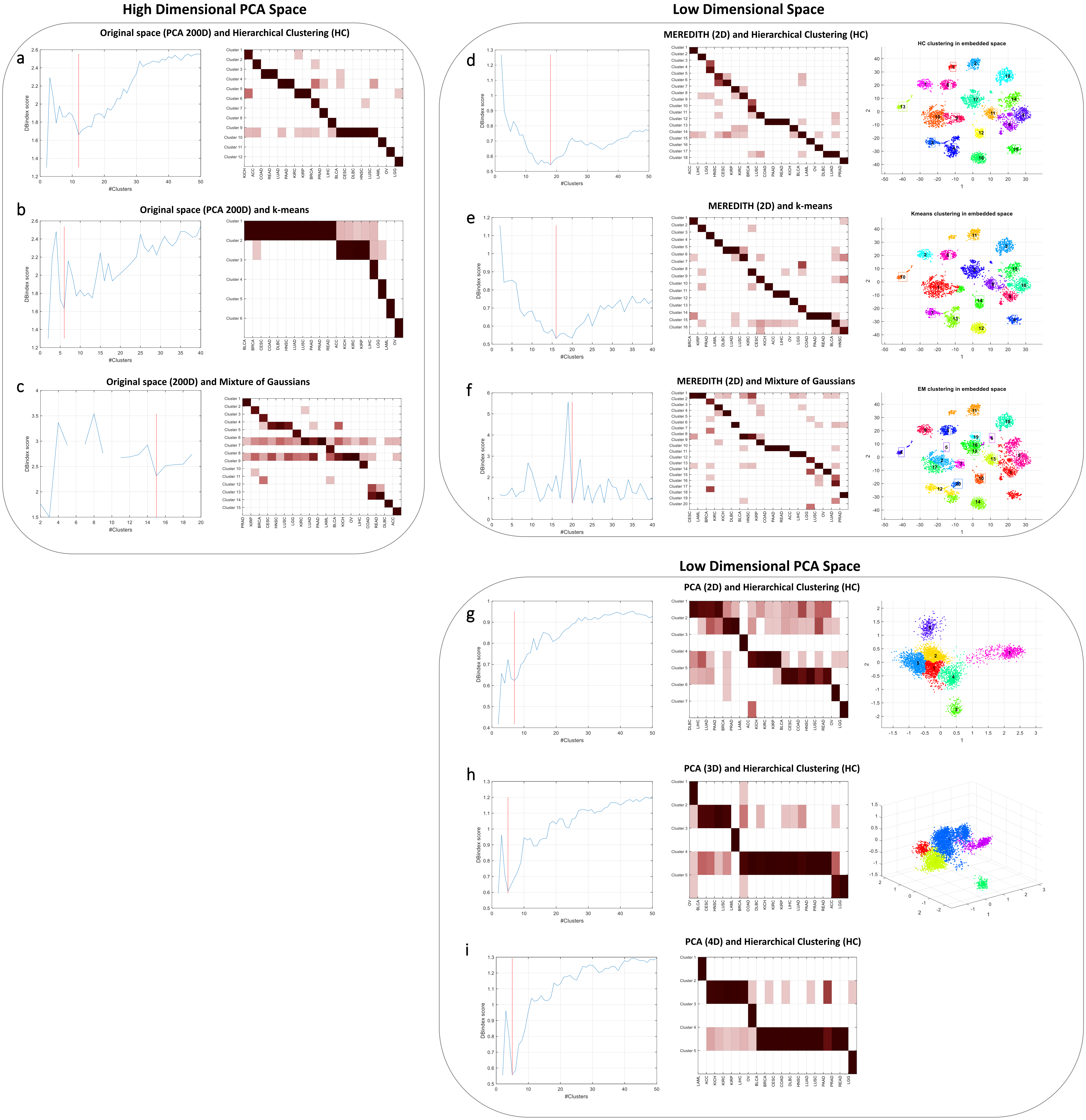
High Dimensional PCA Space
Original space (PCA 200D) and Hierarchical Clustering (HC)
a
Original space (PCA 200D) and k-means
b
Original space (200D) and Mixture of Gaussians
c
Low Dimensional Space
MEREDITH (2D) and Hierarchical Clustering (HC)
d
HC clustering in embedded space
MEREDITH (2D) and k-means
e
Kmeans clustering in embedded space
MEREDITH (2D) and Mixture of Gaussians
f
EM clustering in embedded space
Low Dimensional PCA Space
PCA (2D) and Hierarchical Clustering (HC)
g
PCA (3D) and Hierarchical Clustering (HC)
h
PCA (4D) and Hierarchical Clustering (HC)
i
DBindex score
#Clusters

Figure S6

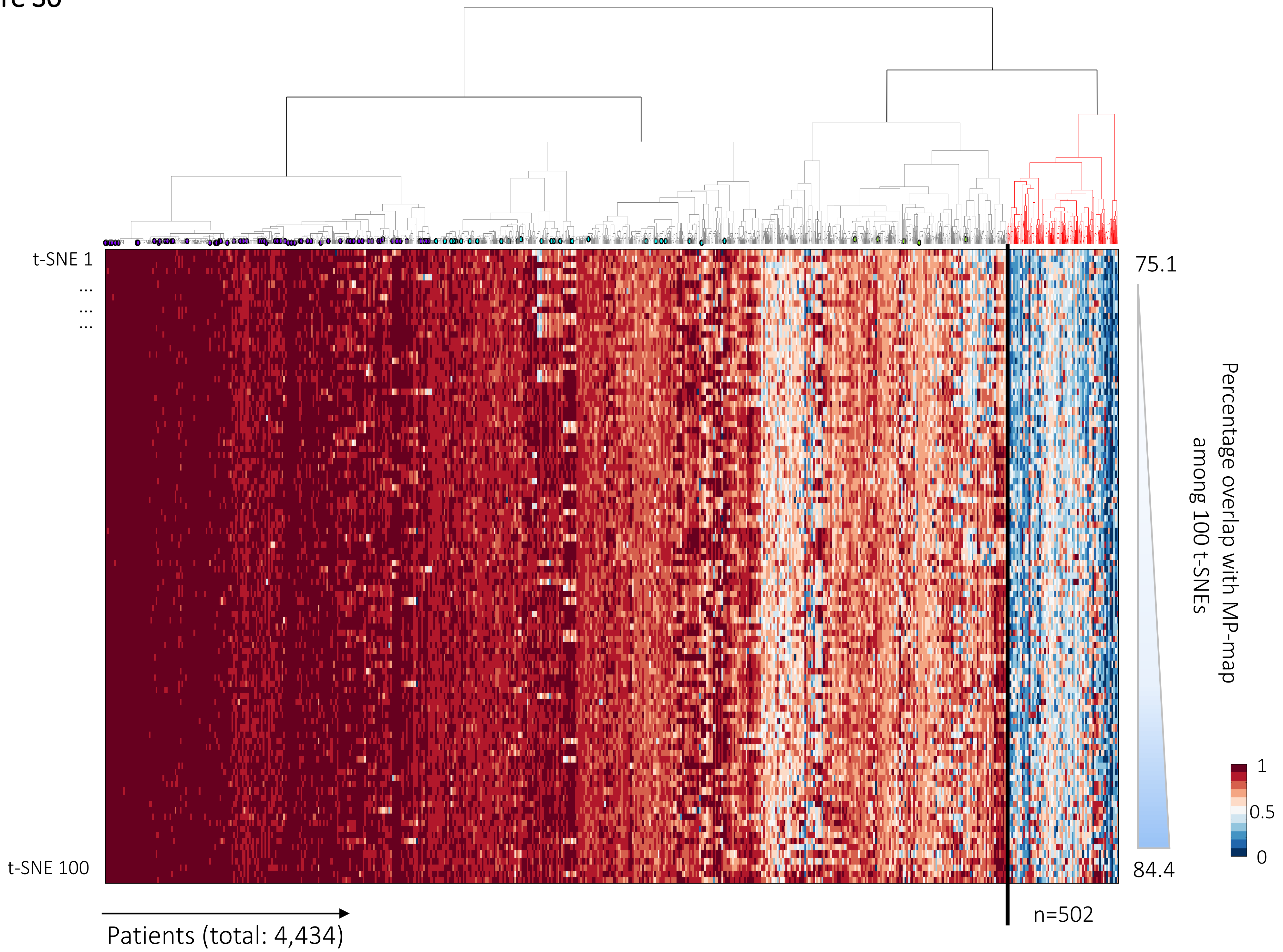
t-SNE 1
...
...
...
t-SNE 100
75.1
84.4
Percentage overlap with MP-map among 100 t-SNEs
1
0.5
0
n=502
Patients (total: 4,434)

Figure S7

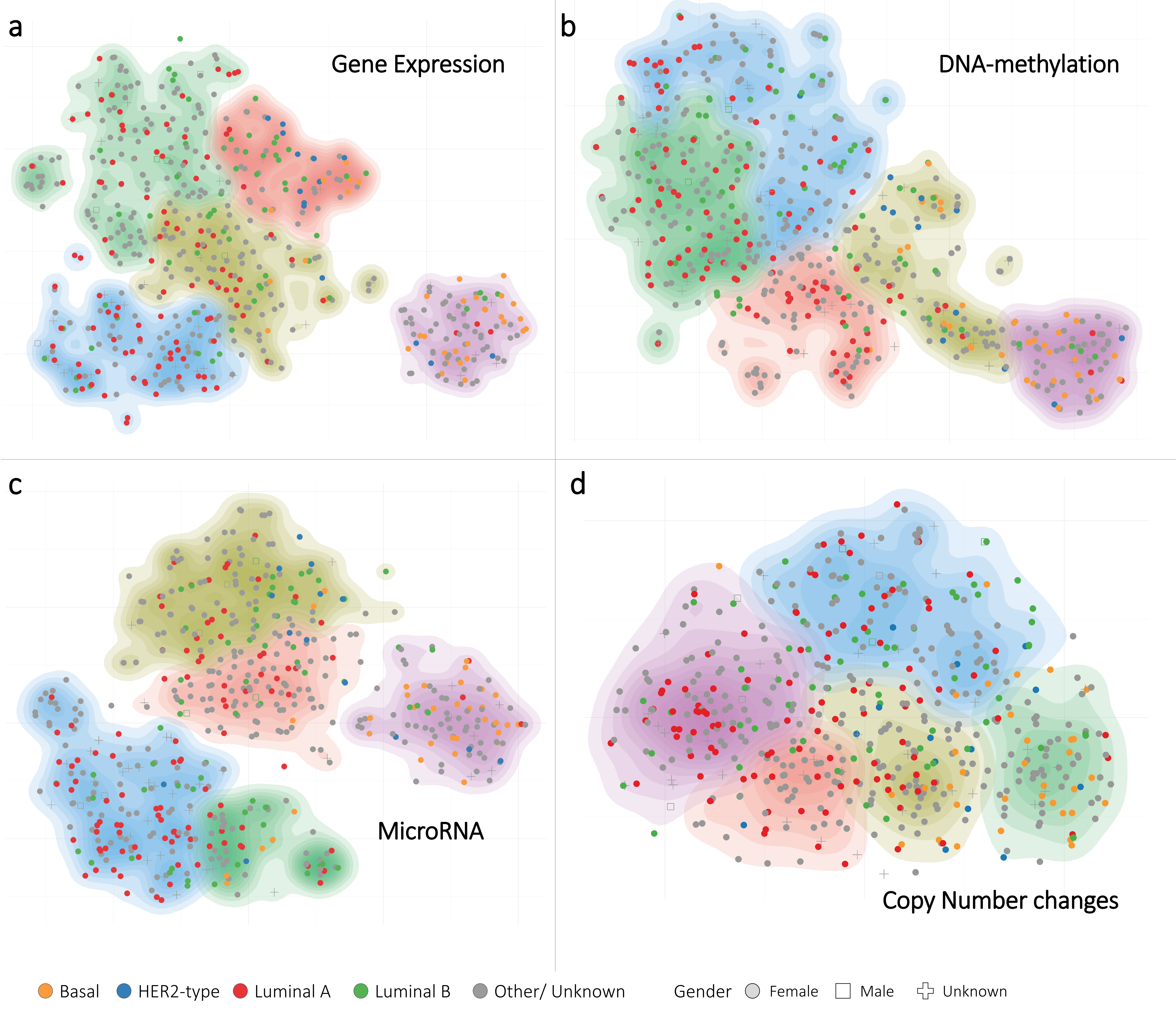
a
Gene Expression
b
DNA-methylation
c
MicroRNA
d
Copy Number changes
Basal
HER2-type
Luminal A
Luminal B
Other/ Unknown
Gender
Female
Male
Unknown

Figure S8

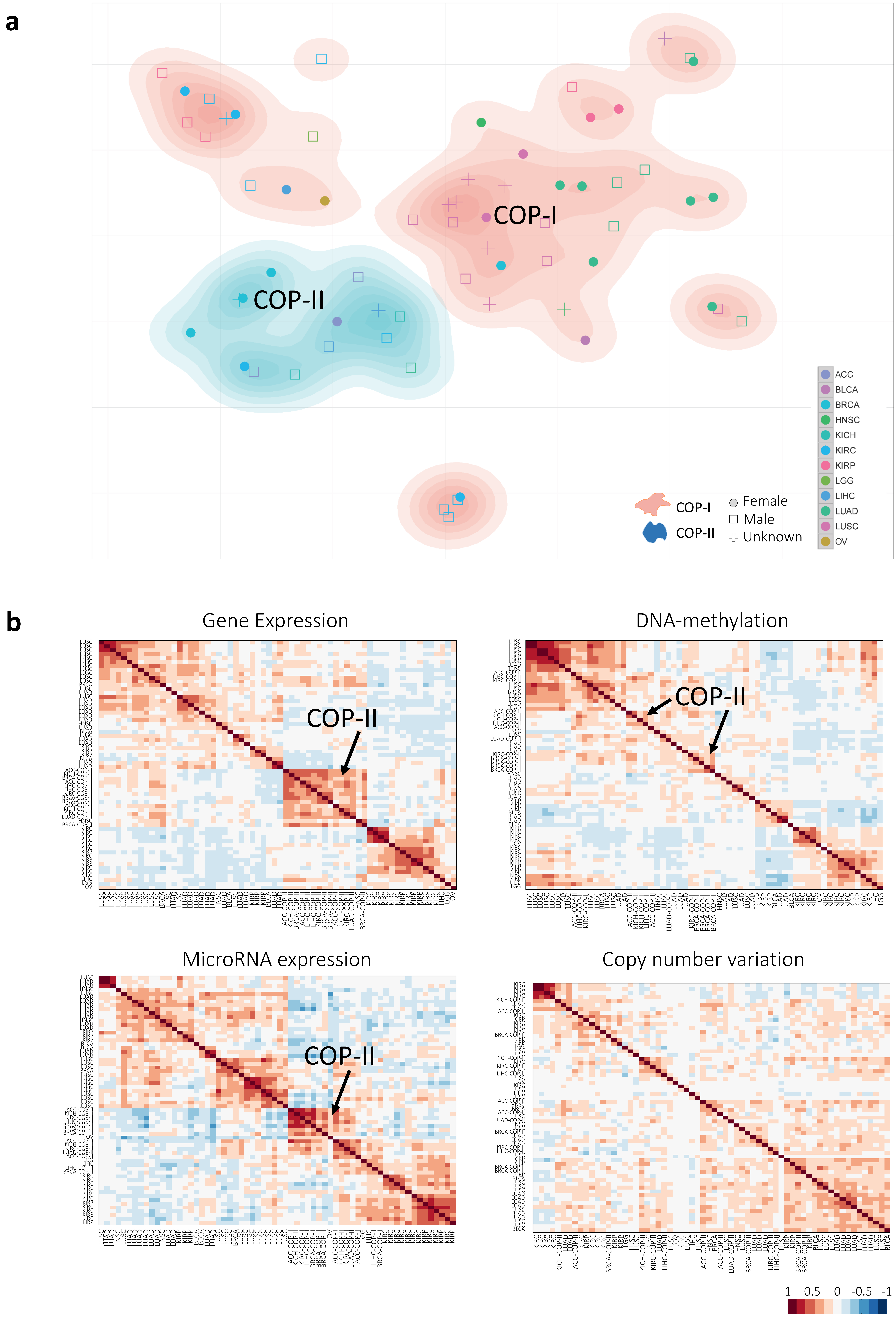
a
COP-I
COP-II
COP-I
COP-II
Female
Male
Unknown
ACC
BLCA
BRCA
HNSC
KICH
KIRC
KIRP
LGG
LIHC
LUAD
LUSC
OV
b
Gene Expression
COP-II
DNA-methylation
COP-II
MicroRNA expression
COP-II
Copy number variation
1
0.5
0
-0.5
-1

Figure S9

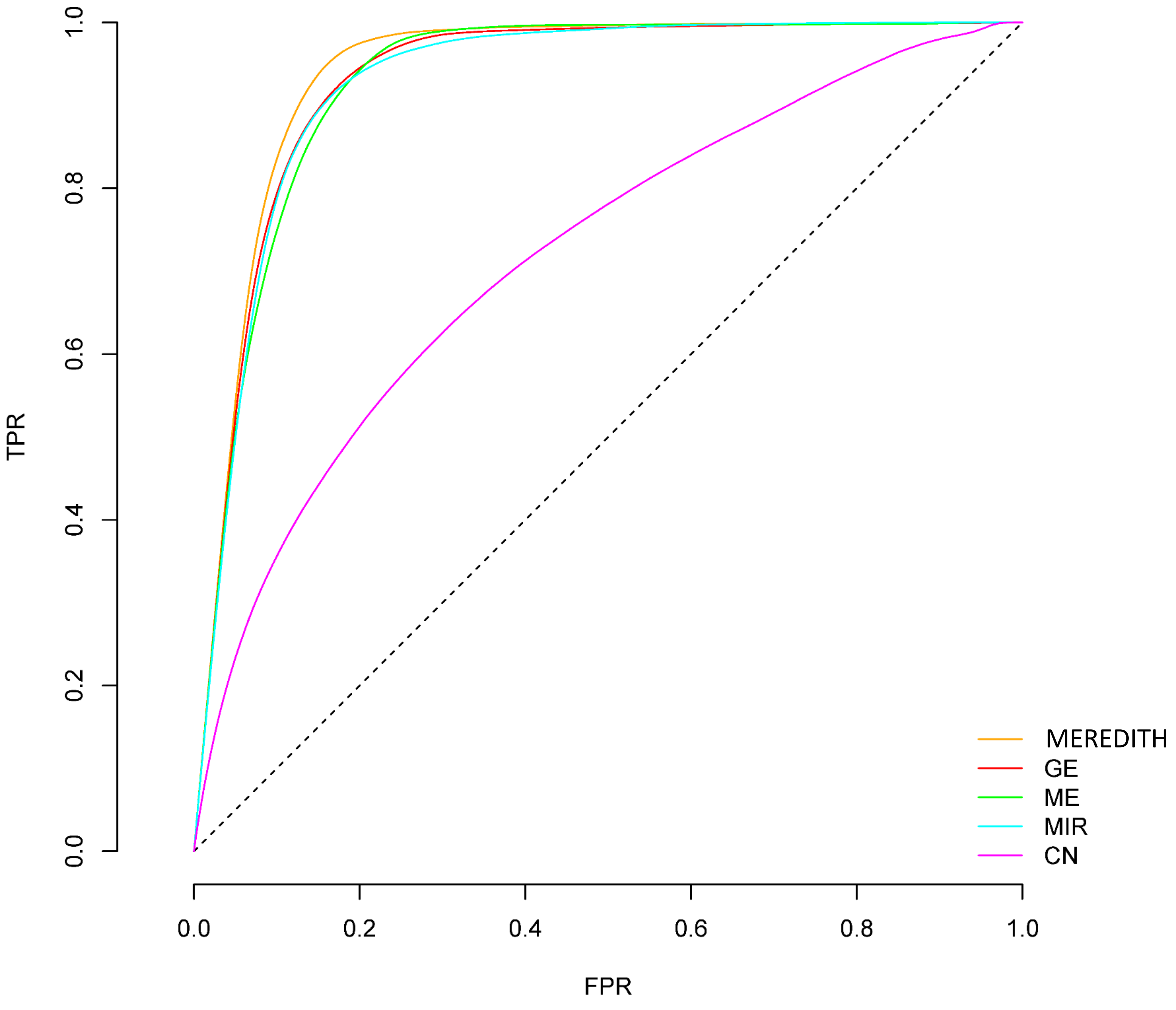

Figure S10

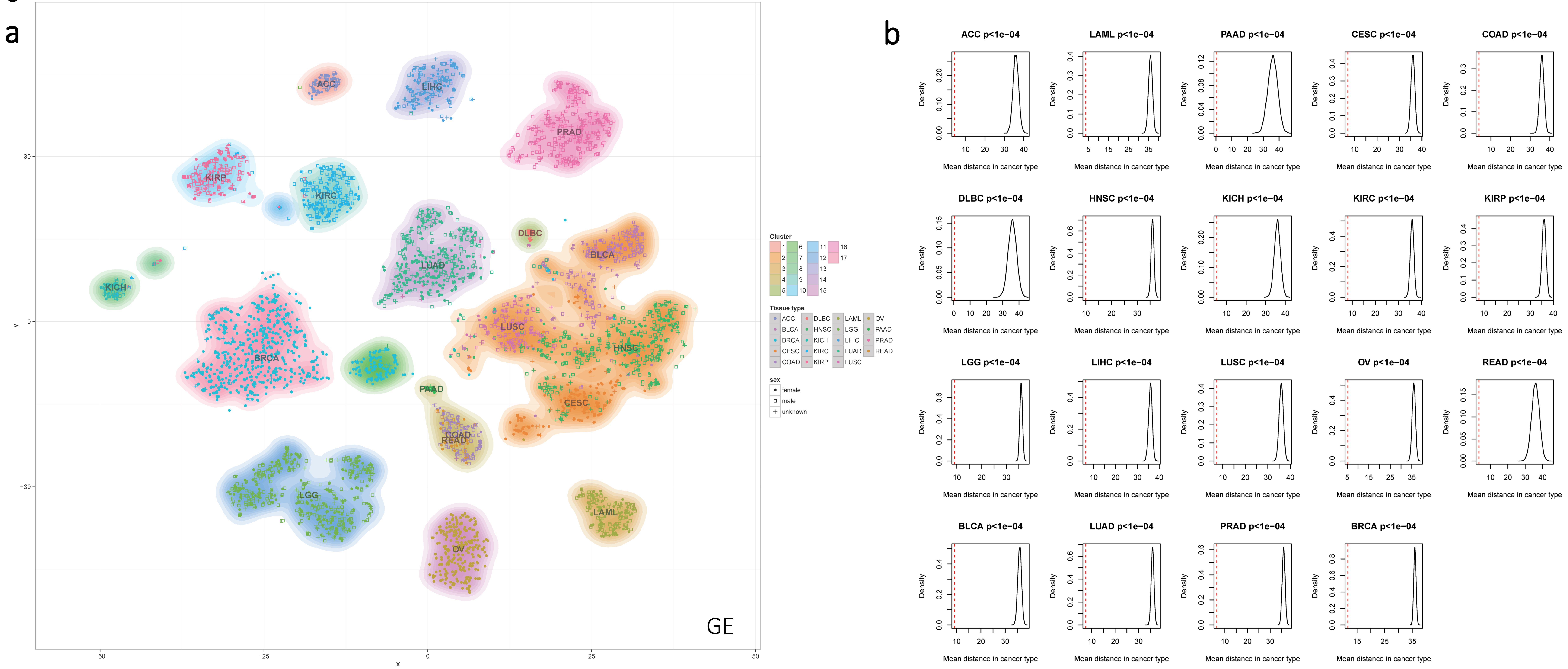
a
ACC
LIHC
PRAD
KIRP
KIRC
DLBC
BLCA
LUAD
KICH
LUSC
BRCA
HNSC
PAAD
CESC
COAD
READ
LGG
LAML
OV
GE
Cluster
Tissue type
sex
female
male
unknown
b
ACC p<1e−04
LAML p<1e−04
PAAD p<1e−04
CESC p<1e−04
COAD p<1e−04
DLBC p<1e−04
HNSC p<1e−04
KICH p<1e−04
KIRC p<1e−04
KIRP p<1e−04
LGG p<1e−04
LIHC p<1e−04
LUSC p<1e−04
OV p<1e−04
READ p<1e−04
BLCA p<1e−04
LUAD p<1e−04
PRAD p<1e−04
BRCA p<1e−04
Density
Mean distance in cancer type

Figure S11

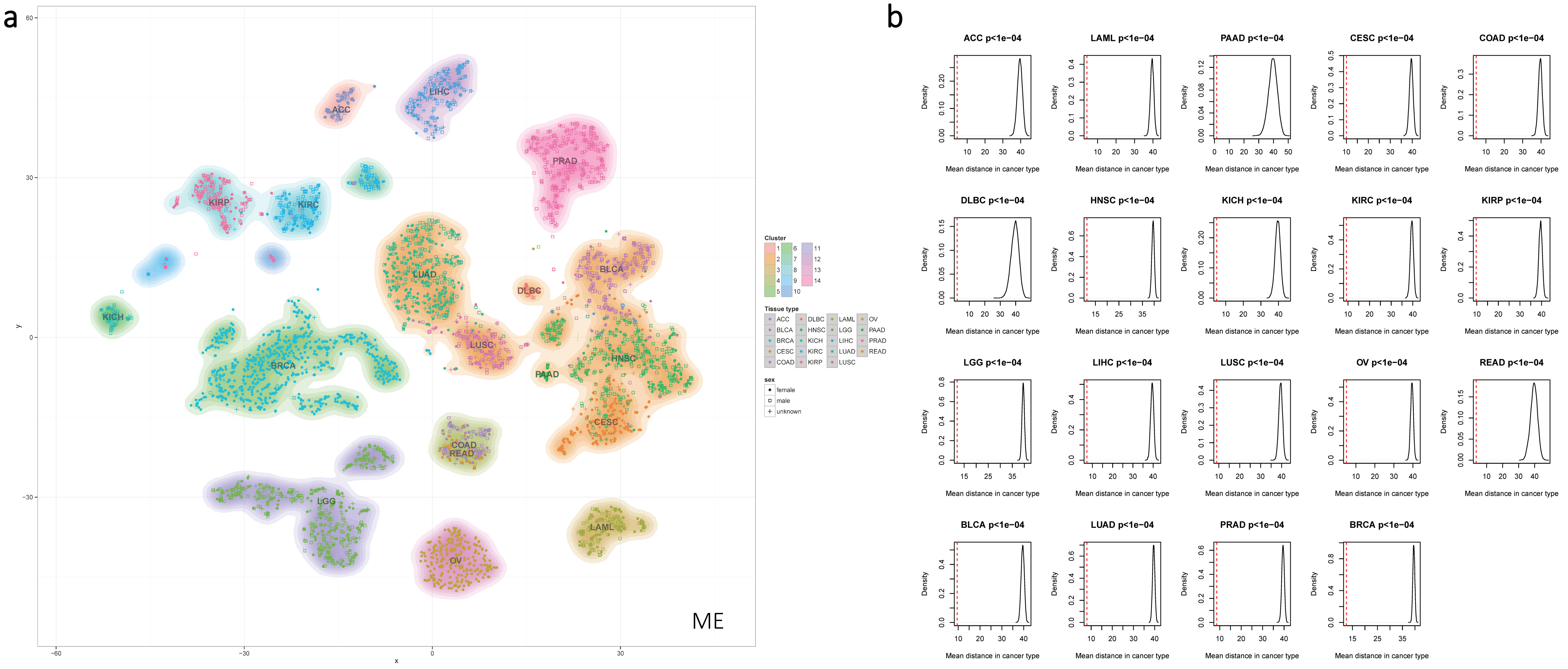
a
ACC
LIHC
PRAD
KIRP
KIRC
LUAD
BLCA
DLBC
KICH
BRCA
LUSC
HNSC
PAAD
CESC
COAD
READ
LGG
LAML
OV
ME
Cluster
Tissue type
sex
female
male
unknown
x
y
b
ACC p<1e−04
LAML p<1e−04
PAAD p<1e−04
CESC p<1e−04
COAD p<1e−04
DLBC p<1e−04
HNSC p<1e−04
KICH p<1e−04
KIRC p<1e−04
KIRP p<1e−04
LGG p<1e−04
LIHC p<1e−04
LUSC p<1e−04
OV p<1e−04
READ p<1e−04
BLCA p<1e−04
LUAD p<1e−04
PRAD p<1e−04
BRCA p<1e−04
Density
Mean distance in cancer type

Figure S12

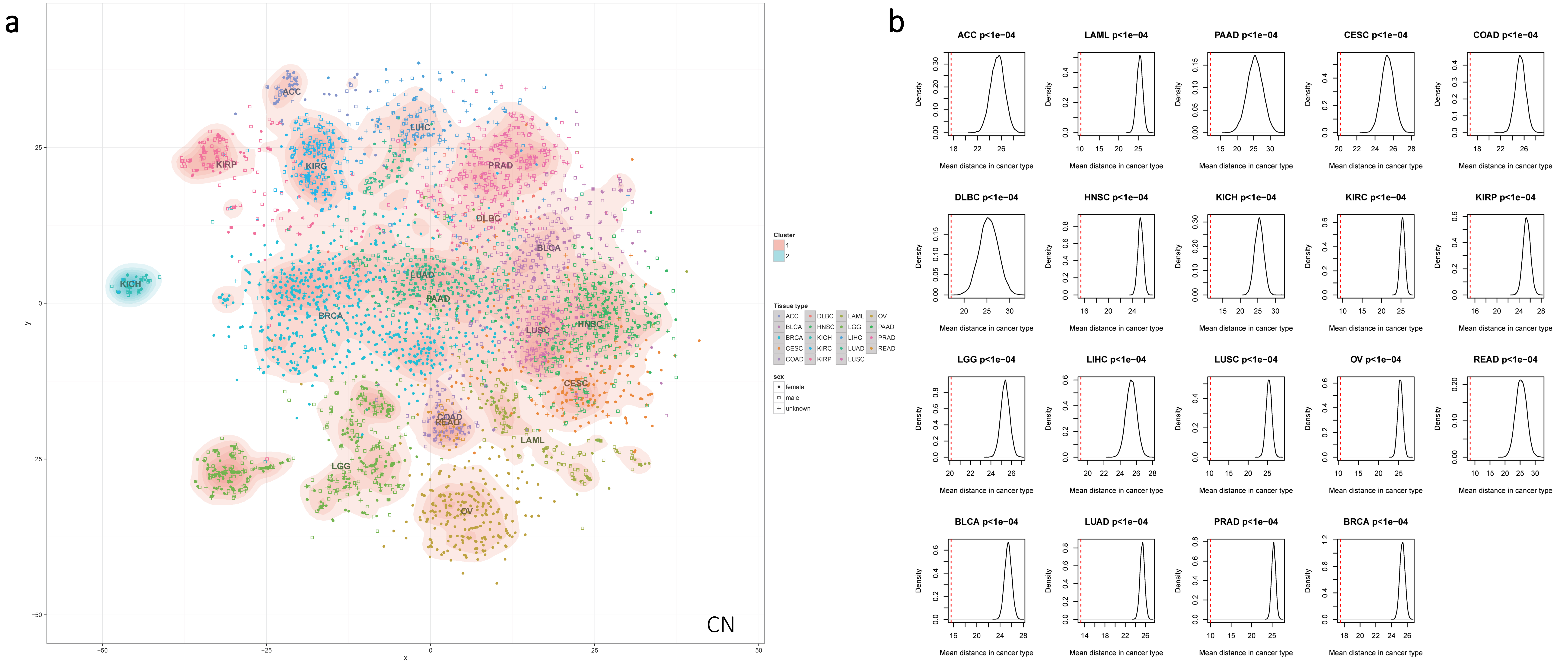
a
ACC
LIHC
KIRP
KIRC
PRAD
DLBC
BLCA
KICH
LUAD
PAAD
BRCA
LUSC
HNSC
CESC
COAD
READ
LAML
LGG
OV
CN
x
y
Cluster
1
2
Tissue type
ACC
BLCA
BRCA
CESC
COAD
DLBC
HNSC
KICH
KIRC
KIRP
LAML
LGG
LIHC
LUAD
LUSC
OV
PAAD
PRAD
READ
sex
female
male
unknown
b
ACC p<1e−04
LAML p<1e−04
PAAD p<1e−04
CESC p<1e−04
COAD p<1e−04
DLBC p<1e−04
HNSC p<1e−04
KICH p<1e−04
KIRC p<1e−04
KIRP p<1e−04
LGG p<1e−04
LIHC p<1e−04
LUSC p<1e−04
OV p<1e−04
READ p<1e−04
BLCA p<1e−04
LUAD p<1e−04
PRAD p<1e−04
BRCA p<1e−04
Density
Mean distance in cancer type

Figure S13

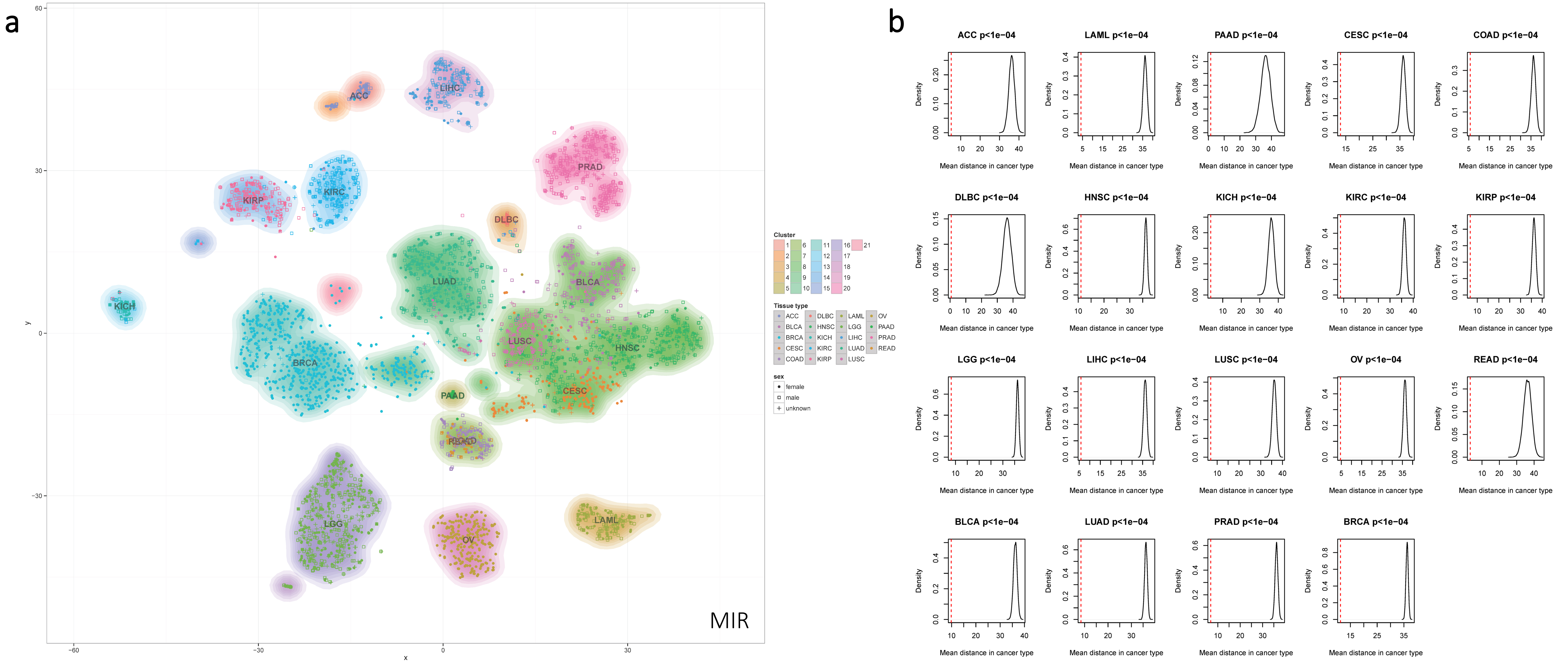
a
b
LIHC
ACC
PRAD
KIRP
KIRC
DLBC
KICH
LUAD
BLCA
LUSC
HNSC
BRCA
PAAD
CESC
READ
LGG
OV
LAML
MIR
x
y
Cluster
Tissue type
ACC
BLCA
BRCA
CESC
COAD
DLBC
HNSC
KICH
KIRC
KIRP
LAML
LGG
LIHC
LUAD
LUSC
OV
PAAD
PRAD
READ
sex
female
male
unknown
ACC p<1e−04
LAML p<1e−04
PAAD p<1e−04
CESC p<1e−04
COAD p<1e−04
DLBC p<1e−04
HNSC p<1e−04
KICH p<1e−04
KIRC p<1e−04
KIRP p<1e−04
LGG p<1e−04
LIHC p<1e−04
LUSC p<1e−04
OV p<1e−04
READ p<1e−04
BLCA p<1e−04
LUAD p<1e−04
PRAD p<1e−04
BRCA p<1e−04
Density
Mean distance in cancer type

Figure S14

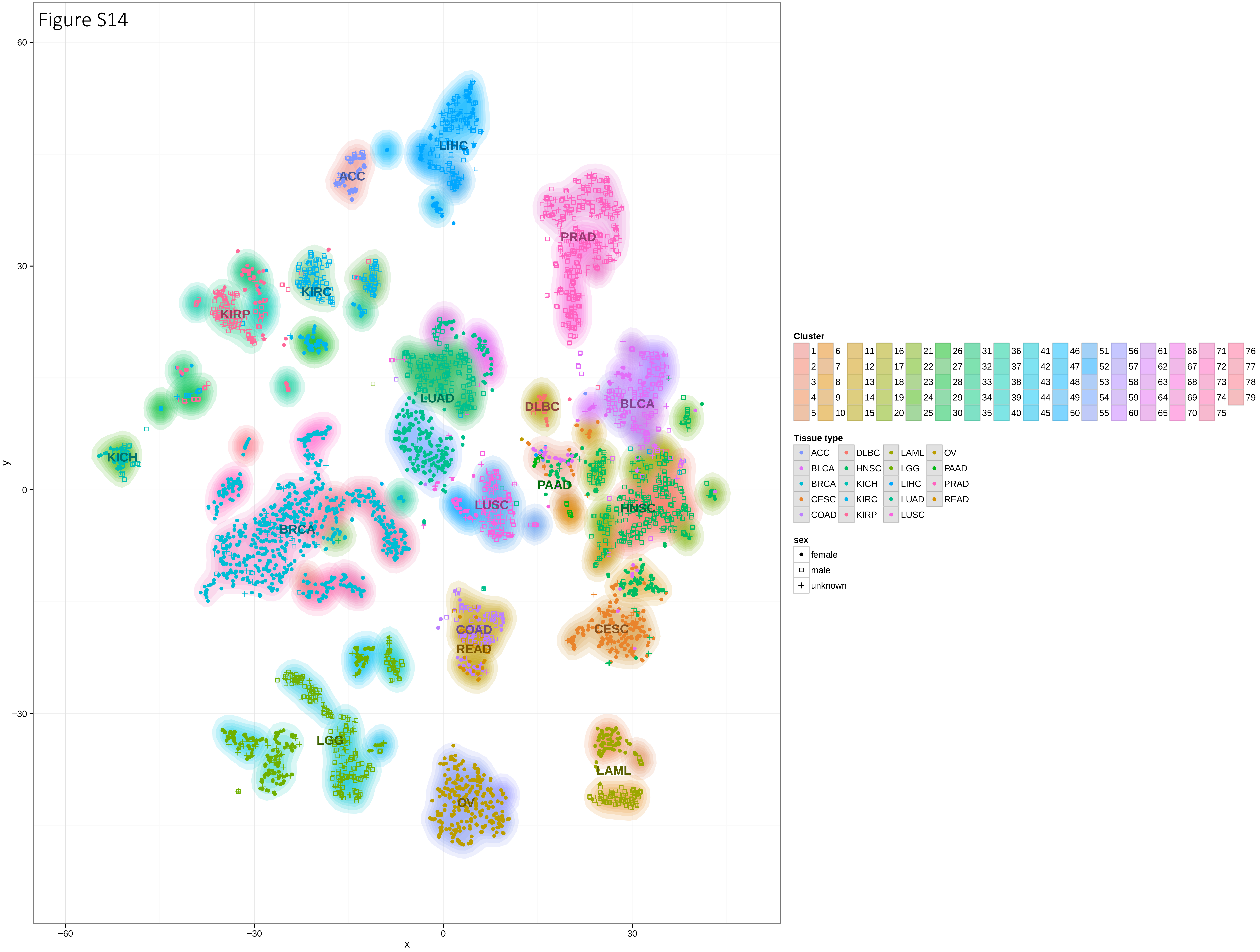

LIHC
ACC
PRAD
KIRC
KIRP
LUAD
DLBC
BLCA
KICH
PAAD
LUSC
HNSC
BRCA
COAD
READ
CESC
LGG
LAML
OV
60
30
0
−30
−60
y
x
Cluster
1 2 3 4 5 6 7 8 9 10 11 12 13 14 15 16 17 18 19 20 21 22 23 24 25 26 27 28 29 30 31 32 33 34 35 36 37 38 39 40 41 42 43 44 45 46 47 48 49 50 51 52 53 54 55 56 57 58 59 60 61 62 63 64 65 66 67 68 69 70 71 72 73 74 75 76 77 78 79
Tissue type
ACC
BLCA
BRCA
CESC
COAD
DLBC
HNSC
KICH
KIRC
KIRP
LAML
LGG
LIHC
LUAD
LUSC
OV
PAAD
PRAD
READ
sex
female
male
unknown

Figure S15

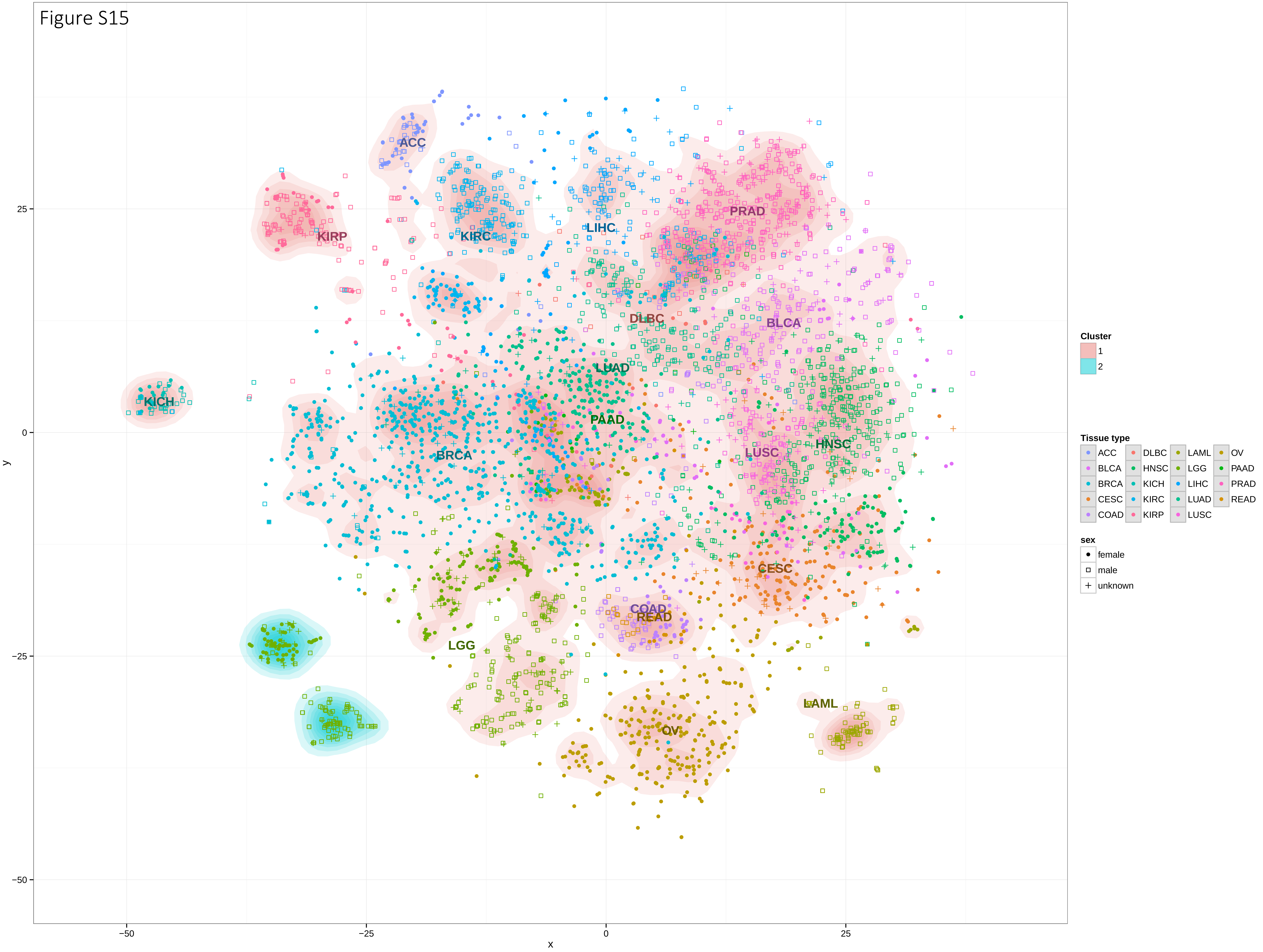

ACC
KIRP
KIRC
LIHC
PRAD
DLBC
BLCA
LUAD
KICH
PAAD
BRCA
LUSC
HNSC
CESC
COAD
READ
LGG
OV
LAML
25
0
−25
−50
y
x
−50
−25
0
25
Cluster
1
2
Tissue type
ACC
BLCA
BRCA
CESC
COAD
DLBC
HNSC
KICH
KIRC
KIRP
LAML
LGG
LIHC
LUAD
LUSC
OV
PAAD
PRAD
READ
sex
female
male
unknown

Figure S16

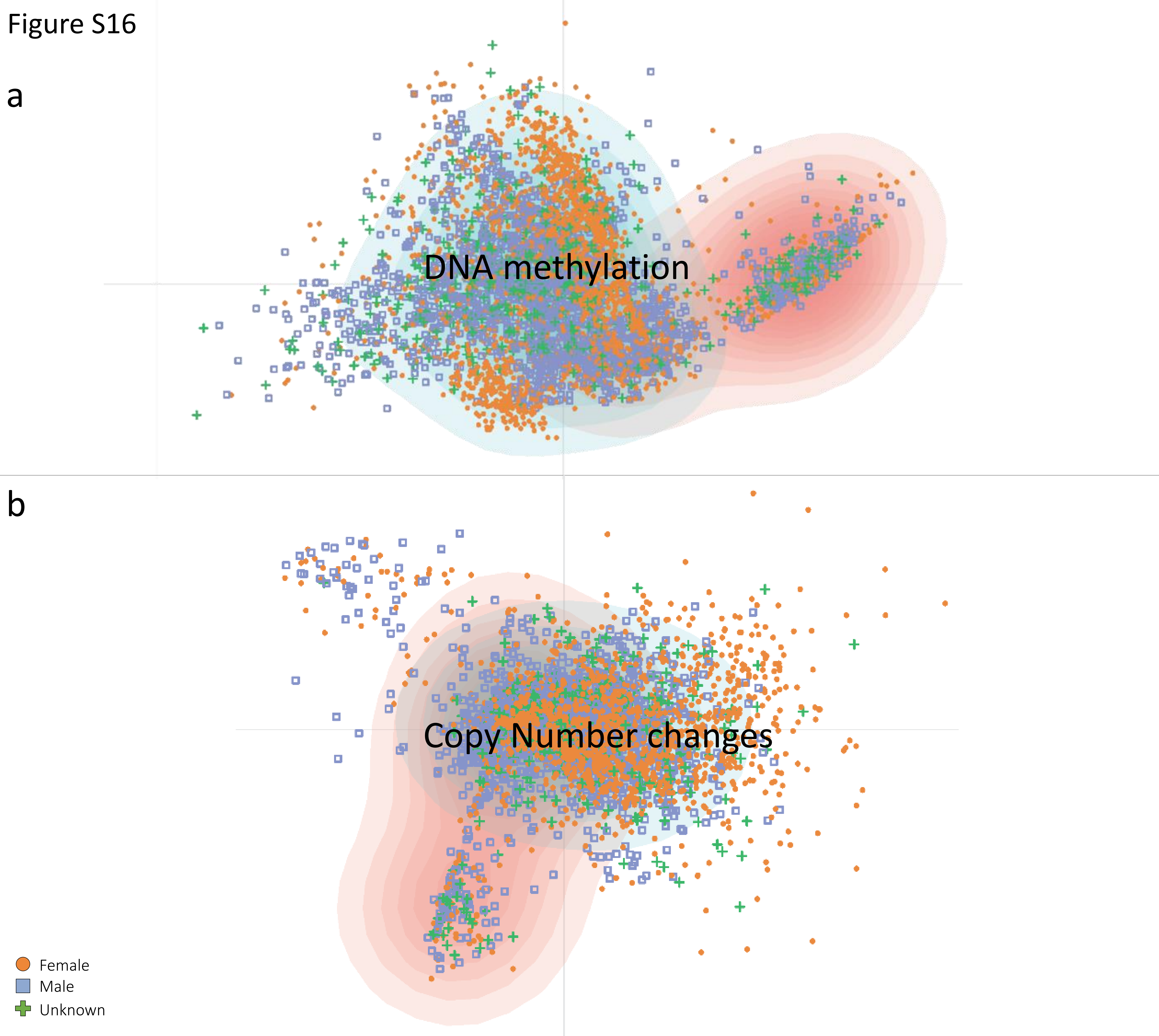
a
DNA methylation
b
Copy Number changes
Female
Male
Unknown

Figure S17

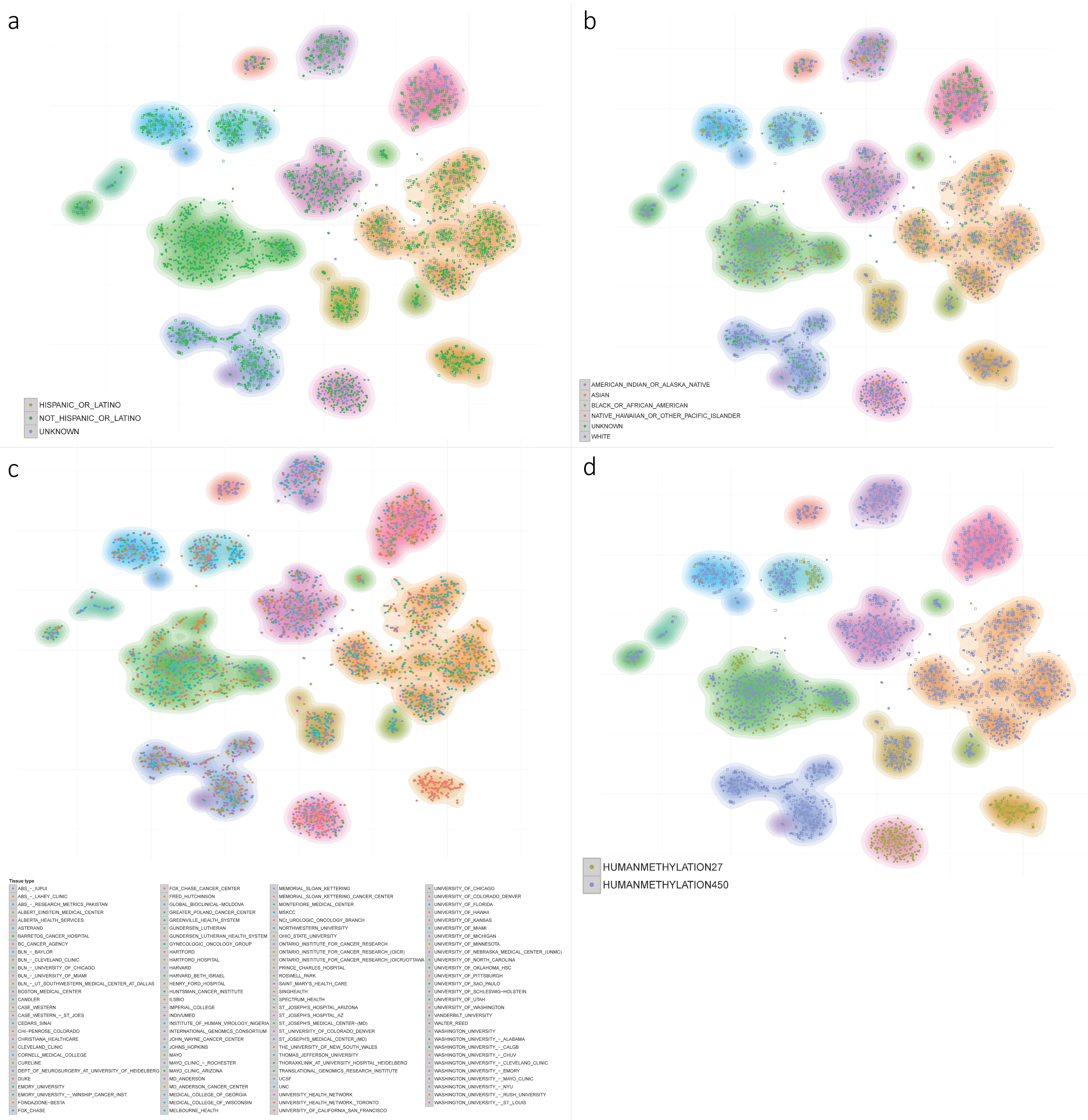
a
HISPANIC_OR_LATINO
NOT_HISPANIC_OR_LATINO
UNKNOWN
b
AMERICAN_INDIAN_OR_ALASKA_NATIVE
ASIAN
BLACK_OR_AFRICAN_AMERICAN
NATIVE_HAWAIIAN_OR_OTHER_PACIFIC_ISLANDER
UNKNOWN
WHITE
c
Tissue type
ABS_-_IUPUI
ABS_-_LAHEY_CLINIC
ABS_-_RESEARCH_METRICS_PAKISTAN
ALBERT_EINSTEIN_MEDICAL_CENTER
ALBERTA_HEALTH_SERVICES
ASTERAND
BARRETOS_CANCER_HOSPITAL
BC_CANCER_AGENCY
BLN_-_BAYLOR
BLN_-_CLEVELAND_CLINIC
BLN_-_UNIVERSITY_OF_CHICAGO
BLN_-_UNIVERSITY_OF_MIAMI
BLN_-_UT_SOUTHWESTERN_MEDICAL_CENTER_AT_DALLAS
BOSTON_MEDICAL_CENTER
CANDLER
CASE_WESTERN
CASE_WESTERN_-_ST_JOES
CEDARS_SINAI
CHI-PENROSE_COLORADO
CHRISTIANA_HEALTHCARE
CLEVELAND_CLINIC
CORNELL_MEDICAL_COLLEGE
CURELINE
DEPT_OF_NEUROSURGERY_AT_UNIVERSITY_OF_HEIDELBERG
DUKE
EMORY_UNIVERSITY
EMORY_UNIVERSITY_-_WINSHIP_CANCER_INST.
FONDAZIONE-BESTA
FOX_CHASE
FOX_CHASE_CANCER_CENTER
FRED_HUTCHINSON
GLOBAL_BIOCLINICAL-MOLDOVA
GREATER_POLAND_CANCER_CENTER
GREENVILLE_HEALTH_SYSTEM
GUNDERSEN_LUTHERAN
GUNDERSEN_LUTHERAN_HEALTH_SYSTEM
GYNECOLOGIC_ONCOLOGY_GROUP
HARTFORD
HARTFORD_HOSPITAL
HARVARD
HARVARD_BETH_ISRAEL
HENRY_FORD_HOSPITAL
HUNTSMAN_CANCER_INSTITUTE
ILSBIO
IMPERIAL_COLLEGE
INDIVUMED
INSTITUTE_OF_HUMAN_VIROLOGY_NIGERIA
INTERNATIONAL_GENOMICS_CONSORTIUM
JOHN_WAYNE_CANCER_CENTER
JOHNS_HOPKINS
MAYO
MAYO_CLINIC_-_ROCHESTER
MAYO_CLINIC_ARIZONA
MD_ANDERSON
MD_ANDERSON_CANCER_CENTER
MEDICAL_COLLEGE_OF_GEORGIA
MEDICAL_COLLEGE_OF_WISCONSIN
MELBOURNE_HEALTH
MEMORIAL_SLOAN_KETTERING
MEMORIAL_SLOAN_KETTERING_CANCER_CENTER
MONTEFIORE_MEDICAL_CENTER
MSKCC
NCI_UROLOGIC_ONCOLOGY_BRANCH
NORTHWESTERN_UNIVERSITY
OHIO_STATE_UNIVERSITY
ONTARIO_INSTITUTE_FOR_CANCER_RESEARCH
ONTARIO_INSTITUTE_FOR_CANCER_RESEARCH_(OICR)
ONTARIO_INSTITUTE_FOR_CANCER_RESEARCH_(OICR)/OTTAWA
PRINCE_CHARLES_HOSPITAL
ROSWELL_PARK
SAINT_MARY'S_HEALTH_CARE
SINGHEALTH
SPECTRUM_HEALTH
ST._JOSEPH'S_HOSPITAL_ARIZONA
ST._JOSEPH'S_HOSPITAL_AZ
ST._JOSEPH'S_MEDICAL_CENTER-(MD)
ST._UNIVERSITY_OF_COLORADO_DENVER
ST_JOSEPH'S_MEDICAL_CENTER_(MD)
THE_UNIVERSITY_OF_NEW_SOUTH_WALES
THOMAS_JEFFERSON_UNIVERSITY
THORAXKLINIK_AT_UNIVERSITY_HOSPITAL_HEIDELBERG
TRANSLATIONAL_GENOMICS_RESEARCH_INSTITUTE
UCSF
UNC
UNIVERSITY_HEALTH_NETWORK
UNIVERSITY_HEALTH_NETWORK,_TORONTO
UNIVERSITY_OF_CALIFORNIA_SAN_FRANCISCO
UNIVERSITY_OF_CHICAGO
UNIVERSITY_OF_COLORADO_DENVER
UNIVERSITY_OF_FLORIDA
UNIVERSITY_OF_HAWAII
UNIVERSITY_OF_KANSAS
UNIVERSITY_OF_MIAMI
UNIVERSITY_OF_MICHIGAN
UNIVERSITY_OF_MINNESOTA
UNIVERSITY_OF_NEBRASKA_MEDICAL_CENTER_(UNMC)
UNIVERSITY_OF_NORTH_CAROLINA
UNIVERSITY_OF_OKLAHOMA_HSC
UNIVERSITY_OF_PITTSBURGH
UNIVERSITY_OF_SAO_PAULO
UNIVERSITY_OF_SCHLESWIG-HOLSTEIN
UNIVERSITY_OF_UTAH
UNIVERSITY_OF_WASHINGTON
VANDERBILT_UNIVERSITY
WALTER_REED
WASHINGTON_UNIVERSITY
WASHINGTON_UNIVERSITY_-_ALABAMA
WASHINGTON_UNIVERSITY_-_CALGB
WASHINGTON_UNIVERSITY_-_CHUV
WASHINGTON_UNIVERSITY_-_CLEVELAND_CLINIC
WASHINGTON_UNIVERSITY_-_EMORY
WASHINGTON_UNIVERSITY_-_MAYO_CLINIC
WASHINGTON_UNIVERSITY_-_NYU
WASHINGTON_UNIVERSITY_-_RUSH_UNIVERSITY
WASHINGTON_UNIVERSITY_-_ST._LOUIS
d
HUMANMETHYLATION27
HUMANMETHYLATION450

Figure S18

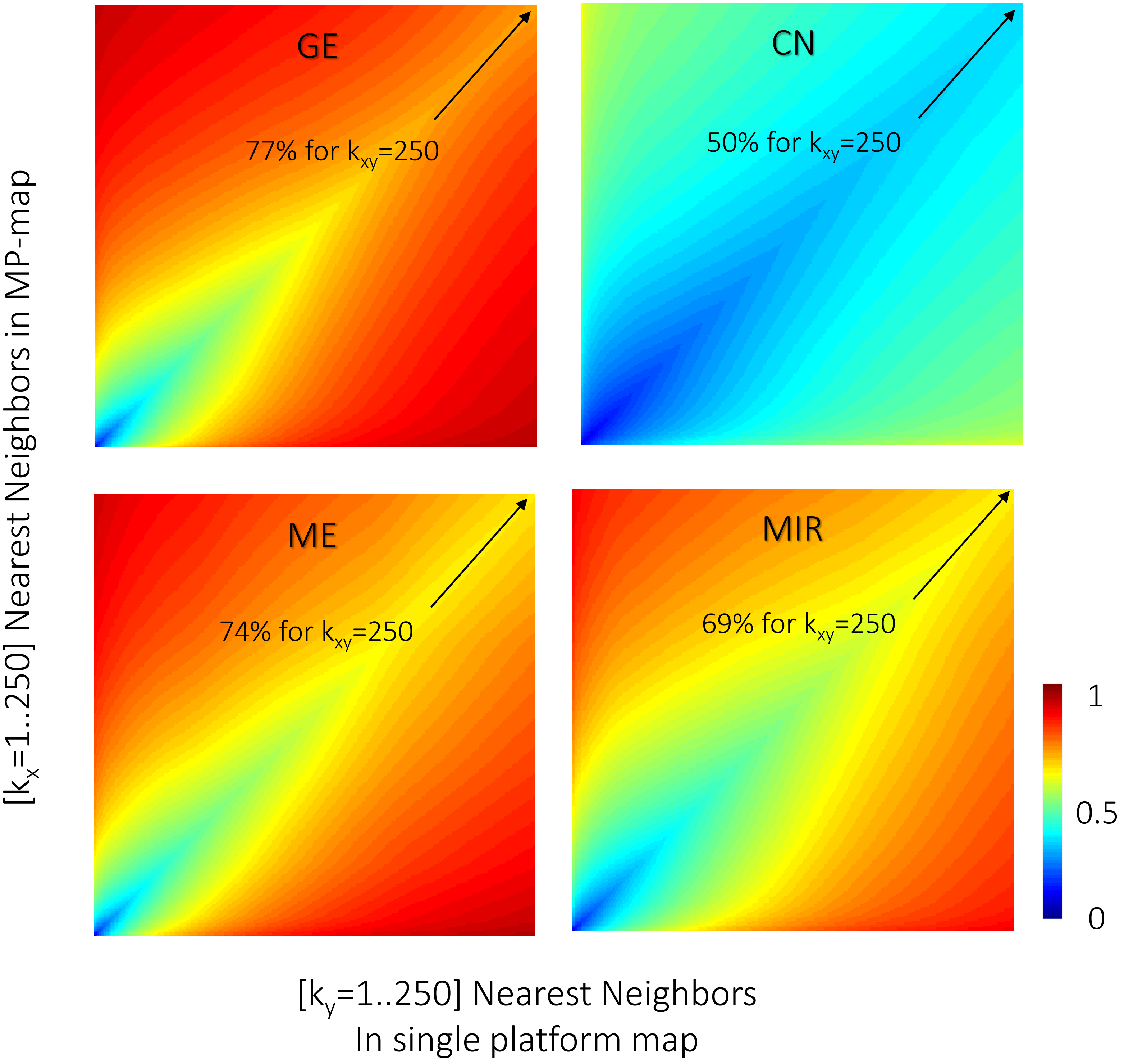
GE
77% for k_{xy}=250
CN
50% for k_{xy}=250
ME
74% for k_{xy}=250
MIR
69% for k_{xy}=250
1
0.5
0
[k_x=1..250] Nearest Neighbors in MP-map
[k_y=1..250] Nearest Neighbors
In single platform map

Table S1

Cancer tissues/Clustering algorithm	Low Dimensional Space								Low Dimensional PCA Space						High dimensional PCA Space					
	MEREDITH (2D) and DBSCAN		MEREDITH (2D) and HC		MEREDITH (2D) and k-means		MEREDITH (2D) and Mixture of Gaussians		PCA(2D) and HC		PCA(3D) and HC		PCA(4D) and HC		Original space (200D) and HC		Original space (200D) and k-means		Original space (200D) and Mixture of Gaussians	
	P Enrichment	Cl.label	P Enrichment	Cl.label	P Enrichment	Cl.label	P Enrichment	Cl.label	P Enrichment	Cl.label	P Enrichment	Cl.label	P Enrichment	Cl.label	P Enrichment	Cl.label	P Enrichment	Cl.label	P Enrichment	Cl.label
ACC	6.122E-158	1	6.122E-158	1	2.195E-91	11	2.195E-91	11	2.352E-31	4	9.356E-40	5	3.659E-50	2	4.589E-156	2	1.559E-63	2	5.82E-152	15
BLCA	2.604E-162	2	9.468e-314	14	3.443E-307	15	9.844E-200	8	6.048E-102	5	4.803E-80	2	1.97E-54	4	1.412E-153	9	2.621E-54	1	0.001807,3.667e-31	4,8
BRCA	0	7	6.438e-179,0	9,10	0,2.41e-09	1,14	2.712e-321,2.186e-91,6.911e-203	2,7,17	3.216E-308	2	6.634E-84	4	6.982E-160	4	0.00271,0	4,6	1.677E-159	1	0,3.14e-88	3,11
CESC	1.116e-65,5.638e-59	2,5	4.589e-178,0.001676	6,14	1.609E-212	9	1.413E-158	1	4.76E-90	5	9.279E-70	2	8.389E-44	4	4.025E-127	9	1.054E-43	1	6.739e-61	4
COAD	5.488E-201	4	5.726E-200	12	7.12E-171	14	5.726E-200	10	4.309e-05,2.706e-24	1,5	5.75E-37	4	2.229E-25	4	1.408E-212	3	3.143E-27	1	6.251e-61,1.771e-112	12,13
DLBC	4.055E-71	6	6.843E-28	17	6.843E-28	5	3.958E-58	6	3.649E-15	1	5.916E-07	4	0.000002456	4	7.032E-17	9	0.000002529	1	1.79E-67	14
HNSC	2.901e-318	2	6.718e-296,1.408e-76	5,6	4.901e-09,2.179e-13,0	6,9,16	9.943e-40,1.48e-179	1,8	2.531e-240	5	8.218e-279	2	1.848E-101	4	3.682E-303	9	3.171E-101	1	9.95E-248	4
KICH	2.295e-110,8.737e-12	8,9	2.207E-124	13	2.207E-124	10	2.207E-124	4	5.66e-46	4	1.592E-19	4	1.626E-47	2	2.446E-105	1	1.162E-55	2	1.56E-31	8
KIRC	0.0007566,0	9,10	0,0.009809	8,13	0,0.009809	8,10	0,0.009809	3,4	5.55E-206	4	1.464E-69	4	2.434E-189	2	6.523E-250	5	2.828E-224	2	6.01E-120	6
KIRP	3.066e-05,3.912e-281,5.919e-13	9,11,12	2.102E-302	7	2.102E-302	2	2.102E-302	9	5.272E-171	4	2.977E-52	4	1.551E-151	2	9.458e-212	5	2.118E-179	2	3.377e-205,1.691e-07	2,10
LAML	4.77E-295	3	3.445E-308	15	3.445E-308	4	4.735E-165	1	3.445e-308	3	3.445E-308	3	3.445E-308	1	3.445e-308	10	3.445E-308	5	8.84E-207	7
LGG	0,1.864e-05	13,14	8.158e-162,0	3,4	4.253e-165,0	7,13	3.02e-301,1.033e-43,7.448e-80	12,14,20	0	7	0	5	0	5	0	12	0	4	0	5
LIHC	0	15	0	2	7.614E-266	11	7.614E-266	11	5.13e-67,0.0001416	1,4	2.828E-54	4	2.75E-137	2	0	8	1.113e-307	3	2.08E-270	9
LUAD	0	16	0	17	0	5	2.91e-06,1.729e-87,1.155e-274,1.273e-53	6,15,16,19	1.217E-145	1	9.723E-69	4	1.096E-90	4	0	4	5.94E-93	1	6.829e-55,8.529e-34	6,8
LUSC	1.692E-91	2	6.644E-267	11	8.373E-222	6	2.488E-260	13	4.173E-14	5	1.241E-76	2	2.024E-39	4	5.801E-86	9	2.485E-39	1	4.48E-46	4
OV	0	17	0	16	0	12	1.49E-307	14	0	6	0	1	0	3	0	11	0	6	1.29E-168	8
PAAD	5.796E-29	4	7.31E-29	12	1.234E-25	14	7.31E-29	10	1.878E-11	1	0.000002216	4	-	-	6.579E-19	4	0.00007216	1	1.07E-15	6
PRAD	0	18	0	18	0	3	0	18	9.365E-182	2	1.002E-97	4	1.364E-71	4	0	7	1.99E-71	1	0	1
READ	2.44E-49	4	3.685E-49	12	1.793E-43	14	3.685E-49	10	1.122E-10	5	2.322E-10	4	8.484E-08	4	2.92E-51	3	8.806E-08	1	4.68E-51	13

Green color depicts (equal) highest cluster enrichment for cancer type

Table S2: Patient demographics and clinical characteristics of Acute Myeloid Leukemia patients in the 11 clusters

	Cl.1 (n=6)	P1	Cl.2 (n=20)	P2	Cl.3 (n=11)	P3	Cl.4 (n=18)	P4	Cl.5 (n=7)	P5	Cl.6 (n=35)	P6	Cl.7 (n=15)	P7	Cl.8 (n=15)	P8	Cl.9 (n=4)	P9	Cl.10 (n=21)	P10	Cl.11 (n=15)	P11
**Age, years**		0.36		0.94		0.61		**0.0003**		0.49		0.37		**0.00055**		1		0.4		0.29		0.067
median	53		58		56		37		61		62		71		63		65		64		51	
range	22-77		22-83		23-76		22-75		45-77		31-82		44-82		28-74		42-78		18-89		30-75	
**Sex, n (%)**		0.93		0.94		0.61		0.35		0.29		0.56		0.22		0.63		0.1		0.65		0.92
Male	2 (33%)		8 (40%)		6 (55%)		11 (61%)		5 (71%)		19 (54%)		10 (67%)		8 (53%)		4 (1e+02%)		11 (52%)		6 (40%)	
Female	4 (67%)		12 (60%)		5 (45%)		7 (39%)		2 (29%)		16 (46%)		5 (33%)		7 (47%)		0 (0%)		10 (48%)		9 (60%)	
**Blast percentage**		0.8		0.76		0.2		0.81		0.31		0.39		0.06		0.13		0.38		0.82		0.98
Median	0		0		0		0		0		0		0		0		0		0		0	
Range	0-1		0-10		0-0		0-1		0-0		0-8		0-6		0-0		0-6		0-2		0-1	
**Lymphocytes**		**0.0023**		**0.0002**		0.69		**0.04**		0.2		0.45		0.06		0.59		0.48		0.17		**0.00052**
Median	87		72		48		52		0		18		12		34		25		14		2	
Range	43-97		okt-98		0-70		0-96		0-87		0-90		0-83		0-97		feb-51		0-90		0-68	
**Monocyte**		0.2		0.26		0.073		0.42		0.18		0.78		0.34		0.33		0.8		0.87		0.6
Median	0		0		1		0		0		0		0		0		1		0		0	
Range	0-1		0-15		0-11		0-48		0-2		0-40		0-14		0-40		0-4		0-8		0-8	
**Neutrophil**		0.0091		0.2		0.48		0.31		0.64		0.72		0.061		0.47		0.85		0.089		0.15
Median	1		4		5		5		5		7		14		4		8		8		10	
Range	0-4		0-35		20-jan		0-33		20-jan		0-69		0-50		0-94		feb-15		1-jan		0-62	
**Promyelocyte**		0.18		0.58		0.31		0.25		0.85		0.27		0.69		0.12		0.28		0.27		**0.0011**
Median	0		0		0		0		0		0		0		0		0		0		1	
Range	0-0		0-2		0-6		0-18		0-3		0-8		0-3		0-3		0-0		0-4		0-71	
**Normal karyotype, n (%)**	4 (66.7%)	1	18 (90%)	**0.039**	5 (45.5%)	1	11 (61.1%)	1	6 (85.7%)	0.76	32 (91.4%)	**0.0016**	14 (93.3%)	0.27	8 (53.3%)	1	3 (75%)	1	17 (81%)	**0.049**	8 (53.3%)	1
**Complex karyotype, n (%)**	0 (0%)	1	0 (0%)	1	0 (0%)	1	1 (5.56%)	0.95	0 (0%)	1	1 (2.86%)	1	12 (80%)	**1.30E-09**	1 (6.67%)	0.9	1 (25%)	0.38	5 (23.8%)	0.077	1 (6.67%)	0.92
**Fab class**																						
M0	0 (0%)	1	1 (5%)	0.88	0 (0%)	1	0 (0%)	1	2 (28.6%)	0.14	0 (0%)	1	3 (20%)	0.16	2 (13.3%)	0.43	0 (0%)	1	8 (38.1%)	**0.00011**	0 (0%)	1
M1	5 (83.3%)	**2.80E-03**	8 (40%)	0.06	2 (18.2%)	0.78	5 (27.8%)	0.41	0 (0%)	1	6 (17.1%)	0.89	2 (13.3%)	0.91	6 (40%)	0.1	2 (50%)	0.23	3 (14.3%)	0.91	0 (0%)	1
M2	1 (16.7%)	0.79	7 (35%)	0.13	1 (9.09%)	0.95	12 (66.7%)	**2.80E-05**	0 (0%)	1	4 (11.4%)	0.98	4 (26.7%)	0.46	6 (40%)	0.093	0 (0%)	1	3 (14.3%)	0.9	0 (0%)	1
M3	0 (0%)	1	0 (0%)	1	0 (0%)	1	0 (0%)	1	0 (0%)	1	0 (0%)	1	0 (0%)	1	0 (0%)	1	0 (0%)	1	0 (0%)	1	15 (100%)	**1.10E-21**
M4	0 (0%)	1	2 (10%)	0.95	8 (72.7%)	**1.90E-04**	1 (5.56%)	0.99	0 (0%)	1	14 (40%)	**0.003**	2 (13.3%)	0.87	0 (0%)	1	1 (25%)	0.61	7 (33.3%)	0.12	0 (0%)	1
M5	0 (0%)	1	1 (5%)	0.9	0 (0%)	1	0 (0%)	1	5 (71.4%)	**1.10E-04**	11 (31.4%)	**4.10E-05**	0 (0%)	1	0 (0%)	1	0 (0%)	1	0 (0%)	1	0 (0%)	1
M6	0 (0%)	1	0 (0%)	1	0 (0%)	1	0 (0%)	1	0 (0%)	1	0 (0%)	1	1 (6.67%)	0.17	0 (0%)	1	1 (25%)	**0.047**	0 (0%)	1	0 (0%)	1
M7	0 (0%)	1	0 (0%)	1	0 (0%)	1	0 (0%)	1	0 (0%)	1	0 (0%)	1	3 (20%)	**0.0006**	0 (0%)	1	0 (0%)	1	0 (0%)	1	0 (0%)	1
**Abnormalities, n (%)**																						
FLT3 / NPM1+	4 (66.7%)	**0.002**	2 (10%)	0.73	0 (0%)	1	0 (0%)	1	1 (14.3%)	0.600	12 (34.3%)	**4.80E-05**	0 (0%)	1	1 (6.67%)	0.87	0 (0%)	1	0 (0%)	1	0 (0%)	1
$FLT3^{ITD}$ / NPM1wt	0 (0%)	1	3 (15%)	0.66	2 (18.2%)	0.56	2 (11.1%)	0.83	2 (28.6%)	0.32	8 (22.9%)	0.17	0 (0%)	1	1 (6.67%)	0.94	0 (0%)	1	2 (9.52%)	0.89	7 (46.7%)	**0.0035**
$FLT3^{ITD}$ / NPM1+	2 (33.3%)	0.15	9 (45%)	**6.10E-05**	0 (0%)	1	0 (0%)	1	0 (0%)	1	9 (25.7%)	**0.0088**	0 (0%)	1	0 (0%)	1	0 (0%)	1	0 (0%)	1	0 (0%)	1
FLT3 / NPM1wt	0 (0%)	1	6 (30%)	1	9 (81.8%)	0.11	16 (88.9%)	**0.0056**	4 (57.1%)	0.72	6 (17.1%)	1	15 (100%)	**3.10E-04**	13 (86.7%)	**0.022**	4 (100%)	0.17	19 (90.5%)	**0.0013**	8 (53.3%)	0.79
RAS	1 (16.7%)	0.29	1 (5%)	0.69	0 (0%)	1	0 (0%)	1	0 (0%)	1	4 (11.4%)	0.093	1 (6.67%)	0.58	1 (6.67%)	0.58	0 (0%)	1	1 (4.76%)	0.71	0 (0%)	1
IDH1	4 (66.7%)	**5.90E-04**	0 (0%)	1	0 (0%)	1	0 (0%)	1	0 (0%)	1	3 (8.57%)	0.65	0 (0%)	1	7 (46.7%)	**4.50E-05**	0 (0%)	1	1 (4.76%)	0.88	0 (0%)	1
inv(16)	0 (0%)	1	0 (0%)	1	7 (63.6%)	**5.20E-10**	0 (0%)	1	0 (0%)	1	0 (0%)	1	0 (0%)	1	0 (0%)	1	0 (0%)	1	0 (0%)	1	0 (0%)	1
t(15;17)	0 (0%)	1	0 (0%)	1	0 (0%)	1	0 (0%)	1	0 (0%)	1	0 (0%)	1	0 (0%)	1	0 (0%)	1	0 (0%)	1	0 (0%)	1	14 (93.3%)	**1.70E-19**
t(8;21)	0 (0%)	1	0 (0%)	1	0 (0%)	1	7 (38.9%)	**5.00E-08**	0 (0%)	1	0 (0%)	1	0 (0%)	1	0 (0%)	1	0 (0%)	1	0 (0%)	1	0 (0%)	1
5q	0 (0%)	1	0 (0%)	1	0 (0%)	1	1 (5.56%)	0.81	0 (0%)	1	0 (0%)	1	10 (66.7%)	**7.60E-10**	2 (13.3%)	0.36	0 (0%)	1	1 (4.76%)	0.86	0 (0%)	1
7q	0 (0%)	1	0 (0%)	1	0 (0%)	1	1 (5.56%)	0.9	0 (0%)	1	1 (2.86%)	0.99	9 (60%)	**1.40E-06**	4 (26.7%)	0.073	1 (25%)	0.39	2 (9.52%)	0.73	1 (6.67%)	0.85
Trisomy8	0 (0%)	1	1 (5%)	0.9	1 (9.09%)	0.7	0 (0%)	1	1 (14.3%)	0.54	0 (0%)	1	5 (33.3%)	**9.50E-03**	5 (33.3%)	**0.0095**	0 (0%)	1	2 (9.52%)	0.66	2 (13.3%)	0.47
**Platform**																						
GE		3.05E-09		9.64E-18		2.73E-10		3.68E-07		8.81E-04		7.68E-11		1.08E-13						2.10E-09		1.14E-21
ME		1.97E-06		1.57E-04		3.04E-12		9.79E-05		1.29E-03		2.54E-05		2.93E-08		7.72E-05				4.28E-06		1.34E-17
CN														6.43E-06		9.89E-04		3.20E-08				
MirExp		1.00E-03		1.05E-08		3.29E-09		2.23E-04				2.46E-09		2.20E-09						3.25E-04		3.17E-13

Abbreviations: AML-rest, patients that are not in cluster # i; Number of cases (percentage), median (range) or missing values are depicted were appropriate; Fab class, morphological classification; M0, minimally diffentiated; M1, without maturation; M2, with maturation; M3, hypergranular promyelocytic; M4, myelomonocytic; M5, (a) monoblastic, (b) monocytic; M6, erytroleukemia; AML, acute myeloid leukemia; FLT3ITD, FLT3 internal tandem duplication; NPM1+, nucleophosmin 1; wt, wild-type; neg, negative. P-values indicate the comparison of patients in Cluster #i versus the patients not in cluster #i (AML-rest); Note that percentages are solely based on non-missing values. *P-values are computed using the Mann-Whitney U test (continuous variables) and 2-sided Fisher exact test (categorical variables).

Table S3

Cancer tissues/ Clustering algorithm	MEREDITH		Low Dimensional PCA Space						High dimensional PCA Space	
			PCA(2D) and HC		PCA(3D) and HC		PCA(4D) and HC		Original space (200D) and HC	
	P Enrichment	Clust.nr	P Enrichment	Clust.nr	P Enrichment	Clust.nr	P Enrichment	Clust.nr	P Enrichment	Clust.nr
FLT3wt/NPM1+	0.002, 4.8E-05	1,6	5.00E-05	3	5.60E-05	5	1.50E-04	1	2.2-E04, 0.013	8,9
FLT3ITD/NPM1wt	0.0035	11	-	-	-	-	0.014	3	0.0035	15
FLT3ITD/NPM1+	6.1E-05, 8.8-E03	2,6	2.60E-08	3	1.10E-07	5	1.50E-04	1	0.029, 2.7-E05	8,12
FLT3wt/NPM1wt	0.0056, 3.1-E04	4,7	8.7-E05, 2.2-E04	2,4	0.0018, 1.6-E05	3,4	3.00E-05	4	3.1-E05, 1.7-E04	2,11
RAS	-	-			-	-	-	-	-	-
IDH1	5.9-E04, 4.5-E05	1,8			-	-	-	-	1.30E-05	13
inv(16)	5.20E-10	3	3.50E-03	4	1.30E-05	1	0.0035	2	1.90E-10	9
t(15;17)	1.70E-19	11	2.80E-13	1	0.0082, 7.2E-15	2,6	7.20E-15	4	1.70E-19	15
t(8;21)	5.00E-08	4	3.50E-02	4	0.0055	1	0.0035	4	1.30E-11	4
5q	7.60E-10	7	1.40E-07	2	1.00E-07	3	3.70E-07	3	0.019, 9.2E-12	1,2
7q	1.40E-06	7	6.10E-07	2	0.00059, 0.048	3,4	1.90E-07	3	6.00E-08	2
Trisomy8	9.5-E03, 9.5-E03	7,8	2.20E-03	2	0.022	3	0.00025	3	0.028, 0.05	1,2

Green color depicts highest cluster enrichment with subtype

Table S4: Patient demographics and clinical characteristics of Breast cancer patients in the 5 clusters

	Cl.1 (n=107)	P	Cl.2 (n=69)	P2	Cl.3 (n=82)	P3	Cl.4 (n=92)	P4	Cl.5 (n=213)	P5
Age, years		0.67		0.00016		0.04		0.31		0.86
median	58		67		53		56		59	
range	39-90		39-87		33-86		29-90		27-90	
Sex, n (%)		1		0.18		1		1		0.089
Male	0 (0%)		2 (2.9%)		0 (0%)		0 (0%)		5 (2.3%)	
Female	97 (91%)		57 (83%)		76 (93%)		88 (96%)		203 (95%)	
Menopause status, n (%)										
Indeterminate	1 (0.935%)	0.19	0 (0%)	1	0 (0%)	1	0 (0%)	1	0 (0%)	1
Peri	3 (2.8%)	0.73	0 (0%)	1	5 (6.1%)	0.13	4 (4.35%)	0.38	7 (3.29%)	0.62
Post	56 (52.3%)	0.98	41 (59.4%)	0.67	57 (69.5%)	0.057	55 (59.8%)	0.66	135 (63.4%)	0.22
Pre	26 (24.3%)	0.15	8 (11.6%)	0.99	11 (13.4%)	0.97	19 (20.7%)	0.51	50 (23.5%)	0.085
Pathologic stage, n (%)										
Stage I	5 (4.67%)	0.95	2 (2.9%)	0.98	5 (6.1%)	0.8	14 (15.2%)	0.0061	18 (8.45%)	0.39
Stage IA	7 (6.54%)	0.69	4 (5.8%)	0.77	4 (4.88%)	0.88	6 (6.52%)	0.69	20 (9.39%)	0.092
Stage IB	1 (0.935%)	0.57	2 (2.9%)	0.075	0 (0%)	1	1 (1.09%)	0.51	0 (0%)	1
Stage II	5 (4.67%)	0.0078	0 (0%)	1	0 (0%)	1	1 (1.09%)	0.76	2 (0.939%)	0.87
Stage IIA	16 (15%)	1	22 (31.9%)	0.43	38 (46.3%)	0.00072	31 (33.7%)	0.26	64 (30%)	0.59
Stage IIB	23 (21.5%)	0.64	16 (23.2%)	0.48	20 (24.4%)	0.36	19 (20.7%)	0.71	48 (22.5%)	0.51
Stage III	0 (0%)	1	0 (0%)	1	0 (0%)	1	1 (1.09%)	0.30	1 (0.469%)	0.61
Stage IIIA	18 (16.8%)	0.15	3 (4.35%)	1	6 (7.32%)	0.98	9 (9.78%)	0.9	39 (18.3%)	0.0052
Stage IIIB	2 (1.87%)	0.7	3 (4.35%)	0.17	0 (0%)	1	1 (1.09%)	0.89	6 (2.82%)	0.28
Stage IIIC	19 (17.8%)	1.90E-05	6 (8.7%)	0.36	2 (2.44%)	0.99	4 (4.35%)	0.92	9 (4.23%)	0.99
Stage IV	1 (0.935%)	0.47	1 (1.45%)	0.32	0 (0%)	1	1 (1.09%)	0.42	0 (0%)	1
Stage X	0 (0%)	1	0 (0%)	1	0 (0%)	1	0 (0%)	1	1 (0.469%)	0.38
Histological type, n (%)										
Infiltrating Carcinoma NOS	0 (0%)	1	0 (0%)	1	1 (1.22%)	0.15	0 (0%)	1	0 (0%)	1
Infiltrating Ductal Carcinoma	30 (28%)	1.00	22 (31.9%)	1.00	67 (81.7%)	1.40E-05	62 (67.4%)	0.11	163 (76.5%)	2.20E-09
Infiltrating Lobular Carcinoma	63 (58.9%)	3.70E-21	23 (33.3%)	0.014	1 (1.22%)	1	15 (16.3%)	0.95	22 (10.3%)	1
Medullary Carcinoma	0 (0%)	1.00	0 (0%)	1	4 (4.88%)	0.0019	1 (1.09%)	0.59	0 (0%)	1
Mixed Histology	3 (2.8%)	0.70	2 (2.9%)	0.67	1 (1.22%)	0.94	2 (2.17%)	0.82	10 (4.69%)	0.093
Mucinous Carcinoma	1 (0.935%)	0.92	9 (13%)	6.20E-07	0 (0%)	1	1 (1.09%)	0.89	1 (0.469%)	1
Other	0 (0%)	1	3 (4.35%)	0.58	2 (2.44%)	0.89	7 (7.61%)	0.079	12 (5.63%)	0.15
ER/PR/HER2 status, n (%)										
ER+	90 (84.1%)	1.60E-09	55 (79.7%)	3.70E-06	9 (11%)	1	46 (50%)	1	179 (84%)	3.80E-15
PR+	79 (73.8%)	2.70E-05	51 (73.9%)	5.70E-05	6 (7.32%)	1	38 (41.3%)	1	166 (77.9%)	5.20E-13
HER2+	9 (8.41%)	0.99	6 (8.7%)	1	7 (8.54%)	0.99	18 (19.6%)	0.1	42 (19.7%)	0.00095
Luminal A, n (%)	33 (30.8%)	5.10E-06	21 (30.4%)	6.20E-04	3 (3.66%)	1	7 (7.61%)	1	41 (19.2%)	0.36
ER-/PR+/HER2-	0 (0%)	1	1 (1.45%)	0.23	1 (1.22%)	0.27	0 (0%)	1	0 (0%)	1
ER+/PR-/HER2-	2 (1.87%)	0.53	2 (2.9%)	0.3	2 (2.44%)	0.38	1 (1.09%)	0.82	2 (0.939%)	0.91
ER+/PR+/HER2-	31 (29%)	5.00E-06	18 (26.1%)	0.006	0 (0%)	1	6 (6.52%)	1	39 (18.3%)	0.17
Luminal B, n (%)	9 (8.41%)	0.95	6 (8.7%)	0.89	4 (4.88%)	1	11 (12%)	0.67	38 (17.8%)	1.60E-04
ER-/PR+/HER2+	0 (0%)	1	0 (0%)	1	2 (2.44%)	0.1	0 (0%)	1	2 (0.939%)	0.48
ER+/PR-/HER2+	1 (0.935%)	0.92	2 (2.9%)	0.44	2 (2.44%)	0.54	2 (2.17%)	0.64	5 (2.35%)	0.49
ER+/PR+/HER2+	8 (7.48%)	0.83	4 (5.8%)	0.92	0 (0%)	1	9 (9.78%)	0.56	31 (14.6%)	0.00017
HER2-type, n (%)										
ER-/PR-/HER2+	0 (0%)	1	0 (0%)	1	3 (3.66%)	0.33	7 (7.61%)	0.0035	4 (1.88%)	0.84
Basal, n (%)										
ER-/PR-/HER2-	0 (0%)	1	0 (0%)	1	22 (26.8%)	3.90E-14	13 (14.1%)	1.50E-03	0 (0%)	1
Platform overlap										
GE		2.01E-23		1.27E-11		4.88E-88		3.28E-09		2.90E-31
ME		2.05E-20		7.48E-12		9.91E-79		5.58E-48		1.46E-36
CN		1.75E-10		6.05E-08		8.54E-67		1.75E-11		6.96E-26
MIR		4.06E-19		8.58E-27		1.62E-84		1.14E-04		2.37E-38
Platform contribution										
Various				1.40E-09						
ME						1.15E-16		7.71E-15		
Any				1.77E-03						
CN						6.24E-05				2.73E-03
All		2.67E-04								1.07E-12

Menopause status is defined as following: Indeterminate (neither Pre or Postmenopausal), Peri (6-12 months since last menstrual period), Post (prior bilateral ovariectomy OR >12 mo since LMP with no prior hysterectomy), Pre (<6 months since LMP AND no prior bilateral ovariectomy AND not on estrogen replacement)

Table S5

Cancer tissues/ Clustering algorithm	MEREDITH		Low Dimensional PCA Space						High dimensional PCA Space	
			PCA(2D) and HC		PCA(3D) and HC		PCA(4D) and HC		Original space (200D) and HC	
	P Enrichment	Clust.nr	P Enrichment	Clust.nr	P Enrichment	Clust.nr	P Enrichment	Clust.nr	P Enrichment	Clust.nr
ER+	3.80E-15	1,2,5	2.20E-13	5	9.50E-08	5	5.70E-10	7	1.00E-06	4
PR+	5.20E-13	1,2,5	2.20E-08	5	5.80E-06	5	5.40E-08	3	9.80E-05	7
HER2+	0.00095	5	5.00E-02	4	0.006	1	0.0031	1	0.0033	6
Luminal A	5.10E-06	1	0.0019	6	0.0089	4	0.0014	7	0.00025	8
Luminal B	1.60E-04	5	0.013	4	0.028	7	0.022	5	1.10E-04	6
HER2-type	0.0035	4	0.0092	2	0.019	1	0.032	9	2.00E-06	5
Basal	3.90E-14	3	4.50E-08	1	2.30E-11	7	3.80E-05	9	3.40E-12	12

Green colored cell depicts highest cluster enrichment with subtype

Table S6

Gene-set	Pathway	BY ≤ 0.05
Immunologic signatures	GSE36476_CTRL_VS_TSST_ACT_72H_MEMORY_CD4_TCELL_OLD_DN	7.54E-21
Immunologic signatures	GSE15750_DAY6_VS_DAY10_EFF_CD8_TCELL_UP	1.25E-20
Immunologic signatures	GSE36476_CTRL_VS_TSST_ACT_72H_MEMORY_CD4_TCELL_YOUNG_DN	1.41E-17
Immunologic signatures	GSE15750_DAY6_VS_DAY10_TRAF6KO_EFF_CD8_TCELL_UP	3.82E-17
Immunologic signatures	GSE36476_CTRL_VS_TSST_ACT_40H_MEMORY_CD4_TCELL_OLD_DN	3.82E-17
Immunologic signatures	GSE36476_CTRL_VS_TSST_ACT_40H_MEMORY_CD4_TCELL_YOUNG_DN	6.71E-17
Immunologic signatures	GSE24634_TREG_VS_TCONV_POST_DAY7_IL4_CONVERSION_UP	1.21E-16
Immunologic signatures	GSE22886_UNSTIM_VS_IL2_STIM_NKCELL_DN	9.31E-16
Immunologic signatures	GSE30962_PRIMARY_VS_SECONDARY_ACUTE_LCMV_INF_CD8_TCELL_UP	6.82E-15
Immunologic signatures	GSE24634_TEFF_VS_TCONV_DAY7_IN_CULTURE_UP	1.27E-14
Immunologic signatures	GOLDRATH_EFF_VS_MEMORY_CD8_TCELL_UP	3.37E-13
Immunologic signatures	GSE12845_IGD_POS_BLOOD_VS_PRE_GC_TONSIL_BCELL_DN	2.34E-12
Immunologic signatures	GSE29618_BCELL_VS_MDC_DN	3.94E-12
Immunologic signatures	GSE29614_CTRL_VS_DAY7_TIV_FLU_VACCINE_PBMC_DN	1.45E-11
Immunologic signatures	GSE24634_IL4_VS_CTRL_TREATED_NAIVE_CD4_TCELL_DAY7_UP	1.83E-11
Immunologic signatures	GSE29614_DAY3_VS_DAY7_TIV_FLU_VACCINE_PBMC_DN	2.55E-11
Immunologic signatures	GSE17974_0H_VS_24H_IN_VITRO_ACT_CD4_TCELL_DN	5.32E-11
All canonical pathways	REACTOME_CELL_CYCLE_MITOTIC	1.04E-10
All canonical pathways	REACTOME_METABOLISM_OF_LIPIDS_AND_LIPOPROTEINS	1.04E-10
Immunologic signatures	GSE22886_UNSTIM_VS_IL15_STIM_NKCELL_DN	3.18E-10
All canonical pathways	REACTOME_EXTRACELLULAR_MATRIX_ORGANIZATION	3.74E-10
Immunologic signatures	GSE10325_LUPUS_BCELL_VS_LUPUS_MYELOID_DN	5.14E-10
Immunologic signatures	GSE9988_ANTI_TREM1_AND_LPS_VS_VEHICLE_TREATED_MONOCYTES_DN	5.58E-10
All canonical pathways	KEGG_VALINE_LEUCINE_AND_ISOLEUCINE_DEGRADATION	1.04E-09
All canonical pathways	REACTOME_CELL_CYCLE	2.47E-09
All canonical pathways	KEGG_COMPLEMENT_AND_COAGULATION_CASCADES	2.74E-09
Immunologic signatures	GOLDRATH_NAIVE_VS_EFF_CD8_TCELL_DN	2.92E-09
Immunologic signatures	GSE3982_MEMORY_CD4_TCELL_VS_TH2_DN	4.16E-09
Immunologic signatures	GSE24634_TEFF_VS_TCONV_DAY5_IN_CULTURE_UP	6.58E-09
Oncogenic signatures	RB_P107_DN.V1_UP	9.88E-09
All canonical pathways	REACTOME_DNA_REPLICATION	1.49E-08

All canonical pathways	REACTOME_AXON_GUIDANCE	1.54E-08
All canonical pathways	REACTOME_COLLAGEN_FORMATION	1.82E-08
Oncogenic signatures	CSR_LATE_UP.V1_UP	2.44E-08
Oncogenic signatures	RPS14_DN.V1_DN	2.44E-08
Immunologic signatures	GSE10239_NAIVE_VS_DAY4.5_EFF_CD8_TCELL_DN	2.93E-08
Immunologic signatures	GSE9988_LPS_VS_VEHICLE_TREATED_MONOCYTE_DN	5.32E-08
Immunologic signatures	GSE7852_LN_VS_THYMUS_TCONV_DN	6.49E-08
All canonical pathways	REACTOME_MITOTIC_M_M_G1_PHASES	6.83E-08
All canonical pathways	REACTOME_DEVELOPMENTAL_BIOLOGY	1.02E-07
Immunologic signatures	GSE24634_TREG_VS_TCONV_POST_DAY10_IL4_CONVERSION_DN	1.06E-07
Immunologic signatures	GSE30962_ACUTE_VS_CHRONIC_LCMV_PRIMARY_INF_CD8_TCELL_DN	1.17E-07
Immunologic signatures	GSE9988_ANTI_TREM1_VS_VEHICLE_TREATED_MONOCYTES_DN	1.17E-07
Immunologic signatures	GSE3982_BCELL_VS_TH2_DN	1.42E-07
Immunologic signatures	GSE22886_NAIVE_VS_IGM_MEMORY_BCELL_DN	1.98E-07
Immunologic signatures	GSE29618_BCELL_VS_MDC_DAY7_FLU_VACCINE_DN	1.98E-07
Immunologic signatures	GSE3982_DC_VS_CENT_MEMORY_CD4_TCELL_UP	2.03E-07
Immunologic signatures	KAECH_DAY8_EFF_VS_MEMORY_CD8_TCELL_UP	2.33E-07
Immunologic signatures	GSE20715_0H_VS_48H_OZONE_TLR4_KO_LUNG_DN	2.39E-07
Immunologic signatures	GSE13411_PLASMA_CELL_VS_MEMORY_BCELL_UP	2.61E-07
Immunologic signatures	GSE24634_TREG_VS_TCONV_POST_DAY7_IL4_CONVERSION_DN	2.68E-07
Immunologic signatures	GSE24634_TREG_VS_TCONV_POST_DAY5_IL4_CONVERSION_UP	3.26E-07
Immunologic signatures	GSE31082_CD4_VS_CD8_SP_THYMOCYTE_DN	3.77E-07
All canonical pathways	REACTOME_HEMOSTASIS	4.65E-07
Immunologic signatures	GSE3982_MEMORY_CD4_TCELL_VS_TH1_DN	5.70E-07
Oncogenic signatures	P53_DN.V1_UP	5.98E-07
Immunologic signatures	GSE15930_NAIVE_VS_24H_IN_VITRO_STIM_INFAB_CD8_TCELL_DN	8.35E-07
Immunologic signatures	GSE24634_TREG_VS_TCONV_POST_DAY3_IL4_CONVERSION_UP	8.35E-07
Immunologic signatures	GSE15930_NAIVE_VS_24H_IN_VITRO_STIM_CD8_TCELL_DN	9.85E-07
Immunologic signatures	GSE29618_BCELL_VS_MONOCYTE_DAY7_FLU_VACCINE_DN	9.85E-07
Oncogenic signatures	SNF5_DN.V1_UP	1.14E-06
Immunologic signatures	GSE3982_EOSINOPHIL_VS_TH2_DN	1.15E-06
Immunologic signatures	GSE17721_LPS_VS_CPG_24H_BMDM_DN	1.16E-06
Immunologic signatures	GSE10325_LUPUS_CD4_TCELL_VS_LUPUS_MYELOID_DN	1.40E-06

All canonical pathways	KEGG_PATHOGENIC_ESCHERICHIA_COLI_INFECTION	1.79E-06
Oncogenic signatures	MEL18_DN.V1_UP	1.85E-06
Immunologic signatures	GSE12845_IGD_POS_VS_NEG_BLOOD_BCELL_DN	1.91E-06
Immunologic signatures	GSE22886_DAY0_VS_DAY7_MONOCYTE_IN_CULTURE_DN	1.93E-06
Oncogenic signatures	PRC2_EDD_UP.V1_UP	2.13E-06
Immunologic signatures	GSE15930_NAIVE_VS_24H_IN_VITRO_STIM_IL12_CD8_TCELL_DN	2.29E-06
Immunologic signatures	GSE20366_EX_VIVO_VS_HOMEOSTATIC_CONVERSION_TREG_DN	2.29E-06
Immunologic signatures	GSE36476_CTRL_VS_TSST_ACT_16H_MEMORY_CD4_TCELL_OLD_DN	2.29E-06
Immunologic signatures	KAECH_NAIVE_VS_DAY8_EFF_CD8_TCELL_DN	2.65E-06
Immunologic signatures	GSE12845_IGD_NEG_BLOOD_VS_PRE_GC_TONSIL_BCELL_DN	2.65E-06
Immunologic signatures	GSE34205_HEALTHY_VS_RSV_INF_INFANT_PBMC_DN	2.65E-06
Immunologic signatures	GSE11057_NAIVE_VS_EFF_MEMORY_CD4_TCELL_DN	3.07E-06
Immunologic signatures	GSE24634_NAIVE_CD4_TCELL_VS_DAY7_IL4_CONV_TREG_DN	3.07E-06
Immunologic signatures	GSE3982_CENT_MEMORY_CD4_TCELL_VS_TH1_DN	3.07E-06
Immunologic signatures	GSE18791_CTRL_VS_NEWCASTLE_VIRUS_DC_18H_UP	3.50E-06
Immunologic signatures	GSE15930_NAIVE_VS_48H_IN_VITRO_STIM_CD8_TCELL_DN	3.52E-06
Immunologic signatures	GSE8678_IL7R_LOW_VS_HIGH_EFF_CD8_TCELL_UP	3.52E-06
Immunologic signatures	GSE3982_EFF_MEMORY_CD4_TCELL_VS_TH2_DN	4.22E-06
Immunologic signatures	GSE12366_GC_BCELL_VS_PLASMA_CELL_UP	4.91E-06
Immunologic signatures	GSE17974_1H_VS_72H_UNTREATED_IN_VITRO_CD4_TCELL_DN	4.91E-06
Immunologic signatures	GSE24142_EARLY_THYMIC_PROGENITOR_VS_DN2_THYMOCYTE_FETAL_DN	4.91E-06
Oncogenic signatures	ESC_J1_UP_LATE.V1_UP	5.41E-06
Oncogenic signatures	LEF1_UP.V1_DN	5.41E-06
Immunologic signatures	GSE17974_CTRL_VS_ACT_IL4_AND_ANTI_IL12_24H_CD4_TCELL_DN	5.76E-06
Immunologic signatures	GSE22886_NAIVE_BCELL_VS_MONOCYTE_DN	5.79E-06
Immunologic signatures	GSE10325_BCELL_VS_MYELOID_DN	6.62E-06
Immunologic signatures	GSE14000_4H_VS_16H_LPS_DC_TRANSLATED_RNA_UP	6.62E-06
Immunologic signatures	GSE20715_0H_VS_48H_OZONE_LUNG_DN	6.62E-06
Immunologic signatures	GSE30083_SP1_VS_SP4_THYMOCYTE_UP	6.62E-06
Immunologic signatures	GSE29614_CTRL_VS_TIV_FLU_VACCINE_PBMC_2007_DN	7.29E-06
Immunologic signatures	GSE10239_NAIVE_VS_KLRG1INT_EFF_CD8_TCELL_DN	7.48E-06
Immunologic signatures	GSE30962_ACUTE_VS_CHRONIC_LCMV_SECONDARY_INF_CD8_TCELL_DN	7.48E-06
Immunologic signatures	GSE9988_ANTI_TREM1_VS_CTRL_TREATED_MONOCYTES_DN	7.48E-06

Immunologic signatures	GSE9988_LOW_LPS_VS_VEHICLE_TREATED_MONOCYTE_DN	7.48E-06
Immunologic signatures	GSE22886_DC_VS_MONOCYTE_UP	8.95E-06
Immunologic signatures	GSE10239_NAIVE_VS_KLRG1HIGH_EFF_CD8_TCELL_DN	1.01E-05
Immunologic signatures	GSE11864_CSF1_IFNG_VS_CSF1_IFNG_PAM3CYS_IN_MAC_UP	1.01E-05
Immunologic signatures	GSE12845_NAIVE_VS_PRE_GC_TONSIL_BCELL_DN	1.01E-05
Immunologic signatures	GSE24634_TREG_VS_TCONV_POST_DAY5_IL4_CONVERSION_DN	1.01E-05
Immunologic signatures	GSE24634_TREG_VS_TCONV_POST_DAY10_IL4_CONVERSION_UP	1.01E-05
Immunologic signatures	GSE360_DC_VS_MAC_T_GONDII_DN	1.01E-05
All canonical pathways	REACTOME_PLATELET_ACTIVATION_SIGNALING_AND_AGGREGATION	1.02E-05
Immunologic signatures	GSE17721_0.5H_VS_12H_PAM3CSK4_BMDM_UP	1.19E-05
Immunologic signatures	GSE29618_PDC_VS_MDC_DAY7_FLU_VACCINE_DN	1.19E-05
Immunologic signatures	GSE17721_POLYIC_VS_CPG_8H_BMDM_DN	1.36E-05
Immunologic signatures	GSE17721_0.5H_VS_12H_CPG_BMDM_UP	1.36E-05
Immunologic signatures	GSE17721_0.5H_VS_8H_CPG_BMDM_UP	1.36E-05
Immunologic signatures	GSE22886_NAIVE_VS_IGG_IGA_MEMORY_BCELL_DN	1.36E-05
Immunologic signatures	GSE24634_TEFF_VS_TCONV_DAY10_IN_CULTURE_DN	1.36E-05
All canonical pathways	REACTOME_BRANCHED_CHAIN_AMINO_ACID_CATABOLISM	1.42E-05
All canonical pathways	PID_UPA_UPAR_PATHWAY	1.56E-05
All canonical pathways	REACTOME_METABOLISM_OF_AMINO_ACIDS_AND_DERIVATIVES	1.56E-05
All canonical pathways	KEGG_PROPANOATE_METABOLISM	1.58E-05
Immunologic signatures	GSE17721_ALL_VS_24H_PAM3CSK4_BMDM_UP	1.60E-05
Immunologic signatures	GSE24634_NAIVE_CD4_TCELL_VS_DAY10_IL4_CONV_TREG_DN	1.60E-05
All canonical pathways	REACTOME_COMPLEMENT_CASCADE	1.70E-05
Immunologic signatures	GSE17721_PAM3CSK4_VS_GADIQUIMOD_4H_BMDM_UP	1.84E-05
Immunologic signatures	GSE17721_LPS_VS_GARDIQUIMOD_24H_BMDM_DN	1.84E-05
Immunologic signatures	GSE3982_DC_VS_TH1_DN	1.84E-05
Immunologic signatures	GSE3982_NKCELL_VS_TH2_DN	1.84E-05
Immunologic signatures	GSE6269_E_COLI_VS_STREP_AUREUS_INF_PBMC_DN	1.84E-05
Immunologic signatures	GSE13411_NAIVE_BCELL_VS_PLASMA_CELL_DN	2.13E-05
Immunologic signatures	GSE27786_LIN_NEG_VS_NKCELL_UP	2.13E-05
Immunologic signatures	GSE3982_MAST_CELL_VS_CENT_MEMORY_CD4_TCELL_UP	2.13E-05
Immunologic signatures	GSE9006_TYPE_1_VS_TYPE_2_DIABETES_PBMC_AT_DX_UP	2.13E-05
All canonical pathways	REACTOME_ADAPTIVE_IMMUNE_SYSTEM	2.42E-05

Immunologic signatures	GSE24142_EARLY_THYMIC_PROGENITOR_VS_DN2_THYMOCYTE_ADULT_DN	2.50E-05
Immunologic signatures	GSE12366_GC_VS_MEMORY_BCELL_UP	2.53E-05
Oncogenic signatures	MEK_UP.V1_UP	2.63E-05
All canonical pathways	REACTOME_BIOLOGICAL_OXIDATIONS	2.73E-05
All canonical pathways	REACTOME_MITOTIC_PROMETAPHASE	2.73E-05
Immunologic signatures	GSE30083_SP2_VS_SP4_THYMOCYTE_DN	3.54E-05
Immunologic signatures	GSE3982_BCELL_VS_TH1_DN	3.54E-05
Oncogenic signatures	E2F1_UP.V1_UP	3.54E-05
Immunologic signatures	GSE11057_PBMC_VS_MEM_CD4_TCELL_UP	4.18E-05
Immunologic signatures	GSE15930_NAIVE_VS_72H_IN_VITRO_STIM_IFNAB_CD8_TCELL_DN	4.18E-05
Immunologic signatures	GSE9006_HEALTHY_VS_TYPE_2_DIABETES_PBMC_AT_DX_UP	4.18E-05
Immunologic signatures	GSE29618_MONOCYTE_VS_MDC_DN	4.86E-05
Immunologic signatures	GSE22886_NAIVE_BCELL_VS_BM_PLASMA_CELL_DN	4.91E-05
Immunologic signatures	GSE17721_0.5H_VS_24H_CPG_BMDM_DN	5.63E-05
Immunologic signatures	GSE24142_DN2_VS_DN3_THYMOCYTE_FETAL_DN	5.63E-05
Immunologic signatures	GSE27786_LIN_NEG_VS_BCELL_UP	5.66E-05
Immunologic signatures	GSE37416_0H_VS_48H_F_TULARENSIS_LVS_NEUTROPHIL_UP	5.66E-05
Immunologic signatures	GSE14000_UNSTIM_VS_16H_LPS_DC_TRANSLATED_RNA_UP	5.72E-05
Immunologic signatures	GSE17721_POLYIC_VS_GARDIQUIMOD_24H_BMDM_DN	6.46E-05
Immunologic signatures	GSE18791_CTRL_VS_NEWCASTLE_VIRUS_DC_14H_UP	6.51E-05
Immunologic signatures	GSE20366_CD103_POS_VS_NEG_TREG_KLRG1NEG_UP	6.51E-05
Immunologic signatures	GSE27786_CD8_TCELL_VS_NEUTROPHIL_DN	7.62E-05
Immunologic signatures	GSE339_CD4POS_VS_CD4CD8DN_DC_DN	7.62E-05
Oncogenic signatures	ESC_J1_UP_EARLY.V1_UP	8.37E-05
Immunologic signatures	GSE17721_0.5H_VS_4H_CPG_BMDM_UP	8.89E-05
Immunologic signatures	GSE360_CTRL_VS_L_DONOVANI_DC_UP	8.89E-05
Immunologic signatures	GSE11057_EFF_MEM_VS_CENT_MEM_CD4_TCELL_UP	8.99E-05
Immunologic signatures	GSE13306_TREG_RA_VS_TCONV_RA_UP	8.99E-05
Immunologic signatures	GSE18791_CTRL_VS_NEWCASTLE_VIRUS_DC_10H_UP	9.13E-05
All canonical pathways	PID_INTEGRIN1_PATHWAY	9.15E-05
All canonical pathways	KEGG_CELL_CYCLE	9.76E-05
Immunologic signatures	GSE22886_NAIVE_CD8_TCELL_VS_DC_DN	9.96E-05
Immunologic signatures	GSE24634_TEFF_VS_TCONV_DAY3_IN_CULTURE_UP	9.96E-05

Immunologic signatures	GSE24634_IL4_VS_CTRL_TREATED_NAIVE_CD4_TCELL_DAY10_DN	9.96E-05
Immunologic signatures	GSE3982_MAC_VS_TH2_DN	9.96E-05
Immunologic signatures	GSE29618_PRE_VS_DAY7_FLU_VACCINE_BCELL_UP	0.000101
Immunologic signatures	GSE9988_LPS_VS_LPS_AND_ANTI_TREM1_MONOCYTE_UP	0.000101
Immunologic signatures	GSE17974_0H_VS_4H_IN_VITRO_ACT_CD4_TCELL_DN	0.0001029
Immunologic signatures	GSE360_T_GONDII_VS_B_MALAYI_LOW_DOSE_DC_DN	0.0001143
Immunologic signatures	GSE17721_PAM3CSK4_VS_CPG_8H_BMDM_UP	0.0001154
Immunologic signatures	GSE3982_DC_VS_MAC_DN	0.0001154
Immunologic signatures	GSE3982_EFF_MEMORY_CD4_TCELL_VS_TH1_DN	0.0001154
Immunologic signatures	GSE11057_CD4_CENT_MEM_VS_PBMC_DN	0.0001178
All canonical pathways	REACTOME_PROTEIN_FOLDING	0.0001219
Immunologic signatures	GSE17721_LPS_VS_PAM3CSK4_6H_BMDM_DN	0.0001282
Immunologic signatures	GSE24142_EARLY_THYMIC_PROGENITOR_VS_DN3_THYMOCYTE_UP	0.0001282
Immunologic signatures	GSE24142_EARLY_THYMIC_PROGENITOR_VS_DN3_THYMOCYTE_FETAL_UP	0.0001282
Immunologic signatures	GSE15930_STIM_VS_STIM_AND_IFNAB_48H_CD8_T_CELL_UP	0.0001307
Immunologic signatures	GSE22886_DAY1_VS_DAY7_MONOCYTE_IN_CULTURE_DN	0.0001307
All canonical pathways	PID_FOXM1PATHWAY	0.0001338
Immunologic signatures	GSE13306_TREG_VS_TCONV_DN	0.0001377
Oncogenic signatures	PRC2_EZH2_UP.V1_DN	0.0001459
Immunologic signatures	GSE22886_NAIVE_CD4_TCELL_VS_48H_ACT_TH1_DN	0.0001471
Immunologic signatures	GSE3982_DC_VS_BCELL_UP	0.0001471
Immunologic signatures	GSE7460_CTRL_VS_TGFB_TREATED_ACT_FOXP3_MUT_TCONV_UP	0.0001471
Immunologic signatures	GSE7460_FOXP3_MUT_VS_HET_ACT_TCONV_UP	0.0001471
Immunologic signatures	GSE7852_TREG_VS_TCONV_FAT_DN	0.0001471
Immunologic signatures	GSE9650_EFFECTOR_VS_MEMORY_CD8_TCELL_UP	0.0001471
All canonical pathways	PID_PLK1_PATHWAY	0.0001478
Immunologic signatures	GSE17974_2H_VS_72H_UNTREATED_IN_VITRO_CD4_TCELL_DN	0.0001491
Immunologic signatures	GSE29618_PRE_VS_DAY7_POST_LAIV_FLU_VACCINE_BCELL_DN	0.0001491
Immunologic signatures	GSE37416_0H_VS_24H_F_TULARENSIS_LVS_NEUTROPHIL_UP	0.0001491
All canonical pathways	REACTOME_NCAM_SIGNALING_FOR_NEURITE_OUT_GROWTH	0.0001567
Immunologic signatures	GSE1460_INTRATHYMIC_T_PROGENITOR_VS_NAIVE_CD4_TCELL_CORD_BLOOD_UP	0.0001657
Immunologic signatures	GSE30083_SP2_VS_SP3_THYMOCYTE_DN	0.0001657
Immunologic signatures	GSE360_CTRL_VS_T_GONDII_MAC_UP	0.0001657

Immunologic signatures	GSE3982_MAC_VS_NKCELL_UP	0.0001657
Immunologic signatures	GSE13738_RESTING_VS_BYSTANDER_ACTIVATED_CD4_TCELL_DN	0.0001684
Immunologic signatures	GSE2826_WT_VS_BTK_KO_BCELL_DN	0.0001684
Immunologic signatures	GSE360_L_DONOVANI_VS_T_GONDII_DC_DN	0.0001684
Immunologic signatures	GSE26928_EFF_MEM_VS_CENTR_MEM_CD4_TCELL_UP	0.0001831
All canonical pathways	KEGG_PPAR_SIGNALING_PATHWAY	0.0001863
All canonical pathways	REACTOME_PHASE1_FUNCTIONALIZATION_OF_COMPOUNDS	0.0001863
All canonical pathways	REACTOME_G2_M_CHECKPOINTS	0.0001863
Immunologic signatures	GSE1460_INTRATHYMIC_T_PROGENITOR_VS_NAIVE_CD4_TCELL_ADULT_BLOOD_UP	0.0001866
Immunologic signatures	GSE17721_0.5H_VS_24H_CPG_BMDM_UP	0.0001866
Immunologic signatures	GSE5463_CTRL_VS_DEXAMETHASONE_TREATED_THYMOCYTE_UP	0.0001866
All canonical pathways	PID_HNF3BPATHWAY	0.0001869
All canonical pathways	REACTOME_IMMUNE_SYSTEM	0.0001869
Immunologic signatures	GSE7764_IL15_TREATED_VS_CTRL_NK_CELL_24H_UP	0.0001926
All canonical pathways	KEGG_PEROXISOME	0.0001937
Immunologic signatures	GSE24142_EARLY_THYMIC_PROGENITOR_VS_DN3_THYMOCYTE_FETAL_DN	0.0002152
Immunologic signatures	GSE29618_BCELL_VS_MONOCYTE_DN	0.0002152
Immunologic signatures	GSE10239_MEMORY_VS_KLRG1INT_EFF_CD8_TCELL_DN	0.0002187
Immunologic signatures	GSE13229_IMM_VS_INTMATURE_NKCELL_UP	0.0002187
Immunologic signatures	GSE13306_TREG_VS_TCONV_SPLEEN_DN	0.0002187
Immunologic signatures	GSE17721_LPS_VS_PAM3CSK4_24H_BMDM_DN	0.0002187
Immunologic signatures	GSE24142_DN2_VS_DN3_THYMOCYTE_FETAL_UP	0.0002408
Immunologic signatures	GSE339_CD8POS_VS_CD4CD8DN_DC_DN	0.0002408
Immunologic signatures	GSE360_CTRL_VS_T_GONDII_DC_UP	0.0002408
Immunologic signatures	GSE360_L_DONOVANI_VS_T_GONDII_MAC_DN	0.0002408
Immunologic signatures	GSE17721_POLYIC_VS_CPG_2H_BMDM_DN	0.0002414
Immunologic signatures	GSE17721_POLYIC_VS_CPG_12H_BMDM_DN	0.0002414
Immunologic signatures	GSE24142_EARLY_THYMIC_PROGENITOR_VS_DN3_THYMOCYTE_DN	0.0002414
Immunologic signatures	GSE29618_PRE_VS_DAY7_POST_LAIV_FLU_VACCINE_BCELL_UP	0.0002414
Immunologic signatures	GSE360_T_GONDII_VS_M_TUBERCULOSIS_MAC_UP	0.0002414
Immunologic signatures	GSE36392_EOSINOPHIL_VS_NEUTROPHIL_IL25_TREATED_LUNG_DN	0.0002414
Immunologic signatures	GSE3982_MAC_VS_CENT_MEMORY_CD4_TCELL_UP	0.0002414
All canonical pathways	KEGG_FATTY_ACID_METABOLISM	0.0002483

Immunologic signatures	GSE22886_NAIVE_BCELL_VS_BLOOD_PLASMA_CELL_DN	0.0002498
Oncogenic signatures	STK33_SKM_DN	0.0002542
All canonical pathways	PID_SYNDECAN_1_PATHWAY	0.0002605
Immunologic signatures	GSE12845_IGD_NEG_BLOOD_VS_DARKZONE_GC_TONSIL_BCELL_DN	0.0002755
Immunologic signatures	GSE17721_LPS_VS_POLYIC_24H_BMDM_UP	0.0002755
Immunologic signatures	GSE24142_DN2_VS_DN3_THYMOCYTE_ADULT_UP	0.0002755
Immunologic signatures	GSE2826_WT_VS_XID_BCELL_UP	0.0002755
All canonical pathways	REACTOME_CELL_SURFACE_INTERACTIONS_AT_THE_VASCULAR_WALL	0.0002815
Immunologic signatures	GSE27786_ERYTHROBLAST_VS_NEUTROPHIL_UP	0.0003158
Immunologic signatures	GSE360_L_DONOVANI_VS_T_GONDII_DC_UP	0.0003158
Immunologic signatures	GSE3982_MAC_VS_BASOPHIL_UP	0.0003158
Immunologic signatures	GSE3982_NKCELL_VS_TH1_DN	0.0003158
All canonical pathways	REACTOME_NCAM1_INTERACTIONS	0.0003194
Oncogenic signatures	BMI1_DN.V1_UP	0.0003205
Immunologic signatures	GSE9988_LPS_VS_CTRL_TREATED_MONOCYTE_DN	0.0003265
Immunologic signatures	GSE9988_ANTI_TREM1_AND_LPS_VS_CTRL_TREATED_MONOCYTES_DN	0.0003265
Immunologic signatures	GSE15659_TREG_VS_TCONV_DN	0.0003276
Immunologic signatures	GSE1460_CORD_VS_ADULT_BLOOD_NAIVE_CD4_TCELL_UP	0.0003334
Immunologic signatures	GSE2706_2H_VS_8H_R848_STIM_DC_UP	0.0003334
Immunologic signatures	GSE31082_DP_VS_CD4_SP_THYMOCYTE_UP	0.0003334
All canonical pathways	PID_INTEGRIN3_PATHWAY	0.0003427
Immunologic signatures	GSE24142_EARLY_THYMIC_PROGENITOR_VS_DN2_THYMOCYTE_DN	0.0003507
Immunologic signatures	GSE360_CTRL_VS_L_DONOVANI_MAC_UP	0.0003507
Immunologic signatures	GSE360_T_GONDII_VS_B_MALAYI_HIGH_DOSE_DC_DN	0.0003507
Immunologic signatures	GSE7764_NKCELL_VS_SPLENOCYTE_DN	0.0003507
Immunologic signatures	GSE20715_WT_VS_TLR4_KO_LUNG_DN	0.0003617
Immunologic signatures	GSE32423_IL7_VS_IL4_MEMORY_CD8_TCELL_UP	0.0003617
Immunologic signatures	GSE3982_MAST_CELL_VS_TH2_UP	0.0003617
Immunologic signatures	GSE13411_IGM_MEMORY_BCELL_VS_PLASMA_CELL_DN	0.0003741
Immunologic signatures	GSE31082_DN_VS_CD8_SP_THYMOCYTE_UP	0.0003741
Oncogenic signatures	ESC_V6.5_UP_EARLY.V1_DN	0.0003813
Immunologic signatures	GSE29618_MONOCYTE_VS_PDC_DAY7_FLU_VACCINE_UP	0.0003954
Immunologic signatures	GSE339_CD8POS_VS_CD4CD8DN_DC_IN_CULTURE_DN	0.0003954

Oncogenic signatures	STK33_DN	0.0004014
Immunologic signatures	GSE14769_20MIN_VS_360MIN_LPS_BMDM_UP	0.0004069
Immunologic signatures	GSE15930_NAIVE_VS_48H_IN_VITRO_STIM_IL12_CD8_TCELL_DN	0.0004069
Immunologic signatures	GSE17721_0.5H_VS_8H_CPG_BMDM_DN	0.0004069
Immunologic signatures	GSE7852_THYMUS_VS_FAT_TCONV_UP	0.0004069
All canonical pathways	KEGG_FOCAL_ADHESION	0.0004103
Immunologic signatures	GSE24142_DN2_VS_DN3_THYMOCYTE_UP	0.0004498
Immunologic signatures	GSE17721_POLYIC_VS_CPG_16H_BMDM_DN	0.0004682
Immunologic signatures	GSE339_EX_VIVO_VS_IN_CULTURE_CD8POS_DC_UP	0.0004682
Immunologic signatures	GSE17974_0H_VS_72H_IN_VITRO_ACT_CD4_TCELL_DN	0.0004887
Immunologic signatures	GSE19825_NAIVE_VS_DAY3_EFF_CD8_TCELL_DN	0.0005091
Immunologic signatures	GSE360_LOW_DOSE_B_MALAYI_VS_M_TUBERCULOSIS_DC_UP	0.000511
Immunologic signatures	GSE10325_CD4_TCELL_VS_MYELOID_DN	0.0005258
Immunologic signatures	GSE14350_TREG_VS_TEFF_DN	0.0005258
Immunologic signatures	GSE17721_4_VS_24H_CPG_BMDM_UP	0.0005258
Immunologic signatures	GSE22886_TH1_VS_TH2_12H_ACT_UP	0.0005258
Immunologic signatures	GSE7764_NKCELL_VS_SPLENOCYTE_UP	0.0005258
Immunologic signatures	GSE17721_LPS_VS_CPG_4H_BMDM_DN	0.0005476
Immunologic signatures	GSE27786_NKCELL_VS_MONO_MAC_DN	0.0005476
Oncogenic signatures	RPS14_DN.V1_UP	0.0005599
Oncogenic signatures	TGFB_UP.V1_UP	0.0005599
Oncogenic signatures	KRAS.DF.V1_UP	0.0005889
Immunologic signatures	GSE20715_WT_VS_TLR4_KO_24H_OZONE_LUNG_UP	0.0006008
Immunologic signatures	GSE27786_NKTCELL_VS_ERYTHROBLAST_DN	0.0006008
Immunologic signatures	GSE17721_POLYIC_VS_PAM3CSK4_12H_BMDM_DN	0.0006297
Oncogenic signatures	HINATA_NFKB_MATRIX	0.000635
Immunologic signatures	GSE17974_CTRL_VS_ACT_IL4_AND_ANTI_IL12_4H_CD4_TCELL_DN	0.0006587
Immunologic signatures	GSE1432_6H_VS_24H_IFNG_MICROGLIA_UP	0.0006825
Immunologic signatures	GSE27786_NEUTROPHIL_VS_MONO_MAC_DN	0.0006825
Immunologic signatures	GSE9037_CTRL_VS_LPS_4H_STIM_BMDM_UP	0.0006825
Immunologic signatures	GSE10325_BCELL_VS_LUPUS_BCELL_UP	0.0007136
Immunologic signatures	GSE25087_FETAL_VS_ADULT_TREG_UP	0.0007136
Oncogenic signatures	LEF1_UP.V1_UP	0.0007206

Immunologic signatures	GSE11864_UNTREATED_VS_CSF1_IFNG_PAM3CYS_IN_MAC_UP	0.0007759
Immunologic signatures	GSE14769_UNSTIM_VS_120MIN_LPS_BMDM_UP	0.0007759
Immunologic signatures	GSE22886_NAIVE_CD8_TCELL_VS_MONOCYTE_DN	0.0007759
Immunologic signatures	GSE24634_IL4_VS_CTRL_TREATED_NAIVE_CD4_TCELL_DAY5_UP	0.0007759
Immunologic signatures	GSE14308_TH1_VS_NATURAL_TREG_DN	0.0007912
Immunologic signatures	GSE17974_0H_VS_6H_IN_VITRO_ACT_CD4_TCELL_DN	0.0007912
Immunologic signatures	GSE24634_TEFF_VS_TCONV_DAY10_IN_CULTURE_UP	0.0007912
Immunologic signatures	GSE25087_FETAL_VS_ADULT_TCONV_UP	0.0007912
Immunologic signatures	GSE27786_BCELL_VS_NKTCELL_DN	0.0007912
Immunologic signatures	GSE29618_LAIV_VS_TIV_FLU_VACCINE_DAY7_MONOCYTE_DN	0.0007912
Immunologic signatures	GSE3982_CTRL_VS_LPS_4H_MAC_UP	0.0007912
All canonical pathways	REACTOME_FATTY_ACID_TRIACYLGLYCEROL_AND_KETONE_BODY_METABOLISM	0.0008338
Oncogenic signatures	CYCLIN_D1_UP.V1_UP	0.0008731
Oncogenic signatures	BMI1_DN_MEL18_DN.V1_UP	0.0008731
All canonical pathways	KEGG_BUTANOATE_METABOLISM	0.0008783
Immunologic signatures	GSE17721_CPG_VS_GARDIQUIMOD_4H_BMDM_DN	0.0009061
Immunologic signatures	GSE3982_EOSINOPHIL_VS_MAC_DN	0.0009061
Immunologic signatures	GSE7852_THYMUS_VS_FAT_TREG_DN	0.0009061
Immunologic signatures	GSE11864_CSF1_VS_CSF1_PAM3CYS_IN_MAC_DN	0.000908
Immunologic signatures	GSE17721_CTRL_VS_LPS_0.5H_BMDM_UP	0.0009458
Immunologic signatures	GSE32423_MEMORY_VS_NAIVE_CD8_TCELL_IL7_UP	0.0009458
Oncogenic signatures	PKCA_DN.V1_UP	0.0009572
Immunologic signatures	GSE3337_CTRL_VS_16H_IFNG_IN_CD8POS_DC_UP	0.0009735
Immunologic signatures	GSE17721_LPS_VS_GARDIQUIMOD_12H_BMDM_DN	0.001004
Immunologic signatures	GSE17721_0.5H_VS_8H_POLYIC_BMDM_UP	0.001004
Immunologic signatures	GSE22886_NAIVE_BCELL_VS_NEUTROPHIL_DN	0.001004
Immunologic signatures	GSE27786_CD8_TCELL_VS_NKCELL_UP	0.001004
Immunologic signatures	GSE37416_0H_VS_12H_F_TULARENSIS_LVS_NEUTROPHIL_UP	0.001004
Immunologic signatures	GSE3982_MAST_CELL_VS_DC_DN	0.001004
Immunologic signatures	GSE39820_TGFBETA1_IL6_VS_TGFBETA1_IL6_IL23A_TREATED_CD4_TCELL_DN	0.001004
Immunologic signatures	GSE37416_12H_VS_24H_F_TULARENSIS_LVS_NEUTROPHIL_UP	0.001058
Immunologic signatures	GSE17721_CTRL_VS_GARDIQUIMOD_4H_BMDM_UP	0.001151
Oncogenic signatures	MYC_UP.V1_UP	0.001185

Immunologic signatures	GSE17721_PAM3CSK4_VS_CPG_12H_BMDM_UP	0.001215
Immunologic signatures	GSE11864_CSF1_VS_CSF1_IFNG_PAM3CYS_IN_MAC_DN	0.001282
Immunologic signatures	GSE17721_LPS_VS_CPG_0.5H_BMDM_UP	0.001303
Immunologic signatures	GSE27786_NKTCELL_VS_MONO_MAC_DN	0.001303
Immunologic signatures	GSE3982_MAC_VS_TH2_UP	0.001303
Immunologic signatures	GSE13738_RESTING_VS_TCR_ACTIVATED_CD4_TCELL_DN	0.001363
Immunologic signatures	GSE27786_LSK_VS_NKTCELL_UP	0.001363
Immunologic signatures	GSE27786_LSK_VS_NEUTROPHIL_UP	0.001363
Immunologic signatures	GSE27786_CD8_TCELL_VS_NKCELL_DN	0.001363
Immunologic signatures	GSE1432_CTRL_VS_IFNG_1H_MICROGLIA_UP	0.001439
Immunologic signatures	GSE17721_PAM3CSK4_VS_GADIQUIMOD_0.5H_BMDM_DN	0.001461
Immunologic signatures	GSE360_DC_VS_MAC_L_DONOVANI_DN	0.001461
Oncogenic signatures	GCNP_SHH_UP_LATE.V1_DN	0.001531
Immunologic signatures	GSE10239_KLRG1INT_VS_KLRG1HIGH_EFF_CD8_TCELL_UP	0.001532
Immunologic signatures	GSE15324_ELF4_KO_VS_WT_NAIVE_CD8_TCELL_UP	0.001532
Immunologic signatures	GSE17721_LPS_VS_POLYIC_8H_BMDM_UP	0.001532
Immunologic signatures	GSE17721_LPS_VS_GARDIQUIMOD_16H_BMDM_DN	0.001532
All canonical pathways	REACTOME_MITOTIC_G1_G1_S_PHASES	0.00154
Immunologic signatures	GSE15930_NAIVE_VS_72H_IN_VITRO_STIM_IL12_CD8_TCELL_DN	0.001621
Immunologic signatures	GSE20715_WT_VS_TLR4_KO_48H_OZONE_LUNG_UP	0.001621
Immunologic signatures	GSE20715_0H_VS_24H_OZONE_LUNG_DN	0.001621
Immunologic signatures	GSE24634_NAIVE_CD4_TCELL_VS_DAY5_IL4_CONV_TREG_DN	0.001621
Immunologic signatures	GSE339_CD4POS_VS_CD8POS_DC_UP	0.001621
Immunologic signatures	GSE360_L_DONOVANI_VS_B_MALAYI_HIGH_DOSE_MAC_DN	0.001621
Immunologic signatures	KAECH_DAY15_EFF_VS_MEMORY_CD8_TCELL_UP	0.001656
Immunologic signatures	GSE11057_NAIVE_CD4_VS_PBMC_CD4_TCELL_DN	0.001656
Immunologic signatures	GSE17721_LPS_VS_POLYIC_1H_BMDM_DN	0.001656
Immunologic signatures	GSE17721_LPS_VS_PAM3CSK4_2H_BMDM_DN	0.001656
Immunologic signatures	GSE17721_POLYIC_VS_CPG_4H_BMDM_DN	0.001656
Immunologic signatures	GSE24102_GRANULOCYSTIC_MDSC_VS_NEUTROPHIL_DN	0.001656
Immunologic signatures	GSE26928_NAIVE_VS_CENT_MEMORY_CD4_TCELL_DN	0.001656
Immunologic signatures	GSE2706_2H_VS_8H_R848_AND_LPS_STIM_DC_UP	0.001656
Immunologic signatures	GSE27786_BCELL_VS_CD8_TCELL_DN	0.001656

Immunologic signatures	GSE339_EX_VIVO_VS_IN_CULTURE_CD4CD8DN_DC_UP	0.001656
Immunologic signatures	GSE3982_CENT_MEMORY_CD4_TCELL_VS_TH2_DN	0.001656
Immunologic signatures	GSE9650_GP33_VS_GP276_LCMV_SPECIFIC_EXHAUSTED_CD8_TCELL_DN	0.001656
All canonical pathways	REACTOME_G1_S_TRANSITION	0.001697
Immunologic signatures	GSE6269_STREP_AUREUS_VS_STREP_PNEUMO_INF_PBMC_DN	0.001709
All canonical pathways	REACTOME_CELL_CYCLE_CHECKPOINTS	0.001712
Oncogenic signatures	EGFR_UP.V1_DN	0.001734
Immunologic signatures	GSE24634_NAIVE_CD4_TCELL_VS_DAY10_IL4_CONV_TREG_UP	0.001738
Immunologic signatures	GSE37416_CTRL_VS_24H_F_TULARENSIS_LVS_NEUTROPHIL_UP	0.001738
Immunologic signatures	GSE7400_CTRL_VS_CSF3_IN_VIVO_TREATED_PBMC_DN	0.001738
Immunologic signatures	KAECH_NAIVE_VS_DAY15_EFF_CD8_TCELL_DN	0.001847
Immunologic signatures	GSE11864_CSF1_IFNG_VS_CSF1_PAM3CYS_IN_MAC_UP	0.001847
Immunologic signatures	GSE17721_PAM3CSK4_VS_GADIQUIMOD_8H_BMDM_UP	0.001847
Immunologic signatures	GSE24634_NAIVE_CD4_TCELL_VS_DAY3_IL4_CONV_TREG_DN	0.001847
Immunologic signatures	GSE29618_LAIV_VS_TIV_FLU_VACCINE_DAY7_BCELL_DN	0.001847
Immunologic signatures	GSE17721_POLYIC_VS_PAM3CSK4_12H_BMDM_UP	0.001948
Immunologic signatures	GSE17974_CTRL_VS_ACT_IL4_AND_ANTI_IL12_12H_CD4_TCELL_DN	0.001948
Immunologic signatures	GSE37416_CTRL_VS_12H_F_TULARENSIS_LVS_NEUTROPHIL_UP	0.001948
Oncogenic signatures	HOXA9_DN.V1_DN	0.00205
Oncogenic signatures	BRCA1_DN.V1_DN	0.002059
Oncogenic signatures	KRAS.DF.V1_DN	0.002059
Immunologic signatures	GOLDRATH_NAIVE_VS_MEMORY_CD8_TCELL_UP	0.002059
Immunologic signatures	GSE14769_UNSTIM_VS_240MIN_LPS_BMDM_UP	0.002059
Immunologic signatures	GSE17721_LPS_VS_POLYIC_24H_BMDM_DN	0.002059
Immunologic signatures	GSE17721_POLYIC_VS_PAM3CSK4_2H_BMDM_DN	0.002059
Immunologic signatures	GSE17721_POLYIC_VS_GARDIQUIMOD_16H_BMDM_DN	0.002059
Immunologic signatures	GSE3982_MAST_CELL_VS_BCELL_UP	0.002059
Immunologic signatures	GSE17721_PAM3CSK4_VS_CPG_4H_BMDM_UP	0.002183
Immunologic signatures	GSE3982_EFF_MEMORY_VS_CENT_MEMORY_CD4_TCELL_UP	0.002183
All canonical pathways	PID_MYC_ACTIVPATHWAY	0.00221
All canonical pathways	REACTOME_PHOSPHOLIPID_METABOLISM	0.002213
Oncogenic signatures	CYCLIN_D1_KE_.V1_UP	0.002299
Oncogenic signatures	PRC2_EZH2_UP.V1_UP	0.002299

Oncogenic signatures	ESC_V6.5_UP_LATE.V1_UP	0.002299
Immunologic signatures	GSE36476_YOUNG_VS_OLD_DONOR_MEMORY_CD4_TCELL_16H_TSST_ACT_UP	0.002319
All canonical pathways	KEGG_ECM_RECEPTOR_INTERACTION	0.002322
Immunologic signatures	GSE3982_MAST_CELL_VS_NKCELL_UP	0.002322
Immunologic signatures	GSE3982_NEUTROPHIL_VS_CENT_MEMORY_CD4_TCELL_UP	0.002322
All canonical pathways	REACTOME_METABOLISM_OF_CARBOHYDRATES	0.002414
Immunologic signatures	GSE13411_NAIVE_VS_IGM_MEMORY_BCELL_DN	0.00245
Immunologic signatures	GSE17721_LPS_VS_CPG_2H_BMDM_DN	0.00245
Immunologic signatures	GSE25087_TREG_VS_TCONV_FETUS_UP	0.00245
Immunologic signatures	GSE37416_0H_VS_3H_F_TULARENSIS_LVS_NEUTROPHIL_DN	0.00245
Oncogenic signatures	PIGF_UP.V1_DN	0.002483
Oncogenic signatures	NFE2L2.V2	0.002483
Immunologic signatures	GSE15324_ELF4_KO_VS_WT_ACTIVATED_CD8_TCELL_DN	0.002583
Immunologic signatures	GSE17721_12H_VS_24H_GARDIQUIMOD_BMDM_UP	0.002583
Immunologic signatures	GSE22886_NAIVE_CD4_TCELL_VS_DC_DN	0.002583
Immunologic signatures	GSE339_EX_VIVO_VS_IN_CULTURE_CD4POS_DC_UP	0.002583
Immunologic signatures	GSE3982_BASOPHIL_VS_TH1_DN	0.002583
Immunologic signatures	GSE14308_TH2_VS_TH17_UP	0.002698
Immunologic signatures	GSE17721_POLYIC_VS_PAM3CSK4_24H_BMDM_UP	0.002698
Immunologic signatures	GSE17721_LPS_VS_PAM3CSK4_16H_BMDM_DN	0.002698
Immunologic signatures	GSE29618_MONOCYTE_VS_PDC_UP	0.002698
Immunologic signatures	GSE360_DC_VS_MAC_T_GONDII_UP	0.002698
Immunologic signatures	GSE360_HIGH_VS_LOW_DOSE_B_MALAYI_DC_UP	0.002698
Immunologic signatures	GSE37416_0H_VS_3H_F_TULARENSIS_LVS_NEUTROPHIL_UP	0.002698
Immunologic signatures	GSE7460_CD8_TCELL_VS_TREG_ACT_DN	0.002698
All canonical pathways	BIOCARTA_COMP_PATHWAY	0.002767
Oncogenic signatures	IL2_UP.V1_UP	0.002957
Immunologic signatures	GSE17721_CTRL_VS_CPG_2H_BMDM_DN	0.00305
Immunologic signatures	GSE27786_LSK_VS_CD4_TCELL_UP	0.00305
Immunologic signatures	GSE339_CD8POS_VS_CD4CD8DN_DC_IN_CULTURE_UP	0.00305
Immunologic signatures	GSE8868_SPLEEN_VS_INTESTINE_CD11B_POS_CD11C_NEG_DC_DN	0.00305
Oncogenic signatures	VEGF_A_UP.V1_DN	0.003061
Oncogenic signatures	MTOR_UP.V1_UP	0.003187

Immunologic signatures	GSE12366_GC_VS_NAIVE_BCELL_UP	0.003207
Immunologic signatures	GSE13306_RA_VS_UNTREATED_MEM_CD4_TCELL_UP	0.003207
Immunologic signatures	GSE18791_UNSTIM_VS_NEWCATSLE_VIRUS_DC_10H_UP	0.003207
Immunologic signatures	GSE22886_NAIVE_TCELL_VS_DC_DN	0.003207
Immunologic signatures	GSE2706_UNSTIM_VS_8H_LPS_AND_R848_DC_UP	0.003207
Immunologic signatures	GSE360_DC_VS_MAC_B_MALAYI_HIGH_DOSE_DN	0.003207
All canonical pathways	REACTOME_MRNA_SPLICING	0.003319
Immunologic signatures	GSE11924_TFH_VS_TH17_CD4_TCELL_DN	0.003354
Immunologic signatures	GSE15930_NAIVE_VS_72H_IN_VITRO_STIM_TRICHOSTATINA_CD8_TCELL_DN	0.003354
Immunologic signatures	GSE17721_POLYIC_VS_PAM3CSK4_6H_BMDM_DN	0.003354
Immunologic signatures	GSE17721_PAM3CSK4_VS_GADIQUIMOD_12H_BMDM_UP	0.003354
Immunologic signatures	GSE22886_DAY0_VS_DAY1_MONOCYTE_IN_CULTURE_DN	0.003354
Immunologic signatures	GSE2826_WT_VS_XID_BCELL_DN	0.003354
Immunologic signatures	GSE39820_CTRL_VS_TGFBETA1_IL6_CD4_TCELL_DN	0.003354
All canonical pathways	REACTOME_CYTOCHROME_P450_ARRANGED_BY_SUBSTRATE_TYPE	0.003446
Immunologic signatures	GSE13411_SWITCHED_MEMORY_BCELL_VS_PLASMA_CELL_DN	0.003554
Immunologic signatures	GSE14308_TH17_VS_INDUCED_TREG_DN	0.003554
Immunologic signatures	GSE17721_POLYIC_VS_PAM3CSK4_4H_BMDM_DN	0.003554
Immunologic signatures	GSE20366_CD103_KLRG1_DP_VS_DN_TREG_DN	0.003554
Immunologic signatures	GSE17721_CTRL_VS_LPS_24H_BMDM_DN	0.003718
Immunologic signatures	GSE17721_CTRL_VS_PAM3CSK4_12H_BMDM_UP	0.003718
Immunologic signatures	GSE17721_LPS_VS_POLYIC_12H_BMDM_UP	0.003718
Immunologic signatures	GSE3982_MAST_CELL_VS_NEUTROPHIL_DN	0.003718
Immunologic signatures	GSE3982_NEUTROPHIL_VS_TH1_DN	0.003718
Immunologic signatures	GSE9006_TYPE_1_DIABETES_AT_DX_VS_1MONTH_POST_DX_PBMC_UP	0.003718
Immunologic signatures	GSE18791_CTRL_VS_NEWCASTLE_VIRUS_DC_6H_UP	0.003722
Immunologic signatures	GSE2706_UNSTIM_VS_8H_R848_DC_UP	0.003722
All canonical pathways	REACTOME_GLYCEROPHOSPHOLIPID_BIOSYNTHESIS	0.00386
All canonical pathways	REACTOME_INTEGRATION_OF_ENERGY_METABOLISM	0.00386
All canonical pathways	REACTOME_S_PHASE	0.00386
Immunologic signatures	GSE12366_GC_VS_NAIVE_BCELL_DN	0.003936
Immunologic signatures	GSE17721_POLYIC_VS_GARDIQUIMOD_12H_BMDM_DN	0.003936
Immunologic signatures	GSE3982_BCELL_VS_TH1_UP	0.003936

Immunologic signatures	GSE15930_STIM_VS_STIM_AND_TRICHOSTATINA_48H_CD8_T_CELL_UP	0.00413
Immunologic signatures	GSE24634_TREG_VS_TCONV_POST_DAY3_IL4_CONVERSION_DN	0.00413
Immunologic signatures	GSE2826_XID_VS_BTK_KO_BCELL_DN	0.00413
Immunologic signatures	GSE29618_MONOCYTE_VS_MDC_UP	0.00413
Oncogenic signatures	PTEN_DN.V2_UP	0.004155
Immunologic signatures	GSE17974_0H_VS_2H_IN_VITRO_ACT_CD4_TCELL_DN	0.004164
Immunologic signatures	GSE1448_CTRL_VS_ANTI_VALPHA2_DP_THYMOCYTE_UP	0.004368
Immunologic signatures	GSE1460_NAIVE_CD4_TCELL_ADULT_BLOOD_VS_THYMIC_STROMAL_CELL_DN	0.004368
Immunologic signatures	GSE20366_EX_VIVO_VS_HOMEOSTATIC_CONVERSION_NAIVE_CD4_TCELL_DN	0.004368
Immunologic signatures	GSE27786_BCELL_VS_CD4_TCELL_UP	0.004368
Immunologic signatures	GSE9988_LOW_LPS_VS_CTRL_TREATED_MONOCYTE_DN	0.004368
All canonical pathways	REACTOME_PREFOLDIN_MEDIATED_TRANSFER_OF_SUBSTRATE_TO_CCT_TRIC	0.004425
Oncogenic signatures	GCNP_SHH_UP_LATE.V1_UP	0.004565
All canonical pathways	REACTOME_TRANSMEMBRANE_TRANSPORT_OF_SMALL_MOLECULES	0.004568
Immunologic signatures	GSE17580_TREG_VS_TEFF_S_MANSONI_INF_UP	0.004572
Immunologic signatures	GSE17721_LPS_VS_PAM3CSK4_0.5H_BMDM_DN	0.004572
Immunologic signatures	GSE24142_EARLY_THYMIC_PROGENITOR_VS_DN2_THYMOCYTE_UP	0.004572
Immunologic signatures	GSE27786_CD4_TCELL_VS_MONO_MAC_DN	0.004572
Immunologic signatures	GSE14000_UNSTIM_VS_4H_LPS_DC_UP	0.00458
Immunologic signatures	GSE17721_POLYIC_VS_GARDIQUIMOD_8H_BMDM_DN	0.00458
Immunologic signatures	GSE27786_LSK_VS_ERYTHROBLAST_DN	0.00458
Immunologic signatures	GSE3982_BASOPHIL_VS_NKCELL_UP	0.00458
Immunologic signatures	GSE9037_CTRL_VS_LPS_4H_STIM_IRAK4_KO_BMDM_UP	0.00458
Immunologic signatures	GSE14308_TH2_VS_NATURAL_TREG_DN	0.004756
Immunologic signatures	GSE17721_CTRL_VS_CPG_4H_BMDM_UP	0.004756
Immunologic signatures	GSE17721_PAM3CSK4_VS_CPG_16H_BMDM_DN	0.004756
Immunologic signatures	GSE17721_POLYIC_VS_GARDIQUIMOD_4H_BMDM_DN	0.004756
Immunologic signatures	GSE17721_0.5H_VS_12H_POLYIC_BMDM_UP	0.004756
Immunologic signatures	GSE27786_LSK_VS_CD8_TCELL_UP	0.004756
Immunologic signatures	GSE32423_CTRL_VS_IL7_MEMORY_CD8_TCELL_UP	0.004756
Immunologic signatures	GSE7460_TREG_VS_TCONV_ACT_WITH_TGFB_UP	0.004756
All canonical pathways	PID_AURORA_B_PATHWAY	0.004807
All canonical pathways	REACTOME_MHC_CLASS_II_ANTIGEN_PRESENTATION	0.004807

All canonical pathways	REACTOME_METABOLISM_OF_PROTEINS	0.004807
All canonical pathways	REACTOME_ACTIVATION_OF_ATR_IN_RESPONSE_TO_REPLICATION_STRESS	0.004807
Immunologic signatures	GSE22886_CTRL_VS_LPS_24H_DC_UP	0.004979
Immunologic signatures	GSE24142_DN2_VS_DN3_THYMOCYTE_DN	0.004979
Immunologic signatures	GSE3982_EOSINOPHIL_VS_TH1_DN	0.004979
Oncogenic signatures	TBK1.DF_DN	0.004983
Immunologic signatures	GSE14308_TH1_VS_NAIVE_CD4_TCELL_UP	0.005024
Immunologic signatures	GSE17721_POLYIC_VS_PAM3CSK4_16H_BMDM_DN	0.005024
Immunologic signatures	GSE34205_RSV_VS_FLU_INF_INFANT_PBMC_DN	0.005024
Immunologic signatures	GSE9988_LOW_LPS_VS_ANTI_TREM1_AND_LPS_MONOCYTE_UP	0.005024
Immunologic signatures	GSE11057_NAIVE_VS_MEMORY_CD4_TCELL_DN	0.005168
Immunologic signatures	GSE13306_RA_VS_UNTREATED_TCONV_UP	0.005168
Immunologic signatures	GSE14350_IL2RB_KO_VS_WT_TEFF_DN	0.005168
Immunologic signatures	GSE17721_CTRL_VS_POLYIC_12H_BMDM_DN	0.005168
Immunologic signatures	GSE17721_CTRL_VS_CPG_1H_BMDM_UP	0.005168
Immunologic signatures	GSE17721_12H_VS_24H_CPG_BMDM_UP	0.005168
Immunologic signatures	GSE20715_0H_VS_24H_OZONE_TLR4_KO_LUNG_UP	0.005168
Immunologic signatures	GSE24634_TEFF_VS_TCONV_DAY7_IN_CULTURE_DN	0.005168
Immunologic signatures	GSE30962_ACUTE_VS_CHRONIC_LCMV_PRIMARY_INF_CD8_TCELL_UP	0.005168
Immunologic signatures	GSE36476_CTRL_VS_TSST_ACT_16H_MEMORY_CD4_TCELL_YOUNG_DN	0.005168
Immunologic signatures	GSE3982_DC_VS_TH2_UP	0.005168
Immunologic signatures	GSE17721_LPS_VS_POLYIC_2H_BMDM_UP	0.005424
All canonical pathways	REACTOME_PROCESSING_OF_CAPPED_INTRON_CONTAINING_PRE_MRNA	0.005496
All canonical pathways	REACTOME_METABOLISM_OF_NUCLEOTIDES	0.005496
Immunologic signatures	GSE14308_NAIVE_CD4_TCELL_VS_NATURAL_TREG_UP	0.005509
Immunologic signatures	GSE15215_CD2_POS_VS_NEG_PDC_UP	0.005509
Immunologic signatures	GSE17974_2.5H_VS_72H_IL4_AND_ANTI_IL12_ACT_CD4_TCELL_DN	0.005509
Oncogenic signatures	RB_DN.V1_UP	0.005518
Immunologic signatures	GSE2706_R848_VS_R848_AND_LPS_8H_STIM_DC_UP	0.005568
Oncogenic signatures	GCNP_SHH_UP_EARLY.V1_UP	0.005594
Immunologic signatures	GSE1460_INTRATHYMIC_T_PROGENITOR_VS_THYMIC_STROMAL_CELL_UP	0.005618
Immunologic signatures	GSE1460_INTRATHYMIC_T_PROGENITOR_VS_THYMIC_STROMAL_CELL_DN	0.005618
Immunologic signatures	GSE15750_WT_VS_TRAF6KO_DAY10_EFF_CD8_TCELL_DN	0.005618

Immunologic signatures	GSE17721_LPS_VS_CPG_0.5H_BMDM_DN	0.005618
Immunologic signatures	GSE17721_LPS_VS_CPG_6H_BMDM_DN	0.005618
Immunologic signatures	GSE17721_LPS_VS_CPG_16H_BMDM_DN	0.005618
Immunologic signatures	GSE19825_CD24LOW_VS_IL2RA_HIGH_DAY3_EFF_CD8_TCELL_DN	0.005618
Immunologic signatures	GSE20715_0H_VS_24H_OZONE_LUNG_UP	0.005618
Immunologic signatures	GSE22886_UNSTIM_VS_STIM_MEMORY_TCELL_DN	0.005618
Immunologic signatures	GSE22886_NAIVE_CD4_TCELL_VS_12H_ACT_TH1_DN	0.005618
Immunologic signatures	GSE27786_LIN_NEG_VS_ERYTHROBLAST_DN	0.005618
Immunologic signatures	GSE27786_NKCELL_VS_NEUTROPHIL_UP	0.005618
Immunologic signatures	GSE9650_NAIVE_VS_EFF_CD8_TCELL_DN	0.005618
Immunologic signatures	GSE11864_CSF1_VS_CSF1_IFNG_IN_MAC_UP	0.005719
All canonical pathways	PID_AVB3_INTEGRIN_PATHWAY	0.005869
Oncogenic signatures	VEGF_A_UP.V1_UP	0.005938
Immunologic signatures	GSE14308_TH2_VS_INDUCED_TREG_DN	0.005984
Immunologic signatures	GSE1460_INTRATHYMIC_T_PROGENITOR_VS_NAIVE_CD4_TCELL_ADULT_BLOOD_DN	0.005984
Immunologic signatures	GSE22886_NAIVE_CD4_TCELL_VS_NEUTROPHIL_DN	0.005984
Immunologic signatures	GSE3982_MAST_CELL_VS_BASOPHIL_UP	0.005984
All canonical pathways	REACTOME_G_ALPHA_Z_SIGNALLING_EVENTS	0.006141
Immunologic signatures	GSE15930_NAIVE_VS_72H_IN_VITRO_STIM_CD8_TCELL_DN	0.006177
Immunologic signatures	GSE17721_12H_VS_24H_POLYIC_BMDM_DN	0.006177
Immunologic signatures	GSE20366_TREG_VS_TCONV_DN	0.006177
Immunologic signatures	GSE20366_EX_VIVO_VS_DEC205_CONVERSION_UP	0.006177
Immunologic signatures	GSE22886_NAIVE_BCELL_VS_DC_DN	0.006177
Immunologic signatures	GSE339_CD4POS_VS_CD8POS_DC_DN	0.006177
Immunologic signatures	GSE3982_DC_VS_NKCELL_UP	0.006177
Immunologic signatures	GSE9037_WT_VS_IRAK4_KO_LPS_4H_STIM_BMDM_DN	0.006177
Immunologic signatures	GSE13485_DAY1_VS_DAY21_YF17D_VACCINE_PBMC_DN	0.006313
Immunologic signatures	GSE2706_R848_VS_LPS_2H_STIM_DC_UP	0.006389
Immunologic signatures	GSE22886_NAIVE_TCELL_VS_NKCELL_UP	0.006532
Immunologic signatures	GSE22886_NAIVE_TCELL_VS_MONOCYTE_UP	0.006532
Immunologic signatures	GSE27786_BCELL_VS_NKCELL_DN	0.006532
Immunologic signatures	GSE30962_PRIMARY_VS_SECONDARY_ACUTE_LCMV_INF_CD8_TCELL_DN	0.006532
Immunologic signatures	GSE360_CTRL_VS_B_MALAYI_HIGH_DOSE_MAC_UP	0.006532

Immunologic signatures	GSE360_L_DONOVANI_VS_B_MALAYI_HIGH_DOSE_MAC_UP	0.006532
Immunologic signatures	GSE9988_ANTI_TREM1_VS_ANTI_TREM1_AND_LPS_MONOCYTE_UP	0.006532
All canonical pathways	KEGG_ARGININE_AND_PROLINE_METABOLISM	0.006777
All canonical pathways	REACTOME_FORMATION_OF_TUBULIN_FOLDING_INTERMEDIATES_BY_CCT_TRIC	0.006777
Oncogenic signatures	JNK_DN.V1_UP	0.006778
Oncogenic signatures	PTEN_DN.V2_DN	0.006782
Oncogenic signatures	ATF2_UP.V1_UP	0.006798
Immunologic signatures	GSE26669_CD4_VS_CD8_TCELL_IN_MLR_COSTIM_BLOCK_DN	0.006803
Immunologic signatures	GSE339_CD8POS_VS_CD4CD8DN_DC_UP	0.006803
Immunologic signatures	GSE360_T_GONDII_VS_B_MALAYI_HIGH_DOSE_MAC_DN	0.006803
Immunologic signatures	GSE11864_CSF1_VS_CSF1_PAM3CYS_IN_MAC_UP	0.00698
Immunologic signatures	GSE11057_CD4_EFF_MEM_VS_PBMC_UP	0.007221
Immunologic signatures	GSE1460_NAIVE_CD4_TCELL_ADULT_BLOOD_VS_THYMIC_STROMAL_CELL_UP	0.007221
Immunologic signatures	GSE17721_4H_VS_24H_POLYIC_BMDM_UP	0.007221
Immunologic signatures	GSE17721_0.5H_VS_24H_GARDIQUIMOD_BMDM_UP	0.007221
Immunologic signatures	GSE27786_LIN_NEG_VS_CD4_TCELL_UP	0.007221
Immunologic signatures	GSE27786_CD8_TCELL_VS_NEUTROPHIL_UP	0.007221
Immunologic signatures	GSE3982_DC_VS_EFF_MEMORY_CD4_TCELL_UP	0.007221
Immunologic signatures	GSE25087_FETAL_VS_ADULT_TREG_DN	0.00739
All canonical pathways	REACTOME_RESPONSE_TO_ELEVATED_PLATELET_CYTOSOLIC_CA2_	0.00742
Oncogenic signatures	HOXA9_DN.V1_UP	0.007473
Oncogenic signatures	TBK1.DF_UP	0.007473
Immunologic signatures	GSE20715_0H_VS_6H_OZONE_TLR4_KO_LUNG_UP	0.007486
Immunologic signatures	GSE29618_PDC_VS_MDC_DN	0.007486
Immunologic signatures	GSE7852_LN_VS_FAT_TCONV_DN	0.007486
Immunologic signatures	GSE14000_4H_VS_16H_LPS_DC_DN	0.007694
Immunologic signatures	GSE27786_LIN_NEG_VS_BCELL_DN	0.007694
Oncogenic signatures	LTE2_UP.V1_UP	0.007815
Immunologic signatures	GSE11057_NAIVE_VS_CENT_MEMORY_CD4_TCELL_DN	0.007908
Immunologic signatures	GSE14308_TH17_VS_NAIVE_CD4_TCELL_UP	0.007908
Immunologic signatures	GSE17721_CTRL_VS_CPG_24H_BMDM_UP	0.007908
Immunologic signatures	GSE17721_LPS_VS_PAM3CSK4_4H_BMDM_DN	0.007908
Immunologic signatures	GSE17721_PAM3CSK4_VS_GADIQUIMOD_24H_BMDM_DN	0.007908

Immunologic signatures	GSE24142_ADULT_VS_FETAL_EARLY_THYMIC_PROGENITOR_UP	0.007908
Immunologic signatures	GSE32423_IL7_VS_IL7_IL4_MEMORY_CD8_TCELL_DN	0.007908
Immunologic signatures	GSE360_T_GONDII_VS_B_MALAYI_LOW_DOSE_MAC_UP	0.007908
Immunologic signatures	GSE7460_FOXP3_MUT_VS_WT_ACT_WITH_TGFB_TCONV_UP	0.007908
Immunologic signatures	GSE17721_LPS_VS_GARDIQUIMOD_2H_BMDM_UP	0.008181
Immunologic signatures	GSE22886_DAY0_VS_DAY1_MONOCYTE_IN_CULTURE_UP	0.008181
Immunologic signatures	GSE22886_NAIVE_CD4_TCELL_VS_MONOCYTE_DN	0.008181
Immunologic signatures	GSE28237_FOLLICULAR_VS_EARLY_GC_BCELL_DN	0.008181
All canonical pathways	KEGG_INSULIN_SIGNALING_PATHWAY	0.0083
Oncogenic signatures	ESC_V6.5_UP_EARLY.V1_UP	0.00854
All canonical pathways	PID_RAC1_PATHWAY	0.008667
Immunologic signatures	KAECH_DAY8_EFF_VS_DAY15_EFF_CD8_TCELL_UP	0.008673
Immunologic signatures	GSE12845_IGD_POS_BLOOD_VS_DARKZONE_GC_TONSIL_BCELL_DN	0.008673
Immunologic signatures	GSE15750_WT_VS_TRAF6KO_DAY10_EFF_CD8_TCELL_UP	0.008673
Immunologic signatures	GSE15930_NAIVE_VS_48H_IN_VITRO_STIM_IFNAB_CD8_TCELL_DN	0.008673
Immunologic signatures	GSE17721_LPS_VS_CPG_12H_BMDM_DN	0.008673
Immunologic signatures	GSE20366_CD103_KLRG1_DP_VS_DN_TREG_UP	0.008673
Immunologic signatures	GSE20715_WT_VS_TLR4_KO_6H_OZONE_LUNG_UP	0.008673
Immunologic signatures	GSE20715_0H_VS_6H_OZONE_LUNG_UP	0.008673
Immunologic signatures	GSE28237_EARLY_VS_LATE_GC_BCELL_DN	0.008673
Immunologic signatures	GSE7460_CTRL_VS_TGFB_TREATED_ACT_CD8_TCELL_DN	0.008673
All canonical pathways	PID_IL8CXCR2_PATHWAY	0.008891
Immunologic signatures	GSE9650_GP33_VS_GP276_LCMV_SPECIFIC_EXHAUSTED_CD8_TCELL_UP	0.009007
Oncogenic signatures	CSR_LATE_UP.V1_DN	0.009034
Immunologic signatures	GSE13306_RA_VS_UNTREATED_TREG_DN	0.009251
Immunologic signatures	GSE17721_CPG_VS_GARDIQUIMOD_12H_BMDM_DN	0.009251
Immunologic signatures	GSE19825_NAIVE_VS_IL2RALOW_DAY3_EFF_CD8_TCELL_DN	0.009251
Immunologic signatures	GSE27786_BCELL_VS_CD4_TCELL_DN	0.009251
Immunologic signatures	GSE7460_CD8_TCELL_VS_CD4_TCELL_ACT_DN	0.009251
Immunologic signatures	GSE9037_WT_VS_IRAK4_KO_BMDM_DN	0.009251
All canonical pathways	REACTOME_SYNTHESIS_OF_BILE_ACIDS_AND_BILE_SALTS_VIA_7ALPHA_HYDROXYCHOLEST[illegible]	0.009452
All canonical pathways	REACTOME_CYCLIN_A_B1_ASSOCIATED_EVENTS_DURING_G2_M_TRANSITION	0.009452
Immunologic signatures	GSE1460_INTRATHYMIC_T_PROGENITOR_VS_NAIVE_CD4_TCELL_CORD_BLOOD_DN	0.009469

Immunologic signatures	GSE17721_POLYIC_VS_CPG_6H_BMDM_UP	0.009469
Immunologic signatures	GSE17721_LPS_VS_GARDIQUIMOD_0.5H_BMDM_UP	0.009469
Immunologic signatures	GSE24142_EARLY_THYMIC_PROGENITOR_VS_DN3_THYMOCYTE_ADULT_UP	0.009469
Immunologic signatures	GSE27786_CD4_TCELL_VS_NEUTROPHIL_DN	0.009469
Immunologic signatures	GSE30962_PRIMARY_VS_SECONDARY_CHRONIC_LCMV_INF_CD8_TCELL_UP	0.009469
Immunologic signatures	GSE360_L_MAJOR_VS_B_MALAYI_LOW_DOSE_DC_DN	0.009469
Immunologic signatures	GSE360_L_MAJOR_VS_T_GONDII_MAC_DN	0.009469
Immunologic signatures	GSE9006_1MONTH_VS_4MONTH_POST_TYPE_1_DIABETES_DX_PBMC_UP	0.009469
Immunologic signatures	GSE11864_CSF1_PAM3CYS_VS_CSF1_IFNG_PAM3CYS_IN_MAC_UP	0.009804
All canonical pathways	KEGG_RETINOL_METABOLISM	0.009988
All canonical pathways	BIOCARTA_FIBRINOLYSIS_PATHWAY	0.009988
All canonical pathways	BIOCARTA_LECTIN_PATHWAY	0.009988
All canonical pathways	REACTOME_CELL_CELL_COMMUNICATION	0.009988
All canonical pathways	REACTOME_SIGNALING_BY_PDGF	0.009988
Oncogenic signatures	KRAS.600_UP.V1_UP	0.01001
All canonical pathways	KEGG_STEROID_HORMONE_BIOSYNTHESIS	0.01008
All canonical pathways	REACTOME_GLYCOSAMINOGLYCAN_METABOLISM	0.01008
Immunologic signatures	GSE18791_CTRL_VS_NEWCASTLE_VIRUS_DC_12H_UP	0.01017
Immunologic signatures	GSE25087_TREG_VS_TCONV_ADULT_UP	0.01017
Immunologic signatures	GSE27786_CD8_TCELL_VS_MONO_MAC_DN	0.01017
Immunologic signatures	GSE13306_LAMINA_PROPRIA_VS_SPLEEN_TREG_UP	0.01039
Immunologic signatures	GSE17721_PAM3CSK4_VS_CPG_24H_BMDM_UP	0.01039
Immunologic signatures	GSE17721_POLYIC_VS_CPG_0.5H_BMDM_UP	0.01039
Immunologic signatures	GSE17721_POLYIC_VS_CPG_24H_BMDM_DN	0.01039
Immunologic signatures	GSE26495_NAIVE_VS_PD1LOW_CD8_TCELL_UP	0.01039
Immunologic signatures	GSE29618_MONOCYTE_VS_MDC_DAY7_FLU_VACCINE_UP	0.01039
Immunologic signatures	GSE360_T_GONDII_VS_B_MALAYI_HIGH_DOSE_MAC_UP	0.01039
Immunologic signatures	GSE7852_LN_VS_FAT_TREG_DN	0.01039
Immunologic signatures	GSE8678_IL7R_LOW_VS_HIGH_EFF_CD8_TCELL_DN	0.01039
Oncogenic signatures	WNT_UP.V1_UP	0.01081
Immunologic signatures	GSE16522_ANTI_CD3CD28_STIM_VS_UNSTIM_NAIVE_CD8_TCELL_DN	0.0112
Immunologic signatures	GSE27786_LIN_NEG_VS_NKTCELL_UP	0.0112
Immunologic signatures	GSE29618_PRE_VS_DAY7_POST_TIV_FLU_VACCINE_BCELL_UP	0.0112

All canonical pathways	BIOCARTA_MPR_PATHWAY	0.01125
All canonical pathways	REACTOME_ACTIVATION_OF_THE_PRE_REPLICATIVE_COMPLEX	0.01125
Immunologic signatures	GSE17721_POLYIC_VS_PAM3CSK4_24H_BMDM_DN	0.01157
Immunologic signatures	GSE17721_0.5H_VS_4H_POLYIC_BMDM_UP	0.01157
All canonical pathways	KEGG_GAP_JUNCTION	0.01189
All canonical pathways	REACTOME_SYNTHESIS_OF_BILE_ACIDS_AND_BILE_SALTS	0.01199
All canonical pathways	KEGG_REGULATION_OF_ACTIN_CYTOSKELETON	0.01219
Immunologic signatures	GSE17580_TREG_VS_TEFF_UP	0.01236
Immunologic signatures	GSE17721_CTRL_VS_LPS_1H_BMDM_DN	0.01236
Immunologic signatures	GSE17721_POLYIC_VS_GARDIQUIMOD_0.5H_BMDM_UP	0.01236
Immunologic signatures	GSE17974_CTRL_VS_ACT_IL4_AND_ANTI_IL12_72H_CD4_TCELL_DN	0.01236
Immunologic signatures	GSE18791_CTRL_VS_NEWCASTLE_VIRUS_DC_1H_DN	0.01236
Immunologic signatures	GSE24081_CONTROLLER_VS_PROGRESSOR_HIV_SPECIFIC_CD8_TCELL_UP	0.01236
Immunologic signatures	GSE2706_LPS_VS_R848_AND_LPS_2H_STIM_DC_UP	0.01236
Immunologic signatures	GSE3982_EFF_MEMORY_CD4_TCELL_VS_TH2_UP	0.01236
Oncogenic signatures	PRC2_SUZ12_UP.V1_UP	0.0124
Oncogenic signatures	CYCLIN_D1_KE_.V1_DN	0.01257
All canonical pathways	KEGG_ENDOCYTOSIS	0.01269
Oncogenic signatures	CRX_DN.V1_DN	0.01269
Immunologic signatures	GSE17721_CTRL_VS_GARDIQUIMOD_6H_BMDM_UP	0.01272
Immunologic signatures	GSE20366_CD103_POS_VS_CD103_KLRG1_DP_TREG_DN	0.01272
Immunologic signatures	GSE22886_NEUTROPHIL_VS_MONOCYTE_DN	0.01272
All canonical pathways	KEGG_TIGHT_JUNCTION	0.01367
Immunologic signatures	GSE11864_CSF1_VS_CSF1_IFNG_PAM3CYS_IN_MAC_UP	0.01369
Immunologic signatures	GSE1460_INTRATHYMIC_T_PROGENITOR_VS_CD4_THYMOCYTE_DN	0.01369
Immunologic signatures	GSE17721_LPS_VS_PAM3CSK4_16H_BMDM_UP	0.01369
Immunologic signatures	GSE37416_12H_VS_48H_F_TULARENSIS_LVS_NEUTROPHIL_UP	0.01369
Immunologic signatures	GSE7852_TREG_VS_TCONV_DN	0.01369
Immunologic signatures	GSE22045_TREG_VS_TCONV_UP	0.01372
Immunologic signatures	GSE14769_20MIN_VS_360MIN_LPS_BMDM_DN	0.014
Immunologic signatures	GSE339_EX_VIVO_VS_IN_CULTURE_CD8POS_DC_DN	0.014
Immunologic signatures	GSE3982_NEUTROPHIL_VS_NKCELL_UP	0.014
Immunologic signatures	GSE3982_NEUTROPHIL_VS_TH2_UP	0.014

All canonical pathways	PID_ATR_PATHWAY	0.01414
Immunologic signatures	GSE17974_0.5H_VS_72H_IL4_AND_ANTI_IL12_ACT_CD4_TCELL_DN	0.01416
All canonical pathways	KEGG_PRIMARY_BILE_ACID_BIOSYNTHESIS	0.0145
Oncogenic signatures	STK33_NOMO_DN	0.01453
Immunologic signatures	GSE16522_MEMORY_VS_NAIVE_CD8_TCELL_UP	0.01466
All canonical pathways	KEGG_LEUKOCYTE_TRANSENDOTHELIAL_MIGRATION	0.01486
Oncogenic signatures	RAPA_EARLY_UP.V1_DN	0.01494
Immunologic signatures	GSE17721_LPS_VS_POLYIC_16H_BMDM_UP	0.01494
Immunologic signatures	GSE17721_PAM3CSK4_VS_CPG_6H_BMDM_UP	0.01494
Immunologic signatures	GSE24142_EARLY_THYMIC_PROGENITOR_VS_DN3_THYMOCYTE_ADULT_DN	0.01494
Immunologic signatures	GSE24634_TEFF_VS_TCONV_DAY3_IN_CULTURE_DN	0.01494
Immunologic signatures	GSE24634_IL4_VS_CTRL_TREATED_NAIVE_CD4_TCELL_DAY3_UP	0.01494
Immunologic signatures	GSE29618_BCELL_VS_PDC_DN	0.01494
Immunologic signatures	GSE3337_4H_VS_16H_IFNG_IN_CD8POS_DC_DN	0.01494
Immunologic signatures	GSE7460_WT_VS_FOXP3_HET_ACT_TCONV_UP	0.01494
Oncogenic signatures	CSR_EARLY_UP.V1_UP	0.01512
Immunologic signatures	GSE17974_CTRL_VS_ACT_IL4_AND_ANTI_IL12_2H_CD4_TCELL_UP	0.01565
Oncogenic signatures	ATF2_S_UP.V1_UP	0.01575
Oncogenic signatures	AKT_UP.V1_UP	0.01585
All canonical pathways	KEGG_FC_GAMMA_R_MEDIATED_PHAGOCYTOSIS	0.01606
Immunologic signatures	GSE16522_ANTI_CD3CD28_STIM_VS_UNSTIM_MEMORY_CD8_TCELL_DN	0.01608
Immunologic signatures	GSE17974_0.5H_VS_72H_UNTREATED_IN_VITRO_CD4_TCELL_DN	0.01608
Immunologic signatures	GSE39820_TGFBETA1_VS_TGFBETA3_IN_IL6_TREATED_CD4_TCELL_DN	0.01608
Immunologic signatures	GSE39820_TGFBETA1_VS_TGFBETA3_IN_IL6_IL23A_TREATED_CD4_TCELL_UP	0.01608
Immunologic signatures	GSE17974_IL4_AND_ANTI_IL12_VS_UNTREATED_1H_ACT_CD4_TCELL_DN	0.01617
Immunologic signatures	GSE12845_IGD_NEG_BLOOD_VS_PRE_GC_TONSIL_BCELL_UP	0.01638
Immunologic signatures	GSE17721_CTRL_VS_POLYIC_1H_BMDM_UP	0.01638
Immunologic signatures	GSE17721_CTRL_VS_CPG_8H_BMDM_UP	0.01638
Immunologic signatures	GSE17721_PAM3CSK4_VS_CPG_2H_BMDM_UP	0.01638
Immunologic signatures	GSE22886_TCELL_VS_BCELL_NAIVE_UP	0.01638
Immunologic signatures	GSE360_HIGH_VS_LOW_DOSE_B_MALAYI_MAC_UP	0.01638
All canonical pathways	REACTOME_MRNA_PROCESSING	0.01642
All canonical pathways	REACTOME_INITIAL_TRIGGERING_OF_COMPLEMENT	0.01664

BioCarta gene sets	BIOCARTA_COMP_PATHWAY	0.01746
Immunologic signatures	GSE13229_IMM_VS_MATURE_NKCELL_UP	0.0177
Immunologic signatures	GSE13411_NAIVE_VS_MEMORY_BCELL_DN	0.0177
Immunologic signatures	GSE1432_CTRL_VS_IFNG_6H_MICROGLIA_UP	0.0177
Immunologic signatures	GSE17721_CTRL_VS_CPG_2H_BMDM_UP	0.0177
Immunologic signatures	GSE17721_LPS_VS_GARDIQUIMOD_6H_BMDM_DN	0.0177
Immunologic signatures	GSE17721_ALL_VS_24H_PAM3CSK4_BMDM_DN	0.0177
Immunologic signatures	GSE19825_NAIVE_VS_IL2RAHIGH_DAY3_EFF_CD8_TCELL_UP	0.0177
Immunologic signatures	GSE22886_NAIVE_CD4_TCELL_VS_12H_ACT_TH1_UP	0.0177
Immunologic signatures	GSE24142_ADULT_VS_FETAL_DN3_THYMOCYTE_DN	0.0177
Immunologic signatures	GSE27786_LIN_NEG_VS_ERYTHROBLAST_UP	0.0177
Immunologic signatures	GSE27786_BCELL_VS_ERYTHROBLAST_DN	0.0177
Immunologic signatures	GSE3337_4H_VS_16H_IFNG_IN_CD8POS_DC_UP	0.0177
Immunologic signatures	GSE339_CD4POS_VS_CD8POS_DC_IN_CULTURE_DN	0.0177
Immunologic signatures	GSE360_CTRL_VS_L_DONOVANI_DC_DN	0.0177
Immunologic signatures	GSE360_CTRL_VS_B_MALAYI_HIGH_DOSE_DC_DN	0.0177
Immunologic signatures	GSE360_DC_VS_MAC_L_MAJOR_UP	0.0177
Immunologic signatures	GSE3982_MAST_CELL_VS_NKCELL_DN	0.0177
Immunologic signatures	GSE3982_MAC_VS_TH1_DN	0.0177
Immunologic signatures	GSE3982_NEUTROPHIL_VS_NKCELL_DN	0.0177
Immunologic signatures	GSE7460_CTRL_VS_TGFB_TREATED_ACT_TCONV_UP	0.0177
All canonical pathways	REACTOME_G_BETA_GAMMA_SIGNALLING_THROUGH_PLC_BETA	0.01798
Immunologic signatures	GSE6269_E_COLI_VS_STREP_PNEUMO_INF_PBMC_DN	0.01806
All canonical pathways	REACTOME_PYRUVATE_METABOLISM_AND_CITRIC_ACID_TCA_CYCLE	0.0181
All canonical pathways	REACTOME_CELL_JUNCTION_ORGANIZATION	0.01815
Immunologic signatures	GSE13485_CTRL_VS_DAY1_YF17D_VACCINE_PBMC_UP	0.01864
Immunologic signatures	GSE1460_CD4_THYMOCYTE_VS_NAIVE_CD4_TCELL_ADULT_BLOOD_UP	0.01887
Immunologic signatures	GSE14769_40MIN_VS_360MIN_LPS_BMDM_UP	0.01887
Immunologic signatures	GSE17721_CTRL_VS_CPG_0.5H_BMDM_DN	0.01887
Immunologic signatures	GSE17721_LPS_VS_POLYIC_16H_BMDM_DN	0.01887
Immunologic signatures	GSE17721_POLYIC_VS_CPG_12H_BMDM_UP	0.01887
Immunologic signatures	GSE17721_LPS_VS_GARDIQUIMOD_0.5H_BMDM_DN	0.01887
Immunologic signatures	GSE360_CTRL_VS_M_TUBERCULOSIS_MAC_UP	0.01887

Immunologic signatures	GSE36476_CTRL_VS_TSST_ACT_16H_MEMORY_CD4_TCELL_OLD_UP	0.01887
Immunologic signatures	GSE39820_CTRL_VS_TGFBETA3_IL6_IL23A_CD4_TCELL_DN	0.01887
Immunologic signatures	GSE7460_FOXP3_MUT_VS_WT_ACT_TCONV_UP	0.01887
Immunologic signatures	GSE17721_CTRL_VS_PAM3CSK4_0.5H_BMDM_UP	0.01918
Immunologic signatures	GSE17721_12H_VS_24H_LPS_BMDM_UP	0.01918
Immunologic signatures	GSE20715_WT_VS_TLR4_KO_LUNG_UP	0.01918
Immunologic signatures	GSE339_CD4POS_VS_CD4CD8DN_DC_IN_CULTURE_DN	0.01918
Immunologic signatures	GSE3982_MAST_CELL_VS_TH2_DN	0.01918
Immunologic signatures	GSE3982_NEUTROPHIL_VS_EFF_MEMORY_CD4_TCELL_UP	0.01918
Oncogenic signatures	CAMP_UP.V1_UP	0.0194
Oncogenic signatures	RB_P107_DN.V1_DN	0.0196
Immunologic signatures	GSE17974_0H_VS_4H_IN_VITRO_ACT_CD4_TCELL_UP	0.01968
All canonical pathways	REACTOME_KINESINS	0.01978
All canonical pathways	BIOCARTA_RHO_PATHWAY	0.01989
All canonical pathways	PID_SYNDECAN_4_PATHWAY	0.01989
All canonical pathways	REACTOME_G_PROTEIN_BETA_GAMMA_SIGNALLING	0.01999
Oncogenic signatures	KRAS.600.LUNG.BREAST_UP.V1_UP	0.02029
Oncogenic signatures	CORDENONSI_YAP_CONSERVED_SIGNATURE	0.02039
All canonical pathways	PID_TAP63PATHWAY	0.02049
Immunologic signatures	GSE13229_IMM_VS_MATURE_NKCELL_DN	0.02058
Immunologic signatures	GSE14308_NAIVE_CD4_TCELL_VS_NATURAL_TREG_DN	0.02058
Immunologic signatures	GSE17721_CTRL_VS_PAM3CSK4_24H_BMDM_DN	0.02058
Immunologic signatures	GSE17721_POLYIC_VS_GARDIQUIMOD_2H_BMDM_DN	0.02058
Immunologic signatures	GSE24634_IL4_VS_CTRL_TREATED_NAIVE_CD4_TCELL_DAY7_DN	0.02058
Immunologic signatures	GSE32423_IL7_VS_IL7_IL4_MEMORY_CD8_TCELL_UP	0.02058
Immunologic signatures	GSE32423_IL7_VS_IL7_IL4_NAIVE_CD8_TCELL_UP	0.02058
Immunologic signatures	GSE6269_HEALTHY_VS_STREP_AUREUS_INF_PBMC_DN	0.02058
Immunologic signatures	GSE1432_CTRL_VS_IFNG_24H_MICROGLIA_UP	0.02093
Immunologic signatures	GSE22886_NAIVE_TCELL_VS_MONOCYTE_DN	0.02093
Immunologic signatures	GSE29617_DAY3_VS_DAY7_TIV_FLU_VACCINE_PBMC_2008_DN	0.02093
Immunologic signatures	GSE360_LOW_DOSE_B_MALAYI_VS_M_TUBERCULOSIS_MAC_UP	0.02093
Immunologic signatures	GSE7460_CTRL_VS_TGFB_TREATED_ACT_CD8_TCELL_UP	0.02093
Immunologic signatures	GSE6269_HEALTHY_VS_FLU_INF_PBMC_DN	0.02096

Immunologic signatures	GSE22045_TREG_VS_TCONV_DN	0.02154
Immunologic signatures	GSE22886_CD8_VS_CD4_NAIVE_TCELL_DN	0.02212
Oncogenic signatures	WNT_UP.V1_DN	0.02223
Oncogenic signatures	CAHOY_ASTROCYTIC	0.02223
All canonical pathways	REACTOME_G1_S_SPECIFIC_TRANSCRIPTION	0.02228
All canonical pathways	REACTOME_CLASS_I_MHC_MEDIATED_ANTIGEN_PROCESSING_PRESENTATION	0.02237
Immunologic signatures	GSE11057_CD4_EFF_MEM_VS_PBMC_DN	0.02243
Immunologic signatures	GSE11924_TFH_VS_TH2_CD4_TCELL_DN	0.02243
Immunologic signatures	GSE13306_TREG_RA_VS_TCONV_RA_DN	0.02243
Immunologic signatures	GSE17721_LPS_VS_POLYIC_6H_BMDM_UP	0.02243
Immunologic signatures	GSE339_EX_VIVO_VS_IN_CULTURE_CD4CD8DN_DC_DN	0.02243
Immunologic signatures	GSE360_L_DONOVANI_VS_L_MAJOR_MAC_UP	0.02243
Immunologic signatures	GSE36392_TYPE_2_MYELOID_VS_MAC_IL25_TREATED_LUNG_UP	0.02243
All canonical pathways	PID_ER_NONGENOMIC_PATHWAY	0.02254
Immunologic signatures	GSE17721_CTRL_VS_POLYIC_0.5H_BMDM_DN	0.02289
Immunologic signatures	GSE29614_DAY3_VS_DAY7_TIV_FLU_VACCINE_PBMC_UP	0.02305
Immunologic signatures	GSE22886_IGG_IGA_MEMORY_BCELL_VS_BM_PLASMA_CELL_DN	0.02428
Immunologic signatures	GSE29618_PRE_VS_DAY7_POST_TIV_FLU_VACCINE_BCELL_DN	0.02428
Immunologic signatures	GSE11924_TH1_VS_TH2_CD4_TCELL_UP	0.02453
Immunologic signatures	GSE14350_IL2RB_KO_VS_WT_TREG_UP	0.02453
Immunologic signatures	GSE20715_WT_VS_TLR4_KO_48H_OZONE_LUNG_DN	0.02453
Immunologic signatures	GSE29618_PDC_VS_MDC_DAY7_FLU_VACCINE_UP	0.02453
Immunologic signatures	GSE29618_PRE_VS_DAY7_POST_LAIV_FLU_VACCINE_PDC_DN	0.02453
Immunologic signatures	GSE360_L_MAJOR_VS_T_GONDII_DC_UP	0.02453
Immunologic signatures	GSE39820_CTRL_VS_TGFBETA1_IL6_IL23A_CD4_TCELL_DN	0.02453
Immunologic signatures	GSE7852_LN_VS_THYMUS_TCONV_UP	0.02453
All canonical pathways	PID_E2F_PATHWAY	0.02474
All canonical pathways	REACTOME_REGULATION_OF_INSULIN_SECRETION	0.02476
Immunologic signatures	GSE29617_DAY3_VS_DAY7_TIV_FLU_VACCINE_PBMC_2008_UP	0.02539
All canonical pathways	REACTOME_ADP_SIGNALLING_THROUGH_P2RY12	0.02547
All canonical pathways	BIOCARTA_CLASSIC_PATHWAY	0.02588
All canonical pathways	PID_INTEGRIN2_PATHWAY	0.02588
All canonical pathways	REACTOME_COMMON_PATHWAY	0.02588

All canonical pathways	REACTOME_MITOCHONDRIAL_FATTY_ACID_BETA_OXIDATION	0.02588
All canonical pathways	REACTOME_INHIBITION_OF_INSULIN_SECRETION_BY_ADRENALINE_NORADRENALINE	0.02588
All canonical pathways	REACTOME_E2F_MEDIATED_REGULATION_OF_DNA_REPLICATION	0.02588
Immunologic signatures	GSE11864_UNTREATED_VS_CSF1_PAM3CYS_IN_MAC_UP	0.026
Immunologic signatures	GSE17974_0H_VS_1H_IN_VITRO_ACT_CD4_TCELL_DN	0.026
Immunologic signatures	GSE24102_GRANULOCYSTIC_MDSC_VS_NEUTROPHIL_UP	0.0265
Immunologic signatures	GSE3982_BCELL_VS_BASOPHIL_UP	0.0265
Immunologic signatures	GSE7460_TREG_VS_TCONV_ACT_DN	0.0265
Immunologic signatures	GSE37416_CTRL_VS_0H_F_TULARENSIS_LVS_NEUTROPHIL_DN	0.02651
All canonical pathways	PID_LYSOPHOSPHOLIPID_PATHWAY	0.0266
Immunologic signatures	GSE10239_NAIVE_VS_KLRG1HIGH_EFF_CD8_TCELL_UP	0.02664
Immunologic signatures	GSE10239_MEMORY_VS_DAY4.5_EFF_CD8_TCELL_DN	0.02664
Immunologic signatures	GSE17580_UNINFECTED_VS_S_MANSONI_INF_TREG_DN	0.02664
Immunologic signatures	GSE17721_CTRL_VS_GARDIQUIMOD_12H_BMDM_UP	0.02664
Immunologic signatures	GSE17721_PAM3CSK4_VS_CPG_2H_BMDM_DN	0.02664
Immunologic signatures	GSE17721_PAM3CSK4_VS_GADIQUIMOD_0.5H_BMDM_UP	0.02664
Immunologic signatures	GSE27786_NKTCELL_VS_MONO_MAC_UP	0.02664
Immunologic signatures	GSE30083_SP3_VS_SP4_THYMOCYTE_DN	0.02664
Immunologic signatures	GSE9650_NAIVE_VS_EXHAUSTED_CD8_TCELL_UP	0.02664
Immunologic signatures	GSE14350_IL2RB_KO_VS_WT_TREG_DN	0.02704
Immunologic signatures	GSE30083_SP2_VS_SP3_THYMOCYTE_UP	0.02704
All canonical pathways	KEGG_DRUG_METABOLISM_OTHER_ENZYMES	0.0272
All canonical pathways	REACTOME_SYNTHESIS_OF_DNA	0.02726
Immunologic signatures	GSE10856_CTRL_VS_TNFRSF6B_IN_MACROPHAGE_UP	0.02828
Immunologic signatures	GSE11864_CSF1_IFNG_VS_CSF1_PAM3CYS_IN_MAC_DN	0.02828
Immunologic signatures	GSE17721_0.5H_VS_8H_LPS_BMDM_UP	0.02885
Immunologic signatures	GSE15930_STIM_VS_STIM_AND_TRICHOSTATINA_72H_CD8_T_CELL_DN	0.029
Immunologic signatures	GSE16522_ANTI_CD3CD28_STIM_VS_UNSTIM_MEMORY_CD8_TCELL_UP	0.029
Immunologic signatures	GSE17721_LPS_VS_PAM3CSK4_12H_BMDM_DN	0.029
Immunologic signatures	GSE17721_LPS_VS_GARDIQUIMOD_8H_BMDM_UP	0.029
Immunologic signatures	GSE26669_CD4_VS_CD8_TCELL_IN_MLR_UP	0.029
Immunologic signatures	GSE360_T_GONDII_VS_M_TUBERCULOSIS_DC_UP	0.029
Immunologic signatures	GSE3982_EOSINOPHIL_VS_DC_DN	0.029

Immunologic signatures	GSE5960_TH1_VS_ANERGIC_TH1_DN	0.029
Immunologic signatures	GSE9650_EXHAUSTED_VS_MEMORY_CD8_TCELL_DN	0.029
BioCarta gene sets	BIOCARTA_FIBRINOLYSIS_PATHWAY	0.02909
BioCarta gene sets	BIOCARTA_MPR_PATHWAY	0.02909
BioCarta gene sets	BIOCARTA_LECTIN_PATHWAY	0.02909
All canonical pathways	REACTOME_UNWINDING_OF_DNA	0.02975
All canonical pathways	REACTOME_CREATION_OF_C4_AND_C2_ACTIVATORS	0.03007
All canonical pathways	REACTOME_CHONDROITIN_SULFATE_DERMATAN_SULFATE_METABOLISM	0.03059
Oncogenic signatures	RB_P130_DN.V1_UP	0.03069
All canonical pathways	KEGG_PYRIMIDINE_METABOLISM	0.03081
Immunologic signatures	GSE14308_TH1_VS_INDUCED_TREG_DN	0.03133
Immunologic signatures	GSE1460_CD4_THYMOCYTE_VS_THYMIC_STROMAL_CELL_DN	0.03133
Immunologic signatures	GSE29618_PRE_VS_DAY7_POST_TIV_FLU_VACCINE_PDC_UP	0.03133
Immunologic signatures	GSE32423_CTRL_VS_IL7_MEMORY_CD8_TCELL_DN	0.03133
Immunologic signatures	GSE360_CTRL_VS_B_MALAYI_HIGH_DOSE_DC_UP	0.03133
Immunologic signatures	GSE3982_MAST_CELL_VS_TH1_UP	0.03133
Immunologic signatures	GSE3982_MAC_VS_NEUTROPHIL_UP	0.03133
All canonical pathways	BIOCARTA_MCM_PATHWAY	0.03143
All canonical pathways	KEGG_AXON_GUIDANCE	0.03146
All canonical pathways	REACTOME_DIABETES_PATHWAYS	0.03146
Immunologic signatures	GSE1460_DP_THYMOCYTE_VS_NAIVE_CD4_TCELL_ADULT_BLOOD_UP	0.03167
Immunologic signatures	GSE17721_CPG_VS_GARDIQUIMOD_6H_BMDM_DN	0.03167
Immunologic signatures	GSE17721_PAM3CSK4_VS_GADIQUIMOD_4H_BMDM_DN	0.03167
Immunologic signatures	GSE17721_PAM3CSK4_VS_GADIQUIMOD_16H_BMDM_UP	0.03167
Immunologic signatures	GSE13485_DAY3_VS_DAY7_YF17D_VACCINE_PBMC_UP	0.03252
All canonical pathways	KEGG_BETA_ALANINE_METABOLISM	0.03314
All canonical pathways	PID_CXCR4_PATHWAY	0.03314
All canonical pathways	PID_THROMBIN_PAR1_PATHWAY	0.03314
All canonical pathways	REACTOME_TRANSMISSION_ACROSS_CHEMICAL_SYNAPSES	0.03314
All canonical pathways	REACTOME_REGULATION_OF_WATER_BALANCE_BY_RENAL_AQUAPORINS	0.03314
Immunologic signatures	GSE26928_EFF_MEMORY_VS_CXCR5_POS_CD4_TCELL_DN	0.03323
All canonical pathways	REACTOME_ASSOCIATION_OF_TRIC_CCT_WITH_TARGET_PROTEINS_DURING_BIOSYNTHESIS	0.0336
Immunologic signatures	GSE17721_CTRL_VS_PAM3CSK4_6H_BMDM_DN	0.03435

Immunologic signatures	GSE17721_12H_VS_24H_GARDIQUIMOD_BMDM_DN	0.03435
Immunologic signatures	GSE8515_CTRL_VS_IL1_4H_STIM_MAC_DN	0.03435
Immunologic signatures	GSE20715_0H_VS_48H_OZONE_LUNG_UP	0.0346
Immunologic signatures	GSE360_CTRL_VS_B_MALAYI_LOW_DOSE_MAC_DN	0.0346
Immunologic signatures	GSE36392_TYPE_2_MYELOID_VS_NEUTROPHIL_IL25_TREATED_LUNG_UP	0.0346
Immunologic signatures	GSE9006_HEALTHY_VS_TYPE_1_DIABETES_PBMC_4MONTH_POST_DX_UP	0.0346
Immunologic signatures	GSE9037_WT_VS_IRAK4_KO_LPS_4H_STIM_BMDM_UP	0.0346
All canonical pathways	KEGG_MAPK_SIGNALING_PATHWAY	0.03497
All canonical pathways	KEGG_DRUG_METABOLISM_CYTOCHROME_P450	0.03575
All canonical pathways	KEGG_P53_SIGNALING_PATHWAY	0.03575
All canonical pathways	ST_INTEGRIN_SIGNALING_PATHWAY	0.03575
Immunologic signatures	GSE31082_DN_VS_CD4_SP_THYMOCYTE_UP	0.0372
Immunologic signatures	GSE1460_DP_VS_CD4_THYMOCYTE_UP	0.0375
Immunologic signatures	GSE15767_MED_VS_SCS_MAC_LN_DN	0.0375
Immunologic signatures	GSE17721_LPS_VS_POLYIC_2H_BMDM_DN	0.0375
Immunologic signatures	GSE26495_NAIVE_VS_PD1HIGH_CD8_TCELL_DN	0.0375
Immunologic signatures	GSE39820_CTRL_VS_TGFBETA3_IL6_CD4_TCELL_DN	0.0375
Oncogenic signatures	P53_DN.V1_DN	0.03769
Oncogenic signatures	CAHOY_OLIGODENDROCUTIC	0.03769
Oncogenic signatures	KRAS.PROSTATE_UP.V1_UP	0.03769
Immunologic signatures	GSE32423_CTRL_VS_IL7_IL4_MEMORY_CD8_TCELL_UP	0.03787
Immunologic signatures	GSE39820_CTRL_VS_IL1B_IL6_IL23A_CD4_TCELL_UP	0.03787
All canonical pathways	SA_MMP_CYTOKINE_CONNECTION	0.03849
All canonical pathways	SIG_REGULATION_OF_THE_ACTIN_CYTOSKELETON_BY_RHO_GTPASES	0.03973
Immunologic signatures	GSE12366_PLASMA_CELL_VS_NAIVE_BCELL_UP	0.04017
Immunologic signatures	GSE1460_DP_THYMOCYTE_VS_NAIVE_CD4_TCELL_ADULT_BLOOD_DN	0.04061
Immunologic signatures	GSE1460_NAIVE_CD4_TCELL_CORD_BLOOD_VS_THYMIC_STROMAL_CELL_DN	0.04061
Immunologic signatures	GSE15324_NAIVE_VS_ACTIVATED_ELF4_KO_CD8_TCELL_DN	0.04061
Immunologic signatures	GSE15930_STIM_VS_STIM_AND_IFNAB_72H_CD8_T_CELL_DN	0.04061
Immunologic signatures	GSE17721_CTRL_VS_LPS_4H_BMDM_UP	0.04061
Immunologic signatures	GSE17721_POLYIC_VS_PAM3CSK4_8H_BMDM_UP	0.04061
Immunologic signatures	GSE22886_NAIVE_CD4_TCELL_VS_MEMORY_TCELL_DN	0.04061
Immunologic signatures	GSE29618_BCELL_VS_PDC_DAY7_FLU_VACCINE_DN	0.04061

Immunologic signatures	GSE29618_PRE_VS_DAY7_FLU_VACCINE_BCELL_DN	0.04061
Immunologic signatures	GSE32423_MEMORY_VS_NAIVE_CD8_TCELL_IL7_DN	0.04061
Immunologic signatures	GSE32423_CTRL_VS_IL7_IL4_MEMORY_CD8_TCELL_DN	0.04061
Immunologic signatures	GSE360_L_DONOVANI_VS_L_MAJOR_DC_DN	0.04061
Immunologic signatures	GSE360_HIGH_DOSE_B_MALAYI_VS_M_TUBERCULOSIS_MAC_DN	0.04061
Immunologic signatures	GSE360_CTRL_VS_M_TUBERCULOSIS_DC_DN	0.04094
Immunologic signatures	GSE360_HIGH_DOSE_B_MALAYI_VS_M_TUBERCULOSIS_DC_UP	0.04094
All canonical pathways	KEGG_HYPERTROPHIC_CARDIOMYOPATHY_HCM	0.04182
All canonical pathways	PID_P73PATHWAY	0.04215
All canonical pathways	REACTOME_GPVI_MEDIATED_ACTIVATION_CASCADE	0.04267
Immunologic signatures	GSE17974_0H_VS_6H_IN_VITRO_ACT_CD4_TCELL_UP	0.0429
Immunologic signatures	GSE17721_CTRL_VS_LPS_12H_BMDM_DN	0.04381
Immunologic signatures	GSE17721_CTRL_VS_PAM3CSK4_24H_BMDM_UP	0.04381
Immunologic signatures	GSE17721_POLYIC_VS_PAM3CSK4_16H_BMDM_UP	0.04381
Immunologic signatures	GSE17721_PAM3CSK4_VS_CPG_24H_BMDM_DN	0.04381
Immunologic signatures	GSE17721_12H_VS_24H_PAM3CSK4_BMDM_DN	0.04381
Immunologic signatures	GSE17721_0.5H_VS_4H_GARDIQUIMOD_BMDM_UP	0.04381
Immunologic signatures	GSE22886_NAIVE_CD4_TCELL_VS_DC_UP	0.04381
Immunologic signatures	GSE24142_EARLY_THYMIC_PROGENITOR_VS_DN2_THYMOCYTE_FETAL_UP	0.04381
Immunologic signatures	GSE27786_CD8_TCELL_VS_ERYTHROBLAST_DN	0.04381
Immunologic signatures	GSE27786_ERYTHROBLAST_VS_MONO_MAC_UP	0.04381
Immunologic signatures	GSE29618_PRE_VS_DAY7_POST_LAIV_FLU_VACCINE_MONOCYTE_DN	0.04381
Immunologic signatures	GSE339_CD4POS_VS_CD8POS_DC_IN_CULTURE_UP	0.04381
Immunologic signatures	GSE37416_CTRL_VS_3H_F_TULARENSIS_LVS_NEUTROPHIL_UP	0.04381
Immunologic signatures	GSE3982_BCELL_VS_NKCELL_DN	0.04381
Immunologic signatures	GSE7852_TREG_VS_TCONV_UP	0.04381
All canonical pathways	REACTOME_CITRIC_ACID_CYCLE_TCA_CYCLE	0.04382
Oncogenic signatures	AKT_UP_MTOR_DN.V1_DN	0.04397
Oncogenic signatures	ESC_V6.5_UP_LATE.V1_DN	0.04397
All canonical pathways	PID_IL8CXCR1_PATHWAY	0.04419
All canonical pathways	REACTOME_BILE_ACID_AND_BILE_SALT_METABOLISM	0.04419
All canonical pathways	REACTOME_G_PROTEIN_ACTIVATION	0.04419
Oncogenic signatures	CAMP_UP.V1_DN	0.04423

Oncogenic signatures	NRL_DN.V1_UP	0.04539
All canonical pathways	KEGG_PATHWAYS_IN_CANCER	0.04565
Immunologic signatures	GSE6269_FLU_VS_STREP_PNEUMO_INF_PBMC_DN	0.04583
Oncogenic signatures	ESC_J1_UP_EARLY.V1_DN	0.04629
Immunologic signatures	GSE12845_IGD_POS_BLOOD_VS_NAIVE_TONSIL_BCELL_DN	0.04711
Immunologic signatures	GSE14308_TH2_VS_TH17_DN	0.04711
Immunologic signatures	GSE1460_DP_THYMOCYTE_VS_THYMIC_STROMAL_CELL_DN	0.04711
Immunologic signatures	GSE17721_CTRL_VS_POLYIC_0.5H_BMDM_UP	0.04711
Immunologic signatures	GSE17721_CTRL_VS_PAM3CSK4_2H_BMDM_DN	0.04711
Immunologic signatures	GSE17974_CTRL_VS_ACT_IL4_AND_ANTI_IL12_48H_CD4_TCELL_DN	0.04711
Immunologic signatures	GSE22886_UNSTIM_VS_STIM_MEMORY_TCELL_UP	0.04711
Immunologic signatures	GSE29618_PRE_VS_DAY7_FLU_VACCINE_MDC_DN	0.04711
Immunologic signatures	GSE30083_SP2_VS_SP4_THYMOCYTE_UP	0.04711
Immunologic signatures	GSE32423_MEMORY_VS_NAIVE_CD8_TCELL_UP	0.04711
Immunologic signatures	GSE360_DC_VS_MAC_L_MAJOR_DN	0.04711
Immunologic signatures	GSE36392_MAC_VS_NEUTROPHIL_IL25_TREATED_LUNG_DN	0.04711
Immunologic signatures	GSE3982_DC_VS_TH2_DN	0.04711
Immunologic signatures	GSE7460_CTRL_VS_TGFB_TREATED_ACT_TREG_UP	0.04711
Immunologic signatures	GSE7460_CD8_TCELL_VS_TREG_ACT_UP	0.04711
Immunologic signatures	GSE7460_CTRL_VS_TGFB_TREATED_ACT_FOXP3_HET_TCONV_DN	0.04711
Immunologic signatures	GSE7460_WT_VS_FOXP3_HET_ACT_WITH_TGFB_TCONV_DN	0.04711
Immunologic signatures	GSE7852_LN_VS_THYMUS_TREG_DN	0.04711
Immunologic signatures	GSE9006_HEALTHY_VS_TYPE_1_DIABETES_PBMC_1MONTH_POST_DX_UP	0.04711
All canonical pathways	REACTOME_INSULIN_RECEPTOR_SIGNALLING_CASCADE	0.04735
All canonical pathways	REACTOME_INTEGRIN_CELL_SURFACE_INTERACTIONS	0.04804
Immunologic signatures	GSE26495_PD1HIGH_VS_PD1LOW_CD8_TCELL_DN	0.04872
BioCarta gene sets	BIOCARTA_RHO_PATHWAY	0.04969
